# Endovascular treatment of acute ischemic stroke with a fully radiopaque retriever: A randomized controlled trial

**DOI:** 10.3389/fneur.2022.962987

**Published:** 2022-12-14

**Authors:** Yongxin Zhang, Pei Liu, Zifu Li, Ya Peng, Wenhuo Chen, Liyong Zhang, Jianfeng Chu, Dong Kuai, Zhen Chen, Wei Wu, Yun Xu, Yong Zhang, Bin Zhou, Yu Geng, Congguo Yin, Jiang Li, Ming Wang, Naichi Zhai, Xiaoxiang Peng, Zhong Ji, Yaping Xiao, Xingen Zhu, Xueli Cai, Lei Zhang, Bo Hong, Pengfei Xing, Hongjian Shen, Yongwei Zhang, Minghua Li, Meixia Shang, Jianmin Liu, Pengfei Yang

**Affiliations:** ^1^Neurovascular Center, Changhai Hospital, Naval Military Medical University, Shanghai, China; ^2^Department of Neurosurgery, Changzhou First People's Hospital, Changzhou, China; ^3^Department of Neurology, Zhangzhou Hospital Affiliated to Fujian Medical University, Zhangzhou, China; ^4^Department of Neurosurgery, Liaocheng People's Hospital Brain Hospital, Liaocheng, China; ^5^Department of Neurology, The First People's Hospital of Jining City, Jining, China; ^6^Department of Neurosurgery, Shanxi Provincial Cardiovascular Hospital, Taiyuan, China; ^7^Department of Neurointervention, The First Affiliated Hospital of Zhengzhou University, Zhengzhou, China; ^8^Department of Neurology, Qilu Hospital of Shandong University, Jinan, China; ^9^Department of Neurology, Nanjing Gulou Hospital, Nanjing, China; ^10^Department of Neurology, The Affiliated Hospital of Qingdao University, Qingdao, China; ^11^Department of Neurointervention, Cerebrovascular Disease Center, The Fifth Affiliated Hospital of Sun Yat-sen University, Zhuhai, China; ^12^Department of Neurology, Zhejiang Provincial People's Hospital, Hangzhou, China; ^13^Department of Neurology, Hangzhou First People's Hospital, Hangzhou, China; ^14^Department of Neurosurgery, The Second Affiliated Hospital of Air Force Military Medical University, Xi'an, China; ^15^Department of Neurointervention, Nanyang Second People's Hospital, Nanyang, China; ^16^Department of Neurosurgery, Zibo Central Hospital, Zibo, China; ^17^Department of Neurology, The Third People's Hospital of Hubei Province, Wuhan, China; ^18^Department of Neurology, Nanfang Hospital of Southern Medical University, Guangzhou, China; ^19^Department of Neurology, Shanghai Oriental Hospital, Shanghai, China; ^20^Department of Neurosurgery, The Second Affiliated Hospital of Nanchang University, Nanchang, China; ^21^Department of Neurology, Lishui Municipal Central Hospital, Lishui, China; ^22^Neurovascular Center, Shanghai General Hospital, Shanghai, China; ^23^Institute of Diagnostic and Interventional Neuroradiology, Shanghai Jiao Tong University Affiliated Sixth People's Hospital, Shanghai, China; ^24^Department of Biostatistics, Peking University First Hospital, Beijing, China

**Keywords:** acute ischemic stroke, endovascular treatment, new fully radiopaque retriever, intracranial atherosclerotic disease, a randomized controlled trial

## Abstract

**Objective:**

The Neurohawk retriever is a new fully radiopaque retriever. A randomized controlled non-inferiority trial was conducted to compare the Neurohawk and the Solitaire FR in terms of safety and efficacy. In order to evaluate the efficacy and safety of endovascular treatment in acute ischemic stroke (AIS) caused by intracranial atherosclerotic disease (ICAD) larger vessel occlusion (LVO), a sub-analysis was performed.

**Methods:**

Acute ischemic stroke patients aged 18–80 years with LVO in the anterior circulation were randomly assigned to undergo thrombectomy with either the Neurohawk or the Solitaire FR. The primary efficacy endpoint was successful reperfusion (mTICI 2b-3) rate by the allocated retriever. A relevant non-inferiority margin was 12.5%. Safety outcomes were symptomatic intracranial hemorrhage (sICH) and all-cause mortality within 90 days. Secondary endpoints included first-pass effect (FPE), modified FPE, and favorable outcomes at 90 days. In subgroup analysis, the patients were divided into the ICAD group and non-ICAD group according to etiology, and baseline characteristics, angiographic, and clinical outcomes were compared.

**Results:**

A total of 232 patients were involved in this analysis (115 patients in the Neurohawk group and 117 in the Solitaire group). The rates of successful reperfusion with the allocated retriever were 88.70% in the Neurohawk group and 90.60% in the Solitaire group (95%CI of the difference, −9.74% to 5.94%; *p* = 0.867). There were similar results in FPE and mFPE in both groups. The rate of sICH seemed higher in the Solitaire group (13.16% vs. 7.02%, *p* = 0.124). All-cause mortality and favorable outcome rates were comparable as well. In subgroup analysis, 58 patients were assigned to the ICAD group and the remaining 174 to the non-ICAD group. The final successful reperfusion and favorable outcome rates showed no statistically significant differences in two groups. Mortality within 90 days was relatively lower in the ICAD group (6.90% vs. 17.24%; *p* = 0.054).

**Conclusion:**

The Neurohawk retriever is non-inferior to the Solitaire FR in the mechanical thrombectomy of large vessel occlusion-acute ischemic stroke (LVO-AIS). The sub-analysis suggested that endovascular treatment including thrombectomy with the retriever and essential rescue angioplasty is effective and safe in AIS patients with intracranial atherosclerotic disease-larger vessel occlusion (ICAD-LVO).

**Clinical trial registration:**

https://clinicaltrials.gov/ct2/show/NCT04995757, number: NCT04995757.

## Introduction

Mechanical thrombectomy has become the standard of care for large vessel occlusion-acute ischemic stroke (LVO-AIS) since the five landmark trials were published (1–6). The high reperfusion rates and short procedure shown in these trials were driven by the use of stent-like retrievers. The Solitaire FR (Medtronic Inc., California, USA) has been one of the most frequently used retrievers (7, 8). However, various novel thrombectomy devices with improved visibility have been developed and tested. Although the physical properties of different novel thrombectomy devices have been investigated *in vitro*, the efficacy and safety of any device should also be demonstrated in clinical trials.

**Figure 1:**
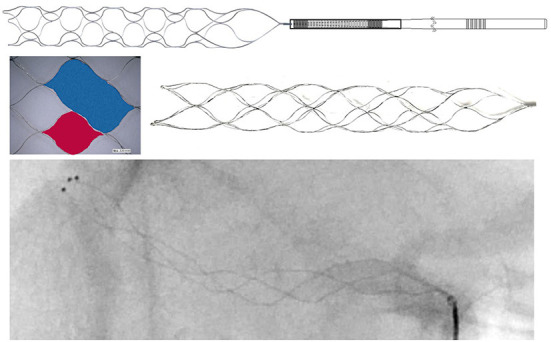
There are three radiopaque marks at the distal end of the Neurohawk retriever. And three radiopaque wires twined around the entire struts. The useable section of the Neurohawk retriever is composed of large cells (blue area) and small cells (red area).

The Neurohawk (MicroPort NeuroTech Company, Shanghai, China) is a stent-like retriever with the closed-cell design. There are three radiopaque marks at the distal end of the retriever. In addition, three radiopaque wires twined around the entire struts of the retriever make it a fully radiopaque device, which allow the physician to visualize the overall placement of the retriever and the combination with thrombus. The useable section of the Neurohawk retriever is composed of large cells and small cells. The large cells is nearly twice as large as the small cells, which is dedicated to catching hard and large-sized thrombus, while the small cells is more conducive to embedding soft and small thrombus (Figure 1). This multi-center randomized controlled trial was designed and carried out to assess the efficacy and safety of this new device comparatively with the Solitaire FR.

As the etiologies of large vessel occlusion were different among ethnic groups, intracranial atherosclerotic disease-larger vessel occlusion (ICAD-LVO) account for a large proportion of patients with acute ischemic stroke (AIS) among Asians. It remains uncertain whether patients with acute ICAD-related occlusion can benefit from mechanical thrombectomy as those with embolism do. Prospective comparative data focusing on efficacy and safety of mechanical thrombectomy between patients with ICAD and those with non-ICAD are scarce. Therefore, a subgroup comparative analysis was conducted.

## Methods

### Study design and patients

This study was a prospective, multicenter, single-blind, randomized, controlled, non-inferiority clinical trial comparing the safety and effectiveness of patients with LVO-AIS treated with either the Neurohawk or the Solitaire FR. The clinical trial followed the principles of law and science, and was approved by the ethics committee at each participating site. This trial planned to enroll 238 patients in 21 tertiary care centers, which were each required to have performed at least 30 endovascular thrombectomy procedures during the previous year.

All patients or their legally authorized representatives provided written informed consent before enrollment. Inclusion criteria were: (1) 18–80 years of age; (2) AIS secondary to internal carotid artery (ICA) or middle cerebral artery (MCA) (M1 or M2) occlusion; (3) ability to undergo puncture within 6 h of symptom onset. Key exclusion criteria were: (1) a pre-stroke modified Rankin Scale (mRS) score (9) ≥2; (2) a baseline National Institutes of Health Stroke Scale (NIHSS) score <2 or >25; (3) massive cerebral infarction defined as an Alberta Stroke Program Early CT Score (ASPECTS) (10) <6 or >1/3 of blood supplying areas on CT/diffusion weighted imaging; (4) concomitant use of oral anticoagulation drugs and INR >3.0, or a platelet count <30 ^*^ 10^9^ /L (11).

### Procedures

According to guidelines, intravenous thrombolysis was administered before mechanical thrombectomy in all eligible patients (12). Depending on the patient's condition, procedures were performed with the patient under local anesthesia, conscious sedation or general anesthesia. The choice of the thrombectomy device was made according to random allocation, which was performed utilizing a 1:1 ratio. Randomization was accomplished by employing a Web-based system with stratification according to each participating site, occlusion segment of artery and the NIHSS. Treatment-group assignment was known to the operating physicians but blinded to the patients. The instructions of the Neurohawk were very similar to those of the Solitaire FR. Due to the presence of the fully radiopaque, the push-and-fluff technique (13), which may lead to better device opening with optimized wall apposition, could be used in the Neurohawk group. Stent retriever combined with aspiration catheter was allowed in both arms. Other retrievers, such as the Trevo (Stryker, Kalamazoo, MI, USA) or other techniques were allowed as salvage measures after unsuccessful recanalization with the Neurohawk or the Solitaire FR. The etiology of occlusion was assessed based on the medical history, risk factors, and angiographic characteristics. For patients with an underlying intracranial stenosis, repeated angiography was performed to exclude potential vasospasm or dissection after the first recanalization. Once atherosclerosis related occlusion was identified, salvage measures, including administration of tirofiban (glycoprotein IIb/IIIa inhibitor) or balloon (Gateway, Boston Scientific, Natick, MA, USA) angioplasty and/or placement of a permanent stent (Enterprise, Johnson and Johnson, Raynham, MA, USA; Wingspan, Stryker, Kalamazoo, MI, United States) or an Apollo balloon mounted stent (MicroPort, Shanghai, China), were allowed (14). Daily oral dual antiplatelet therapy with 100 mg of aspirin and 75 mg of clopidogrel was started post-procedure and continued for 3 months, followed by life-long 100 mg aspirin.

### Outcomes

The primary efficacy endpoint was successful reperfusion rate by the allocated retriever, defined as the percentage of patients achieving modified thrombolysis in cerebral infarction (mTICI) 2b or 3 (15). Secondary endpoints included first-pass effect (FPE, defined as achieving mTICI 3 with a single pass), modified FPE (defined as achieving mTICI 3/2b with a single pass) (16–18), the time from groin puncture to reperfusion, NIHSS at 30 ± 6 h, and favorable clinical outcome (defined as a mRS of 0–2 at 90 ± 14 days). The mRS at 90 days was determined by outpatient follow-up or telephone interview conducted by independent physicians unaware of treatment-group assignment in each center. Safety endpoint measurements were the rate of symptomatic intracranial hemorrhage (sICH) within 30 ± 6 h after intervention, all-cause mortality within 90 days, and all-cause adverse events within 90 days. The sICH was defined as any ICH identified by CT scan combined with a four-point increase in NIHSS or death. Images of the procedure were read by two independent neuroradiologists from the core lab, with consensus required in case of discrepancy. In subgroup analysis, the patients were divided into the ICAD and non-ICAD groups according to the Trial of ORG 10172 in Acute Stroke Treatment (TOAST) classification (19). Baseline characteristics, treatments, and angiographic and clinical outcomes were also compared between the two groups.

### Statistical analysis

The primary study hypothesis was that the successful reperfusion rate of the Neurohawk would be non-inferior to that of the Solitaire FR, with a relevant non-inferiority margin of 12.5%. The non-inferiority margin was calculated by using a two-step method based on the clinical practical significance according to the “Guidelines for the Design of Clinical Trials of Medical Devices” by National Medical Products Administration. All statistical analyses followed the intention-to-treat principle. Baseline data are presented as descriptive statistics according to treatment assignment. The non-inferiority test was based on the asymptotic *Z*-test. Two-sided 95% confidence intervals (CIs) of the differences in the rate of successful reperfusion between the groups were estimated by the Cochran–Mantel–Haenszel chi-squared test with adjusting centers. Statistical tests for continuous variables used Student's *t*-test or the Wilcoxon Mann-Whitney rank-sum test, and categorical variables were tested using the chi-squared test or Fisher's exact test. All statistical tests were two-tailed with a significance level of 0.05, using the SAS software, version 9.4 (SAS Institute, Cary, North Carolina).

## Results

**Figure 2:**
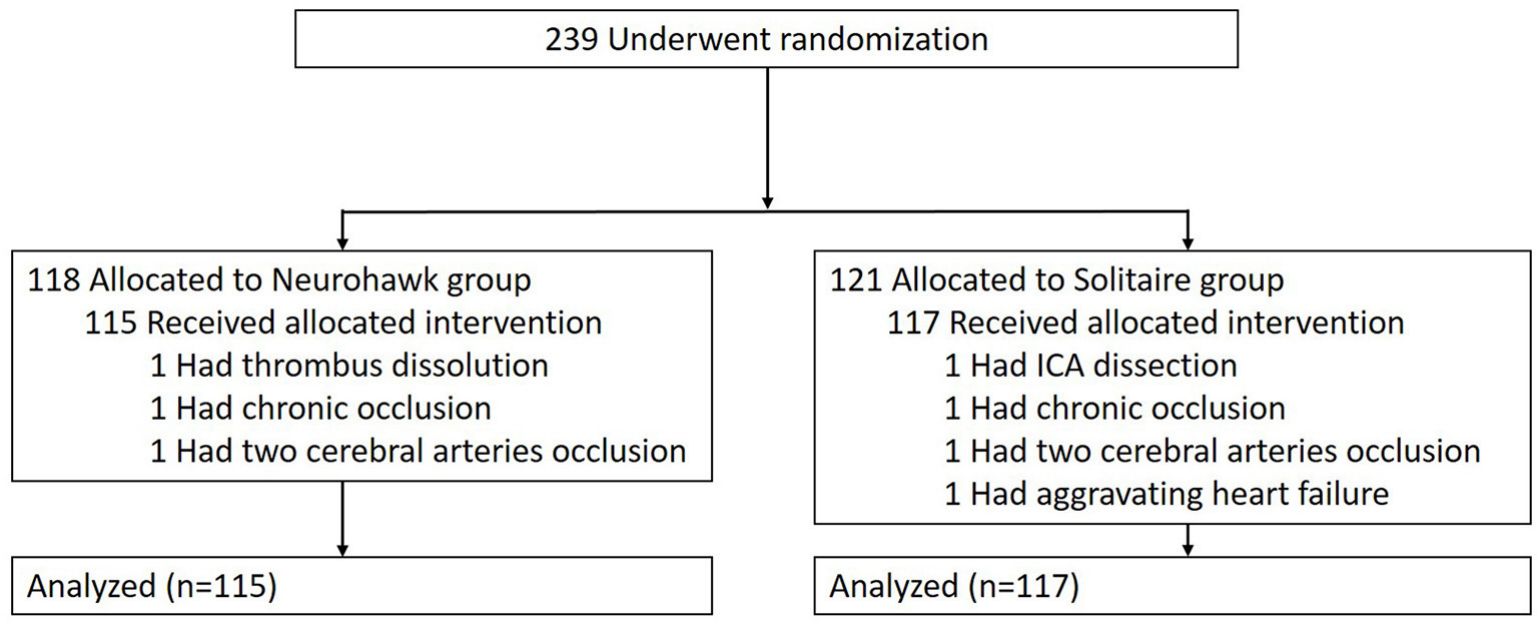
Randomization and treatment of the patients.

A total of 239 patients were enrolled in this trial. A study flow diagram and protocol deviation details are shown in Figure 2. One patient had thrombus dissolution before the retriever reached the target vessel; two patients had chronic occlusion, and one showed aggravating heart failure after general anesthesia. One patient had ICA dissection and two cases had multiple cerebral artery occlusions, which did not align with the inclusion criteria. A total of 115 patients were treated with the Neurohawk, while 117 underwent thrombectomy with the Solitaire FR. Digital subtraction angiographic (DSA) imaging data were missing for one patient in each group. Five patients (two in the Neurohawk group and three in the Solitaire group) were lost to follow-up for mRS assessment at 90 days.

The patient baseline characteristics were similar in both treatment groups, and are detailed in Table 1. The median patient age was 66.6 years. A total of 95 patients had a history of atrial fibrillation and 149 patients had hypertension. Median NIHSS scores were 16 in the Neurohawk group and 17 in the Solitaire group. The median ASPECTS was 8. The most common target vessels were the MCA M1 (117 cases), the ICA (87 cases), and the MCA M2 (26 cases).

**Table 1:** Baseline characteristics of the 232 patients.

**Characteristic**	**Neurohawk group**	**Solitaire group**	***P***
	**(*n* = 115)**	**(*n* = 117)**	
Age (year), median (IQR)	66.5 (57.3, 73.1)	66.6 (58.1, 70.9)	0.654
Male sex, no. (%)	73 (63.48%)	77 (65.81%)	0.710
BMI, mean ± sd	24.7 ± 3.7	24.5 ± 3.2	0.754
**Medical history, no. (%)^a^**			
Previous ischemic stroke	20 (18.20%)	19 (17.00%)	0.959
History of atrial fibrillation	47 (42.73%)	48 (42.86%)	1.000
History of diabetes mellitus	23 (20.91%)	19 (16.96%)	0.453
History of hypertension	77 (70.00%)	72 (64.29%)	0.365
Pre-stroke mRS 1, no. (%)	8 (6.96%)	13 (11.11%)	0.361
NIHSS, median (IQR)	16.0 (13.0, 21.0)	17.0 (13.0, 21.0)	0.853
ASPECTS, median (IQR)^b^	8.0 (7.0, 10.0)	8.0 (7.0, 10.0)	0.311
Time from stroke onset to groin puncture, min, median (IQR)	268.2 (202.2, 319.8)	255.0 (198.0, 315.0)	0.682
**Cause of stroke, no. (%)**			
Cardioembolism	56 (48.70%)	58 (49.57%)	0.335
ICAD	33 (28.70%)	25 (21.37%)	
Undetermined	26 (22.61%)	34 (29.06%)	
**Cerebral arterial occlusion, no. (%)^c^**			
ICA	40 (35.09%)	47 (40.52%)	0.684
MCA M1 segment	61 (53.51%)	56 (48.28%)	
MCA M2 segment	13 (11.40%)	13 (11.20%)	
**Pre-procedure mTICI, no. (%)^c^**			
0	96 (84.21%)	100 (86.21%)	0.678
1	14 (12.28%)	10 (8.62%)	
2a	3 (2.63%)	3 (2.59%)	
2b	1 (0.88%)	3 (2.59%)	
3	0 (0.00%)	0 (0.00%)	

a Data on the medical history were not available for five patients in each group.

b ASPECTS were not available for two patients in the Neurohawk group.

c Data on the arterial occlusion and pre-procedure mTICI were not available for one patient in each group duo to missing of DSA imaging.

Procedural results and outcomes are shown in Table 2. In 92 patients, bridging intravenous fibrinolysis was administrated. General anesthesia was performed in 40.87% of the Neurohawk group and 33.33% of the Solitaire group. There were no differences in bridging therapy and anesthesia method between the two groups. In all 115 patients of the Neurohawk group, the Neurohawk was used as the single retriever with no rescuing needed using other retrievers. The use of a retriever combined with aspiration was similar between the two groups. However, more balloon guide catheters were used in the Solitaire group (*p* = 0.038).

**Table 2:** Procedural and outcomes data.

	**Neurohawk group**	**Solitaire group**	***P***
	**(*n* = 115)**	**(*n* = 117)**	
Bridging intravenous fibrinolysis, no. (%)	40 (34.78%)	52 (44.44%)	0.133
General anesthesia, no. (%)	47 (40.87%)	39 (33.33%)	0.477
Number of passes by retriever, median (IQR)	2.0 (1.0–3.0)	2.0 (1.0–3.0)	0.777
Use of balloon guide catheter	10 (8.70%)	21 (17.95%)	0.038
Retriever combined with aspiration, no. (%)	81 (70.43%)	82 (70.09%)	0.954
Balloon and/or stent angioplasty, no. (%)	27 (23.48%)	24 (20.51%)	0.586
**Primary outcome:**			
Successful reperfusion, no. (%)^a^	102 (88.70%)	106 (90.60%)	0.867
**Major safety outcomes:**			
Symptomatic Intracranial hemorrhage within 36 h, no. (%)^b^	8 (7.02%)	15 (13.16%)	0.124
Death within 90 days, no. (%)	14 (12.17%)	20 (17.09%)	0.289
**Secondary outcomes:**			
mTICI 3 with a first pass, no. (%)^a^	31 (26.96%)	30 (25.64%)	0.820
mTICI 2b/3 with a first pass, no. (%)^a^	49 (42.61%)	44 (37.61%)	0.437
Time from groin puncture to successful reperfusion, min, median (IQR)	58.8 (36.0, 85.2)	52.8 (39.0, 82.8)	0.772
NIHSS at 30 h, median (IQR)	11.0 (5.0, 20.0)	12.0 (5.0, 23.0)	0.316
mRS 0–2 at 90 days, no. (%)^c^	65 (57.52%)	67 (58.77%)	0.849
**Final mTICI, no. (%)^a^**			
0	7 (6.09%)	3 (2.56%)	0.401
1	0 (0.00%)	2 (1.71%)	
2a	4 (3.48%)	5 (4.27%)	
2b	38 (33.04%)	43 (36.75%)	
3	66 (57.39%)	64 (54.71%)	

a DSA imaging data were lost for one patient in each group, the core lab could not evaluate the procedural details, and the mTICI score of patient whose DSA imaging data lost was filled with the worst value—mTICI 0.

b Data on the symptomatic intracranial hemorrhage within 36 h were missing for four patients (one in the Neurohawk group and three in the Solitaire group).

c Data on mRS at 90 days were missing for five patients (two in the Neurohawk group and three in the Solitaire group).

The rates of successful reperfusion with the assigned retriever were 88.70% in the Neurohawk group and 90.60% in the Solitaire group (95%CI of difference, −9.74% to 5.94%; *p* = 0.867). And the median times of pass were two in both group. First-pass complete reperfusion (mTICI 3) was achieved in 61/232 (26.96% vs. 25.64%) and first-pass successful reperfusion (mTICI 3/2b) was achieved in 93/232 (42.61% vs. 37.61%). There were similar FPE and mFPE rates in both groups. Angioplasty with balloon and/or stent use was performed as a remedial measure to maintain a stable flow in 27 patients of the Neurohawk group and 24 of the Solitaire group (*p* = 0.586). Median time from groin puncture to successful reperfusion was similar in both treatment groups (*p* = 0.772). Finally, similar proportions of patients in the Neurohawk and Solitaire groups achieved successful reperfusion (90.43% vs. 91.45%, respectively).

Regarding the major safety outcomes, the rate of sICH within 36 h seemed higher in the Solitaire group (13.16% vs. 7.02%, respectively), but the difference was not statistically significant (*p* = 0.124). All-cause mortality rates within 90 days were 12.17% in the Neurohawk group and 17.09% in the Solitaire group, with no significant difference (*p* = 0.289). There were no significant differences between the two groups in terms of NIHSS at 30 h and favorable outcome (mRS 0–2) at 90 days.

According to the etiology of occlusion, 58 patients were assigned to the ICAD group and the remaining 174 patients to the non-ICAD group. In subgroup analysis, baseline, clinical, and angiographic data between these two groups were compared, and are summarized in Table 3. Compared with the non-ICAD group, individuals with ICAD-LVO had younger age, lower initial NIHSS, and longer time from onset to puncture. Besides, MCA M1 occlusion was more frequent among the patients with ICAD. In terms of treatment method, the proportions of bridging intravenous fibrinolysis were similar in both groups. However, the use of a balloon guide catheter was more preferred in the non-ICAD group (1.72% vs. 17.24%, *p* = 0.003). To maintain a stable flow, 79.31% of patients with ICAD-LVO needed rescue measures. Time from groin puncture to successful reperfusion was longer in the ICAD-LVO group (*p* = 0.004). However, there was no statistically significant difference in successful reperfusion rate between the two groups. In the ICAD group, the successful reperfusion rates were 96.97% in Neurohawk group and 92.0% in Solitaire group (*p* = 0.804). The ICAD group seemed to have a lower frequency of sICH, but without statistical significance. Although mortality within 90 days was relatively lower in the ICAD group (6.90% vs. 17.24%; *p* = 0.054), the rates of favorable outcome at 90 days were comparable between the two groups.

**Table 3:** Baseline, clinical, and angiographic results in subgroup analysis.

	**ICAD group**	**Non-ICAD group**	***P***
	**(*n* = 58)**	**(*n* = 174)**	
Age (year), median (IQR)	63.6 (53.3, 69.9)	67.4 (59.5, 72.5)	**0.021**
Male sex, no. (%)	43 (74.14%)	107 (61.49%)	0.081
NIHSS, median (IQR)	15.0 (11.0, 19.0)	18.0 (14.0, 21.0)	**0.002**
ASPECTS, median (IQR)	8.0 (8.0, 10.0)	8.0 (7.0, 10.0)	0.521
Time from stroke onset to groin puncture (min), median (IQR)	282.6 (214.8, 334.8)	255 (195, 310.2)	**0.024**
Bridging intravenous fibrinolysis, no. (%)	26 (44.83%)	66 (37.93%)	0.352
Cerebral arterial occlusion, no. (%)§			
ICA	19 (32.76%)	68 (39.54%)	**0.022**
MCA M1 segment	37 (63.79%)	80 (46.51%)	
MCA M2 segment	2 (3.45%)	24 (13.95%)	
Use of balloon guide catheter	1 (1.72%)	30 (17.24%)	**0.003**
Rescue measures, no. (%)			
Single balloon angioplasty	13 (22.41%)	0 (0.00%)	
Single stenting	11 (18.97%)	5 (2.87%)	**<0.001**
Balloon and stent angioplasty	22 (37.93%)	0 (0.00%)	
Final successful reperfusion, *n*. (%)^a^	55 (94.83%)	156 (90.70%)	0.476
Symptomatic Intracranial hemorrhage within 36 h, no. (%)^b^	4 (6.90%)	19 (11.18%)	0.350
Time from groin puncture to successful reperfusion, min, median (IQR)	67.2 (46.2, 90.0)	52.8 (35.4, 76.8)	**0.004**
mRS 0–2 at 90 days, no. (%)^c^	37 (64.91%)	95 (55.88%)	0.232
Death within 90 days, no. (%)	4 (6.90%)	30 (17.24%)	**0.054**

a DSA imaging data were lost for two patient in the non-ICAD group, and in sub-analysis, the lost mTICI score wasn't filled with the worst value.

b Data on the symptomatic intracranial hemorrhage within 36 h were missing for four patients in the Non-ICAD group.

c Data on mRS at 90 days were missing for one patient in the ICAD group and four patients in the non-ICAD group.

Bold values of *P* indicate a statistically significant difference.

## Discussion

This randomized controlled trial demonstrated that the Neurohawk achieved comparable rates of successful reperfusion and PFE vs. the Solitaire FR for the treatment of patients with LVO-AIS. From a safety perspective, the rates of sICH were similar. These angiographic results translated to comparable proportions of patients with good clinical outcomes, with close to 60% of patients regaining functional independence at 90 days in each group. In subgroup analysis, almost 80% of individuals with ICAD-LVO needed rescue measures to maintain a favorable reperfusion, including balloon and/or stent angioplasty, which would prolong the time of the procedure. Even so, patients with ICAD-LVO AIS had similar successful reperfusion rate and favorable outcome rate at 90 days vs. those with non-ICAD-LVO AIS. In addition, patients with ICAD-LVO AIS had a relatively lower mortality rate within 90 days.

The Neurohawk retriever is a closed-cell designed retriever with full radiopaque visibility, which can be delivered through a 0.021-inch microcatheter. The available working lengths of the Neurohawk are 25 mm with diameter of 4 mm, and 30 mm with diameter of 6 mm. For a retriever with only several radiopaque marks, physicians could see the ends of the device but not the retrieval area, indicating that they often had to make assumptions regarding the positional relationship between the clot and opened cells. However, the radiopaque Neurohawk retriever allows the physician to visualize the placement of struts at the location of the clot. In addition, it allows a particular deployment maneuver, the push and fluff technique, which may lead to better device opening and optimized wall apposition (20, 21). After partially unsheathing the retriever by retracting the delivery microcatheter, the forward force is applied to the device delivery wire and the forward tension continues to push the device into maximal expansion. Therefore, the larger stent cell area may allow for incorporation of higher volumes of clot.

This study is one of the few randomized controlled trials that focus on the efficacy and safety of a new device for thrombectomy. Although new devices for thrombectomy are constantly emerging, most of them are validated for clinical effects in single-arm studies. Usually, the data from a single group are compared with previous studies of other devices. However, direct comparison of prognoses between single-arm studies and previous registries could produce bias from inhomogeneous baselines and inconsistent operator experiences (22–27). In this study, the prospective randomization design generated a well-balanced baseline between the two groups. From the perspective of angiographic results and clinical outcomes, the Neurohawk was demonstrated to be non-inferior to the Solitaire FR in the treatment of LVO-AIS in the anterior circulation. Although the visibility of Neurohawk may improve the usage experience, the angiographic endpoint showed no difference. One reason might be the high rate of mTICI 2b-3 seen with the modern endovascular technology results in a ceiling effect, making the measurement insensitive. The subtle difference caused by minor improvements may require larger sample sizes and more sensitive measurements to confirm.

While embolism and extracranial atherosclerotic disease is the leading cause of AIS in Caucasian patients, ICAD-LVO cases are more prevalent among Asians (28, 29). In this study, one-fourth of cases resulted from ICAD-LVO. The characteristics of occlusions arising from ICAD and non-ICAD differed in terms of therapeutic responses. For instance, subsequent plaque irritation and platelet aggregation are persistent even after mechanical thrombectomy, which often leads to re-occlusion. Another concern for these patients is whether the use of antiplatelet drugs post-angioplasty would increase the risk of hemorrhagic complications. However, limited studies have so far assessed the efficacy and safety of mechanical thrombectomy in AIS due to ICD-LVO, and the only information available is based on single center, retrospective studies conducted on few samples (14, 30–32).

In this study, patients with ICAD-LVO had lower initial NIHSS, which might be attributed to ischemic preconditioning and better collateral compensation. This finding was in line with the EAST study in which admission NIHSS in the ICAD group was lower than that of the embolic group (33). Endovascular treatment of AIS underlying ICAD-LVO is technically more complex. About four-fifth of patients with ICAD-LVO received angioplasty, while only five patients in the non-ICAD group needed single stent implantation. The EAST study in China showed 63.8% (30/47) of patients were considered to be eligible for rescue treatment. In our previous meta-analysis of endovascular treatment of ICAD-LVO, the most common rescue therapy was stenting with or without balloon angioplasty (32.7%), followed by single balloon angioplasty (12.3%) (30). Besides, our data showed additional rescue therapy was indeed reflected by significantly longer procedure time, in concordance with the previous study. Finally, our findings corroborate studies that also demonstrated similar angiographic and clinical outcomes were obtained in the treatment of acute ICAD-LVO.

This study had several underlying limitations because of the restrictive nature of the randomized controlled non-inferiority trial design, including the limited sample size, and strict inclusion and exclusion criteria. In addition, the mRS was accessed by physicians in each center, which can lead to heterogenous judgments. The sub-analysis also had several limitations. Data for this sub-analysis were derived from the original trial, and therefore, selection bias was inevitable. There was heterogeneity in the intraoperative antiplatelet regimen among different centers.

## Conclusion

This randomized clinical trial demonstrated that the Neurohawk retriever is non-inferior to the Solitaire FR in the mechanical thrombectomy of LVO-AIS. Meanwhile, the sub-analysis suggested that endovascular treatment including thrombectomy with the retriever and essential rescue angioplasty is effective and safe in patients with AIS underlying ICAD-LVO.

## Data availability statement

The original contributions presented in the study are included in the article/Supplementary material, further inquiries can be directed to the corresponding authors.

## Ethics statement

The studies involving human participants were reviewed and approved by the Ethics Committee at each participating site (the full list is available as a Supplementary file). The patients/participants provided their written informed consent to participate in this study.

## Author contributions

PY and JLiu: conceptualization, responsible for theoretical guidance and conceptual specification, methodology, funding acquisition, project research fund, data collection and collation, and supervision. PY, JLiu, YongxZ, PL, MS, and ML: responsible for the guidance of research methods, formal analysis, and investigation. YongxZ and PL: analyze the current research status and put forward the research direction, writing—original draft preparation, and writing—review and editing. YongxZ, PL, ZL, YP, WC, LiZ, JC, DK, ZC, WW, YXu, YongZ, BZ, YG, CY, JLi, MW, NZ, XP, ZJ, YXi, XZ, XC, LeZ, BH, PX, HS, and YongwZ: resources. All authors read and approved the final manuscript.

## Funding

This study was funded by MicroPort NeuroTech (Shanghai, China). The funder was not involved in the study design, collection, analysis, interpretation of data, the writing of this article, or the decision to submit it for publication.

## Conflict of interest

The authors declare that the research was conducted in the absence of any commercial or financial relationships that could be construed as a potential conflict of interest.

## Publisher's note

All claims expressed in this article are solely those of the authors and do not necessarily represent those of their affiliated organizations, or those of the publisher, the editors and the reviewers. Any product that may be evaluated in this article, or claim that may be made by its manufacturer, is not guaranteed or endorsed by the publisher.
