# Supplementary material for: Endovascular treatment of acute ischemic stroke with a fully radiopaque retriever: A randomized controlled trial
Source: Front Neurol. 2022 Dec 14;13:962987. doi: 10.3389/fneur.2022.962987 (PMC9796564; doi:10.3389/fneur.2022.962987)
Supplement: Supplementary file 1 [file Data_Sheet_1.zip › 01 │ñ║ú .pdf]

上海长海医院伦理委员会批准函

Approval Letter

附件 ☑ 有 ☐ 无

方案受理编号:	2017-041	☐ 快审<br>☑ 会议审查	审查日期<br>Date: 2017-04-19
伦理批件编号:	CHEC2017-078		会议编号:<br>Meeting No.:<br>M2017-004-2017-04-19
研究方案全称/简称或编号<br>Protocol Title/ Number:	取栓器治疗急性缺血性卒中的前瞻性、多中心、单盲、随机对照临床试验,项目编号: 2016-GATOR-01-A		
申办者 Sponsor: 微创神通医疗科技(上海)有限公司/CRO:方恩(天津)医药发展有限公司	试验产品名称 Study product:: NA	CFDA 批件号 Approval Number by CFDA: NA	
本院主要研究者/科室 PI/site	刘建民/神经外科		
组长单位名称	上海长海医院		

审查决定(在☐内划×)Decision for this proposal:

☑ 同 意 Approved　　☐ 修改后同意 Approved with Recommendation

☐ 不同意 Disapproved　　☐ 终止或暂停试验 Termination/Suspension

跟踪/持续审查频率:本项目首次批准后每 12 月递交进展报告;

研究需按照伦理委员会审查批准的方案和知情同意书进行,具体说明见第 2 页。

受理审查文件(含版本号)如下: List of reviewed documents with Version No.

1. 研究方案: 版本号 V1.0, 日期 2017 年 03 月 08 日;
2. 知情同意书: 版本号 V1.0, 日期 2017 年 03 月 08 日;
3. 病例报告表; 版本号 V1.0, 日期 2017 年 03 月 08 日;
4. 原始病历: 版本号 V1.0, 日期 2017 年 03 月 08 日;
5. 研究者手册: 版本号 V1.0, 日期 2017 年 03 月 08 日;
6. 医疗器械产品检测报告;
7. 医疗器械产品技术要求;
8. 受试产品说明书;
9. 招募说明: 日期 2017 年 03 月 10 日。

主任委员签名: [signature]

上海长海医院伦理委员会(盖章):

日 期 : 2017 年 4 月 24 日

地址: 上海市杨浦区长海路 168 号 Address: No. 168, Changhai Road, Yangpu District, Shanghai, China
电话&传真 Tel & Fax: 86-21-31162338, 邮政编码: postcode:200433 邮箱 Email:changhaiec@126.com

声 明

1. 上海长海医院伦理委员会的职责、人员组成、操作规程和记录遵循中华人民共和国食品药品监督管理局颁布的药物临床质量管理规范（GCP）和 ICH GCP 的伦理审查原则，并遵守中国相关法律和法规的规定。

2. “同意”的研究应遵循已经 CHEC 批准的方案执行，应符合 CFDA/GCP 和赫尔辛基宣言的原则。conduct the research in accordance with the protocol, CFDA/GCP and the principles of research ethics as set forth in the Declaration of Helsinki.

3. “不同意”和“暂停或终止”的研究方案，申办者和研究者可就 CHEC 的意见和建议中提及的问题做书面申诉，并陈述理由。CHEC 可就申诉作重新审查。If you wish to appeal to this decision, please contact the IEC of Shanghai Changhai Hospital and submit your appeal in writing, addressed to the IEC Chairperson with justification as to why the appeal should be granted.

4. 研究过程中，对研究方案和知情同意书等相关文件所作的任何修改，均需得到伦理委员会审查同意后方可实施。Any change to the protocol or informed consent or other attachments should be approved by IEC before being implemented.

5. 本中心发生的严重不良事件或非预期不良事件需在向 CFDA 上报的同时向 CHEC 作书面通报，CHEC 有权根据对其评估做出新的决定。Report all the SAE and unexpected AE to CHEC. Base on the review result, CHEC will give PI a new recommendation and decision.

6. 本伦理委员会按照国家有关规定，对研究项目进行跟踪审查。自批准函生效日起，请研究者在规定的持续审查日到期或批准函失效期前 1 个月递交进展报告，以获得伦理委员会的批准。Please resubmit this proposal for a Continuing Review at least 1 months before the next re-approval period.

7. 研究结束时，请向伦理委员会递交结题报告和分中心小结表。

地址：上海市杨浦区长海路 168 号 Address: No. 168, Changhai Road, Yangpu District, Shanghai, China
电话&传真 Tel & Fax: 86-21-31162338, 邮政编码：postcode:200433 邮箱 Email:changhaiec@126.com

上海长海医院伦理委员会批准函附件

方案受理编号:	2017-041	审查日期<br>Date: 2017-04-19
伦理批件编号:	CHEC2017-078	会议编号:<br>Meeting No.:<br>M2017-004-2017-04-19
研究方案全称/简称或编号<br>Protocol Title/ Number:	取栓器治疗急性缺血性卒中的前瞻性、多中心、单盲、随机对照临床试验,项目编号: 2016-GATOR-01-A	
申办者 Sponsor: 微创神通医疗科技(上海)有限公司/CRO:方恩(天津)医药发展有限公司	试验产品名称 Study product:: NA	CFDA 批件号 Approval Number by CFDA: NA
本院主要研究者/科室 PI/site	刘建民/神经外科	
组长单位名称	上海长海医院	

审查决定过程:
1. 会议审查:时间 2017 年 04 月 19 日 参会委员人数 10 人
评审结果:同意 10 人。
审查决定
☑ 同 意 ☐ 修改后同意 ☐ 不同意 ☐ 终止或暂停试验

上海长海医院伦理委员会(盖章):
(Stamp: 上海长海医院医学伦理委员会 业务专用章)

日 期 :2017 年 4 月 24 日

IEC 会议记录表
Form of IEC Meeting Minutes

会议编号：Meeting No:	M2017-004 ■ 例行会议 Regular meeting ☐ 紧急会议 Emergency meeting
日期：Date（YY/MM/DD）：	2017-04-19
地点：Location:	长海医院伦理委员会办公室（科技楼 202）
开始/结束时间：Starting/Adjourned time:	14:00-18:00
会议主席：Chairman:	刘燕敏

参加会议的 IEC 委员及列席者名单 Attending IEC members and guests:

序号 No.	任职 Position	姓名 Name	性别 Gender	专业 Professional	工作单位 Institution	签名 Signature
1	主任委员	刘燕敏	女	管　理	长海医院院办	[illegible]
2	副主任委员	蔡全才	男	流行病学	长海医院流行病学中心	[illegible]
3	委　员	徐志云	男	心胸外科	长海医院心胸外科	[illegible]
4	委　员	韩一平	女	呼　吸	长海医院呼吸科	[illegible]
5	委　员	杨建民	男	血　液	长海医院血液科	[illegible]
6	委　员	陆清声	男	血管外科	长海医院血管外科	[illegible]
7	委　员	马丽萍	女	心　内	长海医院心内科	[illegible]
8	委　员	印慨	男	普外科	长海医院普外科	[illegible]
9	委　员	陈志辉	男	感　染	长海医院感染科	[illegible]
10	委　员	束学安	男	法　律	北京盈科（上海）律师事务所	[illegible]

上海长海医院伦理委员会

Shanghai Changhai Hospital Ethics Committee

上海长海医院伦理委员会批准函

Approval Letter

附件 ☐ 有 ☑ 无

方案受理编号：	2017-041A	☑ 快审	审查日期 Date: 2018-09-18
伦理批件编号：	CHEC2018-124	☐ 会议审查	会议编号：Meeting No.：NA
研究方案全称/简称或编号 Protocol Title/ Number:	取栓器治疗急性缺血性卒中的前瞻性、多中心、单盲、随机对照临床试验，项目编号：2016-GATOR-01-A		
申办者 Sponsor：微创神通医疗科技（上海）有限公司/CRO: 方恩（天津）医药发展有限公司	试验产品名称 Study product:: NA	CFDA 批件号 Approval Number by CFDA：NA	
本院主要研究者/科室 PI/site	刘建民/神经外科		
组长单位名称	上海长海医院		

审查决定（在☐内划√）Decision for this proposal:

☑ 同 意　☐ 修改后同意　☐ 修改后重审　☐ 不同意　☐ 终止或暂停试验

跟踪/持续审查频率：本项目首次批准后每 12 月递交进展报告；

研究需按照伦理委员会审查批准的方案和知情同意书进行，具体说明见第 2 页。

受理审查文件（含版本号）如下： List of reviewed documents with Version No.

1. 研究方案：版本号 V2.0，日期 2018 年 08 月 08 日；
2. 知情同意书：版本号 V2.0，日期 2018 年 08 月 08 日；
3. 研究者手册：版本号 V2.0，日期 2018 年 08 月 08 日；
4. 病例报告表：版本号 ~~V2.0~~ 3.0，日期 2018 年 08 月 08 日；
5. 原始病历：版本号 ~~V2.0~~ 3.0，日期 2018 年 08 月 08 日；
6. 取栓器使用说明：版本号 Rev2.0。

JYP 2018-9-25

主任委员签名：

上海长海医院伦理委员会（盖章）：

日 期 ：2018 年 9 月 25 日

地址：上海市杨浦区长海路 168 号　Address: No. 168, Changhai Road, Yangpu District, Shanghai, China
电话&传真 Tel & Fax：86-21-31162338，　邮政编码：postcode:200433　邮箱 Email:changhaiec@126.com

声 明

1. 上海长海医院伦理委员会的职责、人员组成、操作规程和记录遵循中华人民共和国食品药品监督管理局颁布的药物临床质量管理规范（GCP）和 ICH GCP 的伦理审查原则，并遵守中国相关法律和法规的规定。

2. “同意”的研究应遵循已经 CHEC 批准的方案执行，应符合 CFDA/GCP 和赫尔辛基宣言的原则。conduct the research in accordance with the protocol, CFDA/GCP and the principles of research ethics as set forth in the Declaration of Helsinki.

3. “不同意”和“暂停或终止”的研究方案，申办者和研究者可就 CHEC 的意见和建议中提及的问题做书面申诉，并陈述理由。CHEC 可就申诉作重新审查。If you wish to appeal to this decision, please contact the IEC of Shanghai Changhai Hospital and submit your appeal in writing, addressed to the IEC Chairperson with justification as to why the appeal should be granted.

4. 研究过程中，对研究方案和知情同意书等相关文件所作的任何修改，均需得到伦理委员会审查同意后方可实施。Any change to the protocol or informed consent or other attachments should be approved by IEC before being implemented.

5. 本中心发生的严重不良事件或非预期不良事件需在向 CFDA 上报的同时向 CHEC 作书面通报，CHEC 有权根据对其评估做出新的决定。Report all the SAE and unexpected AE to CHEC. Base on the review result, CHEC will give PI a new recommendation and decision.

6. 本伦理委员会按照国家有关规定，对研究项目进行跟踪审查。自批准函生效日起，请研究者在规定的持续审查日到期或批准函失效期前 1 个月递交进展报告，以获得伦理委员会的批准。Please resubmit this proposal for a Continuing Review at least 1 months before the next re-approval period.

7. 研究结束时，请向伦理委员会递交结题报告和分中心小结表。

地址：上海市杨浦区长海路 168 号 Address: No. 168, Changhai Road, Yangpu District, Shanghai, China
电话&传真 Tel & Fax：86-21-31162338， 邮政编码：postcode:200433 邮箱 Email:changhaiec@126.com
