# Supplementary material for: Endovascular treatment of acute ischemic stroke with a fully radiopaque retriever: A randomized controlled trial
Source: Front Neurol. 2022 Dec 14;13:962987. doi: 10.3389/fneur.2022.962987 (PMC9796564; doi:10.3389/fneur.2022.962987)
Supplement: Supplementary file 1 [file Data_Sheet_1.zip › 02 ╓ú┤≤╥╗.pdf]

# 郑州大学第一附属医院科研/药物临床试验伦理委员会伦理审查批件

<table>
<tr><td>伦理批准编号</td><td colspan="3">械-2017-15</td></tr>
<tr><td>研究方案名称</td><td colspan="3">取栓器治疗急性缺血性卒中的前瞻性、多中心、单盲、随机对照临床试验</td></tr>
<tr><td>申办者</td><td>微创神通医疗科技（上海）有限公司</td><td>CFDA 批件</td><td>□有 □无 ■不适用</td></tr>
<tr><td>CRO</td><td>方恩（天津）医药发展有限公司</td><td rowspan="2">研究期别</td><td rowspan="2">□Ⅰ期 □Ⅱ期 □Ⅲ期 □Ⅳ期 □登记研究<br>□临床科研 ■器械临床验证□器械临床试用</td></tr>
<tr><td>试验器械名称及型号<br>药品名称及剂型</td><td>取栓器/AIS4025、AIS6030</td></tr>
<tr><td>报审资料及内容</td><td colspan="3">试验方案（2016-GATOR-01-A 版本号：V1.0，版本日期：20170308）；郑州大学第一附属医院知情同意书（版本号：2016-GATOR-01-1E V1.0，版本日期：20170405）；郑州大学第一附属医院知情同意书（2016-GATOR-01-1E 版本号：V2.0，版本日期：20170626）；受试者招募说明；病例报告表（2016-GATOR-01-F 版本号：V1.0，版本日期：20170308）；原始病历（2016-GATOR-01-I 版本号：V1.0，版本日期：20170308）；研究者手册（2016-GATOR-01-G 版本号：V1.0，版本日期：20170308）；取栓器及对照产品说明书；临床试验机构的设施条件能够满足试验的综述；主要研究者履历及团队成员名单；企业法人营业执照；医疗器械生产企业许可证；复核通过的医疗器械注册产品标准；样品检测报告及自测报告；试验用医疗器械的研制符合适用的医疗器械质量管理体系相关要求的声明；组长单位伦理批件；组长单位知情同意书；CRO 授权委托书；CRO 资质；保险；等。</td></tr>
<tr><td>主要研究者及职称</td><td>管生/主任医师</td><td>研究单位及专业</td><td>郑州大学第一附属医院神经介入科</td></tr>
<tr><td>首次审查方式</td><td colspan="3">■会议审查 □快速审查 □紧急会议审查</td></tr>
<tr><td>参会情况</td><td colspan="3">委员人数 10 人；出席人数 9 人；请假 1 人；回避 0 人。</td></tr>
<tr><td colspan="4">投票情况 同意 4 票；修改后同意 5 票；修改后快速审查 0 票；修改后会议审查 0 票；不同意 0 票；暂停或终止 0 票；终止 0 票</td></tr>
<tr><td>首次会议审查决定</td><td colspan="3">□同意；■修改后同意；□修改后快速审查；□修改后会议审查；□不同意；□终止或暂停试验</td></tr>
</table>

根据 2017 年 06 月 26 日郑州大学第一附属医院科研/药物临床试验伦理委员会伦理会议审查所提出的问题，2017 年 07 月 11 日微创神通医疗科技（上海）有限公司向郑州大学第一附属医院科研/药物临床试验伦理委员会递交了补充修改后的临床试验伦理审查资料，郑州大学第一附属医院科研/药物临床试验伦理委员会对该公司补充修改后送审材料进行审查和讨论，**本次审批意见如下：同意在我院进行该临床试验研究。**

1、请按照 GCP 原则和会议审查通过的递交材料：试验方案（2016-GATOR-01-A 版本号：V1.0，版本日期：20170308）；郑州大学第一附属医院知情同意书（2016-GATOR-01-1E 版本号：V2.0，版本日期：20170626）进行临床试验研究。

2、在开展该项研究时要求，上述审评资料未经本委员会批准，不得作任何修改；如果研究中发生严重不良事件请立即（24 小时内）通知本委员会。

3、如果试验开展一年以上，需向本委员会提交试验年度报告并接受年度跟踪审查及再备案。

4、该研究进行过程中将接受伦理委员会的持续审查，伦理委员会有权根据实际进展情况改变持续审查频率。

5、批件超过有效期请提前 1 个月提出申请。

主任委员签字：

（伦理委员会盖章）

2017 年 07 月 13 日

| | | |
|---|---|---|
| 年度/定期跟踪审查频率：□3 个月 □6 个月 ■12 个月 | 此批件有效期至：2018 年 07 月 13 日 | |
| 会议审查地点：郑州大学第一附属医院 15 号楼 B 楼 5 层会议室 | 会议秘书：赵菁 | 首次会议审查时间：2017-06-26 |

声明：

1.本院伦理委员会职责、人员组成，操作规程及记录均遵循 ICH-GCP、CFDA-GCP、中国相关法规。

2.“同意”的研究应遵循已经伦理委员会批准的方案执行，应符合 ICH-GCP、CFDA-GCP 和《赫尔辛基宣言》的原则。

3.“修改后同意”的研究请按评审意见进行逐条修改并递交临床研究文件修正申请（AF15）及新版本文件，申请经伦理委员会同意后，方可获得批准编号。

4.“修改后快速审查”的研究请按评审意见进行修改并递交临床研究文件修正申请（AF16）及新版本文件，该申请会出 2 名主审委员审查，根据主审意见决定结果。

5. “修改后会议审查”的研究请按评审意见进行修改，并按研究者送审通知（AF06）重新递交新版本文件，在下次全体会议中审查，由全体委员投票决定结果。

6.“不同意”或“暂停或终止”的研究方案，申办者和研究者有权就伦理委员会的意见和建议中提及的问题做书面申诉，由全体委员投票决定结果。

7.本中心发生的严重不良事件或意外不良事件需在向 CFDA 上报的同时向伦理委员会作书面报告，必要时会邀请研究者参加全体会议进行审查，伦理委员会有权根据对其的评估并做出新的决定。

8.无论试验开始与否，请在下次持续审查已到期前 1 个月提出持续审查的申请。

9.郑州大学第一附属医院科研/药物临床试验伦理委员会

地址：郑州市建设东路 1 号郑州大学第一附属医院，邮编：450052

秘书处联系人：赵菁 联系电话：13937175368

E-mail: zhaojingyxj@126.com

# 郑州大学第一附属医院科研和临床试验伦理委员会

# 修正案审查伦理批件

| | | | |
|---|---|---|---|
| 方案修正<br>伦理批准编号 | 修正案-2018-124 | | |
| 首次伦理审查<br>批准编号 | 械-2017-15 | 首次伦理审查<br>批准日期 | 2017-07-13 |
| 研究方案名称 | 取栓器治疗急性缺血性卒中的前瞻性、多中心、单盲、随机对照临床试验 | | |
| 申办者 | 微创神通医疗科技（上海）有限公司 | CFDA 批件 | □有 □无 ■不适用 |
| CRO | 方恩（天津）医药发展有限公司 | 研究期别 | □Ⅰ期 □Ⅱ期 □Ⅲ期 □Ⅳ期 □登记研究<br>□临床科研 ■器械临床验证 □器械临床试用 |
| 试验器械名称及型号 | 取栓器/AIS4025、AIS6030 | | |
| 修改资料及内容 | 1、临床试验方案版本 V2.0 说明。2、方案修改对照表。3、方案 V2.0/20180808（痕迹版及无痕版）。4、知情同意书修改对照表。5、郑州大学第一附属医院专用版知情同意书 V3.0/20180926（痕迹版及无痕版）。6、研究者手册修改对照表。7、研究者手册 V2.0/20180808（痕迹版及无痕版）。8、病例报告表修改对照表。9、病例报告表 V3.0/20180808（痕迹版及无痕版）。10、原始病历修改对照表。11、原始病历 V3.0/20180808（痕迹版及无痕版）。12、取栓器说明书修改对照表。13、取栓器说明书 V2.0（痕迹版及无痕版）。14、组长单位伦理批件。 | | |
| 主要研究者及职称 | 管生/主任医师 | 研究单位及专业 | 郑州大学第一附属医院神经介入科 |
| 审查方式 | □会议审查 ■快速审查 □紧急会议审查 | | |

2018 年 10 月 19 日微创神通医疗科技（上海）有限公司向郑州大学第一附属医院科研和临床试验伦理委员会递交了方案修改后资料，郑州大学第一附属医院科研和临床试验伦理委员会对该公司方案修改后送审材料进行审查和讨论，**本次审批意见如下：同意在我院按照新版本试验方案进行该临床试验研究。**

1、请按照 GCP 原则和审查通过的递交材料：试验方案（版本号：V2.0，版本日期：20180808）、知情同意书（版本号：V3.0，版本日期：20180926）进行临床试验研究。

2、在开展该项研究时要求，上述审评资料未经本委员会批准，不得作任何修改；如果研究中发生严重不良事件请立即（24 小时内）通知本委员会。

3、如果试验开展一年以上，需向本委员会提交试验年度报告并接受年度跟踪审查及再备案。

4、该研究进行过程中将接受伦理委员会的持续审查，伦理委员会有权根据实际进展情况改变持续审查频率。

5、批件超过有效期请提前 1 个月提出申请。

主任委员签字：[signature]

（伦理委员会盖章）

郑州大学第一附属医院 科研和临床试验伦理委员会

2018 年 10 月 25 日

| | |
|---|---|
| 年度/定期跟踪审查频率：□3 个月 □6 个月 ■12 个月 | 此批件有效期至：2019 年 10 月 25 日 |

**声明：**

1.本院伦理委员会职责、人员组成，操作规程及记录均遵循 ICH-GCP、CFDA-GCP、中国相关法规。

2.“同意”的研究应遵循已经伦理委员会批准的方案执行，应符合 ICH-GCP、CFDA-GCP 和《赫尔辛基宣言》的原则。

3.“作必要的修正后同意”的研究请按评审意见进行逐条修改并递交临床研究文件修正申请及新版本文件，申请经伦理委员会同意后，方可获得批准编号。

4.“作必要的修正后重审”的研究请按评审意见进行修改，并递交临床研究文件修正申请及新版本文件，在下次全体会议中审查，由全体委员投票决定结果。

5.“不同意”或“暂停或终止已经批准的临床试验”的研究方案，申办者和研究者有权就伦理委员会的意见和建议中提及的问题做书面申诉，由全体委员投票决定结果。

6.本中心发生的严重不良事件或意外不良事件需在向 CFDA 上报的同时向伦理委员会作书面报告，必要时会邀请研究者参加全体会议进行审查，伦理委员会有权根据对其的评估并做出新的决定。

7.无论试验开始与否，请在下次持续审查已到期前 1 个月提出持续审查的申请。

8.郑州大学第一附属医院科研和临床试验伦理委员会

地址：郑州市建设东路 1 号郑州大学第一附属医院，邮编：450052

秘书处联系人：田丽　　联系电话：0371-66295219

E-mail: tianli_llzl@163.com
