# Supplementary material for: Endovascular treatment of acute ischemic stroke with a fully radiopaque retriever: A randomized controlled trial
Source: Front Neurol. 2022 Dec 14;13:962987. doi: 10.3389/fneur.2022.962987 (PMC9796564; doi:10.3389/fneur.2022.962987)
Supplement: Supplementary file 1 [file Data_Sheet_1.zip › 03 │ú╓▌╩╨╥╗.pdf]

常州市第一人民医院伦理委员会

伦理委员会审查意见

2017（器）CL008-01

会议地点	常州市第一人民医院 5 号楼 5 楼会议室	会议日期	2017.08.10
审查类别	初始审查	审查方式	会议审查
试验项目	取栓器治疗急性缺血性卒中的前瞻性、多中心、单盲、随机对照临床试验		
器械类别	III类		
临床试验总例数	320	我院承担例数	30
申 办 单 位	微创神通医疗科技（上海）有限公司	主要负责人	刘慧萍
组长单位	上海长海医院	主要研究者	刘建民
临床研究单位	常州市第一人民医院	临床研究部门	神经外科
主要研究者	彭亚	职 称	主任医师

	需 提 供 审 查 文 件 名 称	版 本	日 期
✓	初始伦理审查申请书		2017.07.04
✓	研究经济利益声明		2017.05.16
✓	临床试验方案	V1.0	2017.03.08
✓	知情同意书	V1.0	2017.03.08
✓	受试者招募说明		2017.03.10
✓	原始病历和病例报告表	V1.0	2017.03.08
✓	研究者手册	V1.0	2017.03.08
✓	医疗器械说明书		
✓	医疗器械产品技术要求		
✓	医疗器械检验报告		
✓	医疗器械动物实验报告		
✓	研究者履历表和主要研究者专业简历		
✓	组长单位伦理委员会批准函		2017.04.24
✓	申办方资质证明		
✓	CRO 资质证明和委托书		
✓	研究中心满足条件的综述和申办方关于医疗器械质量的声明		
✓	保险		

第一人民医院 伦理委员会

审查意见：

根据卫生部《涉及人的生物医学研究伦理审查办法》（2016）、SFDA《药物临床试验质量管理规范（2003）》、《医疗器械临床试验质量管理规范（2016）》、WMA《赫尔辛基宣言》和CIOMS《人体生物医学研究国际道德指南》的伦理原则，经本伦理委员会审查，意见如下：

1. 在知情同意书第 1 页“试验背景”中补充机械取栓的现状与不足。
2. 修改完善知情同意书中“试验过程-术中”里“手术情况”，具体写明评价指标等内容。
3. 在知情同意书中补充明确如何确定取栓支架是否正确使用。
4. 修改知情同意书第 3 页第六项第二段，去掉“直接”，并明确材料费等其他相关费用也由申办方支付。

5.删除知情同意书第 4 页第九项“与试验相关伤害的治疗和经济补偿”中“经医疗鉴定的与试验支架有关的直接伤害”中“直接”二字，在此项中增加造成人身损害的相关赔偿。

6. 在知情同意书第 5 页第十三项中明确再次发生急性缺血性卒中时，再次取栓时所用支架应为 solitaire 支架而非试验用支架。

按审查意见修改后的文件，或对审查意见不同观点的陈诉，请提交“复审申请”，方案/知情同意书请注明新的版本号和版本日期，并以阴影和/或下划线方式标注修改部分，报伦理委员会审查，经批准后执行。

主任委员（签名）：

常州市第一人民医院伦理委员会（盖章）

2017 年 08 月 16 日

常州市第一人民医院伦理委员会

会议签到表

伦理委员会名称	常州市第一人民医院伦理委员会
会议日期	2017 年 08 月 10 日

伦理委员会职务	姓名	性别	职务、职称	专业	签名
主任委员	张晓膺	男	党委书记、主任医师	临床医学	[illegible]
副主任委员	许国锋	男	副院长、主任医师	临床医学	[illegible]
委员	华　飞	男	副院长、主任医师	临床医学	[illegible]
委员	倪静玉	女	护理部主任、主任护师	护理	[illegible]
委员	周　健	女	物资采购中心主任、主任护师	护理	[illegible]
委员	王莉英	女	药剂科副主任、主任中药师	药学	[illegible]
委员	徐天舒	男	颌面外科主任、主任医师	口腔专业	[illegible]
委员	倪慧萍	女	医务处副主任、主任医师	临床医学	[illegible]
委员	练学淦	男	科教科副科长、副主任医师	临床医学	[illegible]
委员	陆　皓	男	泌尿外科副主任、副主任医师	临床医学	[illegible]
委员	王　亮	男	法律顾问、合伙律师	法律	[illegible]
委员	吴玉娣	女	天宁街道人大办公室主任助理	社区工作	[illegible]
委员、秘书	王梦洁	女	科室负责人、助理政工师	医院管理	/

常州市第一人民医院伦理委员会

伦理委员会审查批件

（2017）器第 10 号

会议地点	常州市第一人民医院5号楼5楼会议室	会议日期	2017.08.10
审查类别	初始审查	审查方式	会议审查
试验项目	取栓器治疗急性缺血性卒中的前瞻性、多中心、单盲、随机对照临床试验		
器械类别	III类		
临床试验总例数	320	我院承担例数	30
申 办 单 位	微创神通医疗科技（上海）有限公司	主要负责人	刘慧萍
组长单位	上海长海医院	主要研究者	刘建民
临床研究单位	常州市第一人民医院	临床研究部门	神经外科
主要研究者	彭亚	职　称	主任医师

需 提 供 审 查 文 件 名 称		版　本	日　期
√	初始伦理审查申请书		2017.07.04
√	研究经济利益声明		2017.05.16
√	临床试验方案	V1.0	2017.03.08
√	知情同意书	V2.0	2017.08.24
√	受试者招募说明		2017.03.10
√	原始病历和病例报告表	V1.0	2017.03.08
√	研究者手册	V1.0	2017.03.08
√	医疗器械说明书		
√	医疗器械产品技术要求		
√	医疗器械检验报告		
√	医疗器械动物实验报告		
√	研究者履历表和主要研究者专业简历		
√	组长单位伦理委员会批准函		2017.04.24
√	申办方资质证明		
√	CRO 资质证明和委托书		
√	研究中心满足条件的综述和申办方关于医疗器械质量的声明		
√	保险		
√	复审申请		2017.09.05

（2017）器第 10 号

审查决定：

1. 根据卫生部《涉及人的生物医学研究伦理审查办法》（2016）、SFDA《药物临床试验质量管理规范（2003）》、《医疗器械临床试验质量管理规范（2016）》、WMA《赫尔辛基宣言》和 CIOMS《人体生物医学研究国际道德指南》的伦理原则，本伦理委员会进行认真的讨论并进行了投票表决。结论：作必要的修正后同意。

2. 2017 年 09 月 06 日收到申办方递交的知情同意书（版本号：V2.0 版本日期：2017.08.24）等材料，经主审委员和主任委员审核，认为已符合伦理委员会 2017 年 08 月 10 日会议的要求。

结论：同意按所批准的临床研究方案、知情同意书开展本项研究。

3. 临床试验中若修改方案、知情同意书和招募材料要重新报伦理委员会审查批准后执行；发生严重不良事件以及影响研究风险与受益比的非预期不良事件应及时报告；申办者或研究者暂停 / 终止研究，申请人应通知伦理委员会并报告暂停 / 终止的原因；暂停 / 终止的研究所取得结果的总结应递交伦理委员会；研究完成后提交结题或总结报告；

4. 年度/定期跟踪审查频率为：12 个月/次，请按此频率在截止日期前 1 个月提交年度/定期跟踪审查报告。

5. 批件的有效期为：1 年。

主任委员（签名）：[illegible]

常州市第一人民医院伦理委员会（盖章）

2017 年 9 月 8 日

伦理委员会联系热线： 051968870201

申请负责人手机： 13093178444

传 真：051986621235

固定电话：051968871283

常州市第一人民医院伦理委员会

伦理委员会审查意见

2017（器）CL008-02

会议地点	常州市第一人民医院5号楼5楼会议室	会议日期	2018.11.13
审查类别	跟踪审查	审查方式	会议审查
试验项目	取栓器治疗急性缺血性卒中的前瞻性、多中心、单盲、随机对照临床试验		
药物/器械类别	III类		
临床试验总例数	238	我院承担例数	30
申 办 单 位	微创神通医疗科技（上海）有限公司	主要负责人	刘慧萍
组长单位	上海长海医院	主要研究者	刘建民
临床研究单位	常州市第一人民医院	临床研究部门	神经外科
主要研究者	彭亚	职 称	主任医师

需提供审查文件名称		版 本	日 期
✓	修正方案审查申请		2018.10.16
✓	组长单位伦理委员会审查批件		2018.09.25
✓	试验方案（纯净版与痕迹版）	V2.0	2018.08.08
✓	试验方案修订说明及修订列表		
✓	研究者手册（纯净版与痕迹版）	V2.0	2018.08.08
✓	研究者手册修订列表		
✓	知情同意书（纯净版与痕迹版）	V3.0	2018.09.09
✓	知情同意书修订列表		
✓	病例报告表（纯净版与痕迹版）	V3.0	2018.08.08
✓	病例报告表修订列表		
✓	原始病历（纯净版与痕迹版）	V3.0	2018.08.08
✓	原始病历修订列表		
✓	取栓器使用说明书（纯净版与痕迹版）		
✓	取栓器使用说明书修订列表		

审查意见：

根据卫生部《涉及人的生物医学研究伦理审查办法》（2016）、SFDA《药物临床试验质量管理规范（2003）》、《医疗器械临床试验质量管理规范（2016）》、WMA《赫尔辛基宣言》和CIOMS《人体生物医学研究国际道德指南》的伦理原则，经本伦理委员会审查，意见如下：

该研究方案、知情同意书等修改符合伦理要求，批准在我院继续进行研究。

有效期：批准之日起至2019年09月06日。

主任委员（签名）：[signature]

常州市第一人民医院伦理委员会（盖章）

2018年11月15日
