# Supplementary material for: Endovascular treatment of acute ischemic stroke with a fully radiopaque retriever: A randomized controlled trial
Source: Front Neurol. 2022 Dec 14;13:962987. doi: 10.3389/fneur.2022.962987 (PMC9796564; doi:10.3389/fneur.2022.962987)
Supplement: Supplementary file 1 [file Data_Sheet_1.zip › 04 ╞δ┬│╥╜╘║.pdf]

# 伦理审查意见

| | | | |
|---|---|---|---|
| 项目编号 | 2017022 | | |
| 项目名称 | 取栓器治疗急性缺血性卒中的前瞻性、多中心、单盲、随机对照临床试验 | | |
| 申办者 | 微创神通医疗科技（上海）有限公司 | 主要研究者 | 吴伟 |
| 审查日期 | 20170607 | | |

审查意见：

一、试验方案

1、研究者手册、方案、知情同意书中均提到了参加研究中心共 15 家，但两处专列的名单中为 11 家。请进行统一。

2、申办方在手册中选到了 43 篇今年的国外文献列出研究重点，伦理审查重视受试者的安全性，其中 21 篇注明死亡率为 5.7%-45%，症状性颅内出血 11 篇中为 0-17%，若计算平均数应为 20.3%和 7.4%。病人死亡率为何相差巨大？

3、方案中排除标准共 27 项，尚不能完全包括，建议增加第 28 项，“研究者认为其他不宜入选者”

4、研究者手册、方案和知情同意书列出了风险共 34 项等，不知哪些是常见、比较常见、和罕见，能否明确。

5、若受试者发生心梗，其处理费用谁来承担？

二、知情同意书

1、第 6 条第二段及第 9 条需修改，申办方不能仅承担治疗费用，还要承担相应的损害赔偿。将第 9 条中的“直接损害”中“直接”删除，应包括间接损害。

2、第 6 页第 13 行，“副本”二字删除，应当为原件。

3、本项目要求病人在 6 小时内治疗，知情同意书是否可以适当简化？

4、第一部分应告知受试者，本研究经山东大学齐鲁医院药物临床试验伦理委员会审核通过。

5、受试者随访几次？

6、第 5 页第 3 行“但不包括术中可能使用的其他器械”指的是什么？应当是为受试者免费提供的。是否应当表述清楚。

7、请删去第 6 页 16 行中的未成年人字样，因为受试者均大于 18 岁。

**审查决定：** 1、☐同意；　2、■作必要的修正后同意　3、☐作必要的修正后重审　4、☐终止或暂停已批准的试验　5、☐不同意；

| | |
|---|---|
| 年度/定期跟踪审查频率 | 12 个月 |
| 如需调整年度/定期跟踪审查频率，调整后的年度/定期跟踪审查频率 | |
| 主任委员签名及日期 | [illegible] 2017.6.7 |

山东大学齐鲁医院 国家药品临床试验伦理委员会

# 山东大学齐鲁医院药物临床试验伦理委员会

## 临床试验伦理审查批件（医疗器械） 山东大学齐鲁医院

批件号：2017022

| 项目名称 | 取栓器治疗急性缺血性卒中的前瞻性、多中心、单盲、随机对照临床试验 | 项目编号 | 2017022 |
|---|---|---|---|
| 研究目的 | 通过和 Solitaire FR（ev3/Medtronic）治疗急性缺血性卒中的有效性和安全性比较，评价试验取栓器的有效性和安全性 | 规格型号 | AIS4025、AIS6030 |
| 适应症 | 1）适用于急性缺血性卒中症状出现 6 小时内，静脉用组织性纤溶酶原激活剂（IV t-PA）治疗失败或者禁忌的前循环血管栓塞的患者血流开通。<br>2）靶病变血管直径 2.0～5.0 mm。 | | |
| 主要研究者专业科室 | 神经内科 | 项目负责人 | 吴伟 |
| 申 办 者 | 微创神通医疗科技（上海）有限公司 | | |

**本伦理委员会是相对独立的，委员会组成和工作程序符合临床试验管理规范要求及相关法律法规。已审阅并批准下面划[✓]的研究相关文件：**

[✓] CFDA 医疗器械质量监督检验中心检验报告：国医检（械）字 ZC2016 第 636 号、第 637 号
[✓] 临床试验委托书
[✓] 研究方案： 版本号 V1.0 版本日期：2017-03-08
[✓] 知情同意书： 版本号 V2.0 版本日期：2017-06-15
[✓] 医疗器械说明书
[✓] 研究者手册 ：版本号 V1.0 版本日期：2017-03-08
[✓] 病例报告表：版本号 V1.0 版本日期：2017-03-08
[✓] 产品的自测报告
[✓] 申办者资质
[✓] 研究者简历及 GCP 证书
[✓] 试验用医疗器械的研制符合适用的医疗器械质量管理体系相关要求承诺书
[✓] 原始病历：版本号 V1.0 版本日期：2017-03-08
[✓] 临床试验保险
[✓] 招募广告说明
[✓] CRO 资质
[✓] GLP 实验室对微创神通取栓器在猪动物模型的治疗评估 总结报告（研究编号：FRR1602W，发布日期：2017 年 1 月 9 日）CN
GLP Evaluation Of MicroPort Neuro Tech Stentriever Thrombectomy System in a Swine Model Final Report （Study Number：FRR1602W，Issue Date：January9，2017）EN

同意开展本临床试验，本批件自批准之日起 1 年内有效。

跟踪审查频率为： ☐三个月 ☐六个月 ☒十二个月

研究过程中若变更主要研究者，对临床研究方案、知情同意书、招募材料等的任何修改，请申请人提交修正案审查申请。发生严重不良事件，请申请人及时提交严重不良事件报告。请按照伦理委员会规定的年度/定期跟踪审查频率，申请人在截止日期前 1 个月提交研究进展报告。出现违背/偏离方案的情况，请提交违背/偏离方案报告。申请人暂停或提前终止研究，请及时提交暂停/终止研究报告。完成临床研究，请申请人提交结题报告。本临床试验的生物标本仅用于方案规定的数据监测，禁止用于任何其他目的。否则将追究所有相关责任人法律责任。

主任委员签名：[signature]

会议审议日期 2017 年 6 月 7 日 批准日期 2017 年 7 月 9 日

国家药物临床试验伦理委员会

伦理委员会地址：中国济南市文化西路 107 号山东大学齐鲁医院和平楼 250012

# 山东大学齐鲁医院药物临床试验伦理委员会

## 临床试验伦理审查批件（医疗器械）

山东大学 齐鲁医院

批件号：2017022(1)

| | | | |
|---|---|---|---|
| 项目名称 | 取栓器治疗急性缺血性卒中的前瞻性、多中心、单盲、随机对照临床试验 | 项目编号 | 2017022 |
| 研究目的 | 通过和 Solitaire FR（ev3/Medtronic）治疗急性缺血性卒中的有效性和安全性比较，评价试验取栓器的有效性和安全性。 | 规格型号 | AIS4025、AIS6030 |
| 适应症 | 适用于大血管闭塞导致的急性缺血性脑卒中 | | |
| 主要研究者专业科室 | 神经内科 | 项目负责人 | 吴伟 |
| 申 办 者 | 微创神通医疗科技（上海）有限公司 | | |

本伦理委员会是相对独立的，委员会组成和工作程序符合临床试验管理规范要求及相关法律法规。已审阅并批准下面划[✓]的研究相关文件：

[✓]修正案审查申请

[✓]组长单位批件

[✓]研究方案：版本号 V2.0，版本日期 2018-08-08

[✓]知情同意书：版本号 V3.0 ，版本日期 2018-09-09

[✓]研究者手册：版本号 V2.0，版本日期 2018-08-08

[✓]病例报告表：版本号：V3.0,版本日期 2018-08-08

[✓]原始病历：版本号 V3.0，版本日期 2018-08-08

[✓]取栓器说明书：版本号 V2.0

本批件自批准之日起 1 年内有效。

跟踪审查频率为：　☐三个月　☐六个月　☒十二个月

研究过程中若变更主要研究者，对临床研究方案、知情同意书、招募材料等的任何修改，请申请人提交修正案审查申请。发生严重不良事件，请申请人及时提交严重不良事件报告。请按照伦理委员会规定的年度/定期跟踪审查频率，申请人在截止日期前 1 个月提交研究进展报告。出现违背/偏离方案的情况，请提交违背/偏离方案报告。申请人暂停或提前终止研究，请及时提交暂停/终止研究报告。完成临床研究，请申请人提交结题报告。本临床试验的生物标本仅用于方案规定的数据监测，禁止用于任何其他目的。否则将追究所有相关责任人法律责任。

主任委员签名：

审议日期：2018年10月20日　　批准日期：2018年10月20日

伦理委员会地址：中国济南市文化西路 107 号山东大学齐鲁医院和平楼　250012
