# Supplementary material for: Endovascular treatment of acute ischemic stroke with a fully radiopaque retriever: A randomized controlled trial
Source: Front Neurol. 2022 Dec 14;13:962987. doi: 10.3389/fneur.2022.962987 (PMC9796564; doi:10.3389/fneur.2022.962987)
Supplement: Supplementary file 1 [file Data_Sheet_1.zip › 06 ╢1⁄2╖╜.pdf]

上海市东方医院药物临床试验伦理委员会批件

声明：本伦理委员会按照中国 GCP、ICH-GCP 和有关法规组成和工作，其审查工作过程不受伦理委员会以外任何组织及个人影响

批件号：【2017】临审第(011)号修正 1

项目名称	取栓器治疗急性缺血性卒中的前瞻性、多中心、单盲、随机对照临床试验					
类别	医疗器械	注册分类	三类	试验目的	临床验证	
专业名称	神经内科		试验项目负责人	李 刚	职称	主任医师
			专业负责人	李 刚	职称	主任医师
审评材料	1. 研究方案（V2.0/2018 年 8 月 8 日）<br>2. 知情同意书（V2.0/2018 年 8 月 8 日）<br>3. 研究者手册（V2.0/2018 年 8 月 8 日）<br>4. 病例报告表（V3.0/2018 年 8 月 8 日）<br>5. 原始病历（V3.0/2018 年 8 月 8 日）<br>6. 取栓器说明书 Rev.2.0			主要参加单位	上海长海医院等	
审查方式：	■会议审查 □快速审查					
投票结果	会议时间：2018 年 10 月 31 日，应到人数 11 人，实到人数 11 人；其中：投票人数 10 人，回避 1 人（李刚）					
	会议地点：东方医院南院 5 楼第二会议室					
	其中：同意 10 票；作必要的修正后同意 0 票；作必要的修正后重审 0 票；不同意 0 票；暂停或终止已批准的试验 0 票					
审评意见	结论：■同意 □作必要修改后同意 □作必要的修正后重审 □不同意 □暂停或终止已批准的试验					
	是否需要持续审查？ ■是 □否		持续审查频率：□3 个月 □6 个月 ■12 个月			
	审评意见：<br>递交的研究方案、知情同意书等材料（详见审评材料），符合伦理规范及相关法律法规，我院伦理委员会同意并接受上述材料备案，对项目资料的更新请及时递交。<br>主任委员（签章）医学伦理委员会<br>日期：2018 年 11 月 7 日					

伦理委员会地址：上海市浦东新区即墨路 150 号 邮政编码：200120
上海市浦东新区云台路 1800 号 邮政编码：200123
伦理委员会联系电话：021-38804518-22198

上海市东方医院药物临床试验伦理委员会名单

姓名	性别	职称	伦理委员会任职	单位	专业	签名
雷　撼	男	主任医师	主任委员	上海市东方医院	呼吸内科	[signature]
范列英	女	主任医师	委员	上海市东方医院	检验科	[signature]
何志高	男	主任药师	委员	上海市东方医院	医院药学	[signature]
江　华	女	主任医师	委员	上海市东方医院	老年医学科	[signature]
王学斌	女	主任医师	委员	上海市东方医院	ICU	[signature]
翟晓波	男	主任药师	委员	上海市东方医院	药理学	[signature]
李　刚	男	主任医师	委员	上海市东方医院	神经内科	[signature]
钟　岚	女	主任医师	委员	上海市东方医院	消化内科	[signature]
张震方	男	律师	委员	上海市长江律师事务所	法律	[signature]
朱兰芳	女	助理工程师	委员	上海市浦东新区陆家嘴街道上港居委会	社会	[signature]
鲍思蔚	女	副主任药师	委员/秘书	上海市东方医院	药学	[signature]

（印章：上海市东方医院（同济大学附属东方医院）医学伦理委员会）

备注：

1、本批件可能在其他中心机构及其伦理审查委员会备案。如对研究在贵机构实施的可行性（包括研究者的资格与经验、设备与条件等）有不同意见，请及时与本伦理审查委员会联系。

2、已批准项目须遵循本伦理审查委员会批准的方案执行，须符合 CFDA/GCP 和《赫尔辛基宣言》的原则。

3、暂停/提前终止临床研究，请及时通知本伦理审查委员会。

4、本中心发生的严重不良事件及影响研究风险收益比的非预期事件，须及时报告本伦理审查委员会。

5、研究过程中，对研究方案和知情同意书等相关文件所作的任何修订，均需得到伦理委员会审查同意后方可实施。

6、发现违反方案情况须及时汇报。

7、根据伦理审查委员会对持续审查频度的意见，无论试验开始与否，请在持续审查日到期前 1 个月提出持续审查的申请。

8、完成临床研究，须提交结题报告供伦理审查委员会审查。

9、本批件的有效期为 1 年，逾期未实施的则自动废止。

上海市东方医院药物临床试验伦理委员会批件

声明：本伦理委员会按照中国 GCP、ICH-GCP 和有关法规组成和工作，其审查工作过程不受伦理委员会以外任何组织及个人影响

批件号：【2017】临审第(011)号修正 1

<table>
<tr><td>项目名称</td><td colspan="7">取栓器治疗急性缺血性卒中的前瞻性、多中心、单盲、随机对照临床试验</td></tr>
<tr><td>类别</td><td>医疗器械</td><td>注册分类</td><td>三类</td><td>试验目的</td><td colspan="3">临床验证</td></tr>
<tr><td rowspan="2">专业名称</td><td colspan="2" rowspan="2">神经内科</td><td>试验项目负责人</td><td>李　刚</td><td>职称</td><td colspan="2">主任医师</td></tr>
<tr><td>专业负责人</td><td>李　刚</td><td>职称</td><td colspan="2">主任医师</td></tr>
<tr><td>审评材料</td><td colspan="3">1. 研究方案（V2.0/2018 年 8 月 8 日）
2. 知情同意书（V2.0/2018 年 8 月 8 日）
3. 研究者手册（V2.0/2018 年 8 月 8 日）
4. 病例报告表（V3.0/2018 年 8 月 8 日）
5. 原始病历（V3.0/2018 年 8 月 8 日）
6. 取栓器说明书 Rev.2.0</td><td>主要参加单位</td><td colspan="3">上海长海医院等</td></tr>
<tr><td colspan="8">审查方式：　■会议审查　□快速审查</td></tr>
<tr><td rowspan="3">投票结果</td><td colspan="7">会议时间：2018 年 10 月 31 日，应到人数 11 人，实到人数 11 人；其中：投票人数 10 人，回避 1 人（李刚）</td></tr>
<tr><td colspan="7">会议地点：东方医院南院 5 楼第二会议室</td></tr>
<tr><td colspan="7">其中：同意 10 票；作必要的修正后同意 0 票；作必要的修正后重审 0 票；不同意 0 票；
暂停或终止已批准的试验 0 票</td></tr>
<tr><td rowspan="3">审评意见</td><td colspan="7">结论：■同意　□作必要修改后同意　□作必要的修正后重审　□不同意
□暂停或终止已批准的试验</td></tr>
<tr><td colspan="3">是否需要持续审查？　■是　□否</td><td colspan="4">持续审查频率：□3 个月　□6 个月　■12 个月</td></tr>
<tr><td colspan="7">审评意见：

递交的研究方案、知情同意书等材料（详见审评材料），符合伦理规范及相关法律法规，我院伦理委员会同意并接受上述材料备案，对项目资料的更新请及时递交。

上海市东方医院(同济大学附属东方医院)医学伦理委员会
主任委员（签章）
日期：2018 年 11 月 7 日</td></tr>
</table>

伦理委员会地址：上海市浦东新区即墨路 150 号　　邮政编码：200120
上海市浦东新区云台路 1800 号　　邮政编码：200123
伦理委员会联系电话：021-38804518-22198
