# Supplementary material for: Endovascular treatment of acute ischemic stroke with a fully radiopaque retriever: A randomized controlled trial
Source: Front Neurol. 2022 Dec 14;13:962987. doi: 10.3389/fneur.2022.962987 (PMC9796564; doi:10.3389/fneur.2022.962987)
Supplement: Supplementary file 1 [file Data_Sheet_1.zip › 07 ║■▒▒╚2╘║.pdf]

湖北省第三人民医院药物临床试验伦理委员会

Ethics Committee of The Third People's Hospital of HuBei of Drug Clinical Trial

伦理审查批件

Ethics Review Approval

<table>
<tr><td>批件号</td><td colspan="6">HBSDSRMYY 2017-A004-01</td></tr>
<tr><td>项目名称</td><td colspan="6">取栓器治疗急性缺血性卒中的前瞻性、多中心、单盲、随机对照临床研究</td></tr>
<tr><td>申办者</td><td colspan="6">微创神通医疗科技(上海)有限公司</td></tr>
<tr><td>研究单位</td><td colspan="6">湖北省第三人民医院神经内科</td></tr>
<tr><td>主要研究者</td><td colspan="6">彭小祥</td></tr>
<tr><td>审查类别</td><td colspan="2">初始审查</td><td colspan="2">审查方式</td><td colspan="2">会议审查</td></tr>
<tr><td>审查日期</td><td colspan="2">2017 年 9 月 28 日</td><td colspan="2">审查地点</td><td colspan="2">诚信楼 13 楼会议室</td></tr>
<tr><td>审查委员</td><td colspan="6">邓小容、李先林、罗金波、连莉、王云霞、毕俊英、刘世国、夏莉娅、陈桢艳、李红华、张三江、李龙</td></tr>
<tr><td>批准文件</td><td colspan="6">1. 研究方案：V1.0
2. 知情同意书：V1.0
3. 受试者招募说明：V1.0
4. 病例报告表：V1.0
5. 研究者手册：V1.0
6. 原始病历：V1.0</td></tr>
<tr><td rowspan="3">投票结果</td><td colspan="6">本次会议应到人数 13；实到人数 13；投票人数 12；回避人数 1 人</td></tr>
<tr><td>同 意</td><td>作必要的修正后同意</td><td>作必要的修正后重审</td><td>不同意</td><td>终止或暂停试验</td><td>弃 权</td></tr>
<tr><td>12 票</td><td>0 票</td><td>0 票</td><td>0 票</td><td>0 票</td><td>0 票</td></tr>
<tr><td>审查结论</td><td>■同意</td><td>□做必要修正后同意</td><td>□做必要修正后重审</td><td>□不同意</td><td colspan="2">□终止或暂停试验</td></tr>
<tr><td colspan="7">根据卫生部《涉及人的生物医学研究伦理审查办法（试行）》（2007）、SFDA《药物临床试验质量管理规范（2003）》、《医疗器械临床试验规定（2004）》、WMA《赫尔辛基宣言》和 CIOMS《人体生物医学研究国际道德指南》的伦理原则，经本伦理委员会审查，免除知情同意书审查，同意所批准的临床研究方案开展研究。
请遵循 GCP 原则、遵循伦理委员会批准的方案开展临床研究，保护受试者的健康与权利。研究开始前，请申请人完成临床试验注册。
研究过程中若变更主要研究者，对临床研究方案、知情同意书、招募材料等的任何修改，请申请人提交修正案审查申请。发生严重不良事件，请申请人及时提交严重不良事件报告。</td></tr>
</table>

湖北省第三人民医院 药物临床试验伦理委员会

请按照伦理委员会规定的年度/定期跟踪审查频率，申请人在截止日期前 1 个月提交研究进展报告。<br>出现没有遵从方案开展研究的情况；或可能对受试者的权益/健康、以及研究的科学性造成不良影响等违背 GCP 原则的情况，请申办者/监查员/研究者提交违背方案报告。<br>申请人暂停或提前终止临床研究，请及时提交暂停/终止研究报告。<br>完成临床研究，请申请人提交结题报告。	
跟踪审查频率	一年/一次
有效期	一年
联系人与联系电话	陈桢艳 027-83745616
主任/副主任委员签字	李光林
湖北省第三人民医院药物临床试验伦理委员会（盖章）	湖北省第三人民医院 药物临床试验 伦理委员会
日期：2017 年 9 月 28 日	
声明：本伦理委员会的职责、人员组成、操作程序及记录遵循中华人民共和国食品药品监督管理局颁布的药物临床试验质量管理规范（GCP）和 ICH-GCP 的伦理审查原则，并遵守中国的相关法律及法规。<br>伦理地址：湖北省武汉市硚口区中山大道 26 号，邮编 430033；	

附录

取栓器治疗急性缺血性卒中的前瞻性、多中心、单盲、随机对照临床试验(2016-GATOR-01-A)

伦理初审文件目录

1、初始审查申请

2、临床研究方案 V1.0 2017-03-08

3、知情同意书 V1.0 2017-03-08

4、受试者招募说明 2017-03-10

5、病例报告表 V1.0 2017-03-08

6、研究者手册 V1.0 2017-03-08

7、原始病历 V1.0 2017-03-08

8、说明书

9、医疗器械产品复核通过注册产品标准或相应的国家、行业标准

10、产品检验报告：国医检（械）字 ZC2016 第 636 号（AIS4025)、国医检（械）字 ZC2016 第 637 号（AIS6030）

11、自测报告

12、动物实验报告

13、主要研究者简历

14、组长单位批件

15、企业资质（营业执照、营业执照副本、组织机构代码证、税务登记证））、CRO 资质（营业执照副本）、CRO 授权书、CRA 授权书

16、保险 单号：92666573

湖北省第三人民医院药物临床试验伦理委员会

Ethics Committee of The Third People's Hospital of HuBei of Drug Clinical Trial

会议签到表

Sign-in Sheet of Meeting

项目名称	取栓器治疗急性缺血性卒中的前瞻性、多中心、单盲、随机对照临床研究
会议日期	2017 年 9 月 28 日
会议地点	行政楼 3 楼会议室

姓名	性别	工作单位	伦理委员会职务	职称	出席者签名
彭小祥	男	湖北省第三人民医院	主任委员	主任医师	彭小祥
邓小容	女	湖北省第三人民医院	副主任委员	主任医师	邓小容
李先林	男	湖北省第三人民医院	副主任委员	主任医师	李先林
罗金波	男	湖北省第三人民医院	委员	主任医师	罗金波
皮勇	男	湖北省第三人民医院	委员	副主任医师	
廖亚玲	女	湖北省第三人民医院	委员	副主任药师	
连利	女	湖北省第三人民医院	委员	副主任医师	连利
王云霞	女	湖北省第三人民医院	委员	主任医师	王云霞
毕俊英	男	湖北省第三人民医院	委员	副主任医师	毕俊英
池卫明	男	湖北省第三人民医院	委员	副主任药师	
李勇光	男	湖北省第三人民医院	委员	副主任医师	
刘世国	男	湖北省第三人民医院	委员	主任技师	刘世国
林琍	女	湖北省第三人民医院	委员	主任医师	
夏莉娅	女	湖北省第三人民医院	委员	副主任护师	夏莉娅
陈桢艳	女	湖北省第三人民医院	委员	主治医师	陈桢艳
李红华	男	湖北鑫卫律师所	委员	律师	李红华
张二江	男	湖北中烟医务部	委员	主治医师	张二江
李龙	男	[illegible]军工社区	委员	居委会副书记	李龙

委员到会情况	应到人数：	实到人数：

湖北省第三人民医院 药物临床试验伦理委员会

湖北省第三人民医院药物临床试验伦理委员会

Ethics Committee of The Third People's Hospital of HuBei of Drug Clinical Trial

伦理审查批件

Ethics Review Approval

<table>
<tr><td>批件号</td><td colspan="6">HBSDSRMYY 2017-A004-02</td></tr>
<tr><td>项目名称</td><td colspan="6">取栓器治疗急性缺血性卒中的前瞻性、多中心、单盲、随机对照临床研究</td></tr>
<tr><td>申办者</td><td colspan="6">微创神通医疗科技(上海)有限公司</td></tr>
<tr><td>研究单位</td><td colspan="6">湖北省第三人民医院神经内科</td></tr>
<tr><td>主要研究者</td><td colspan="6">彭小祥</td></tr>
<tr><td>审查类别</td><td colspan="2">修正案审查</td><td colspan="2">审查方式</td><td colspan="2">快速审查</td></tr>
<tr><td>审查日期</td><td colspan="2">2018 年 10 月 24 日</td><td colspan="2">审查地点</td><td colspan="2">行政楼 3 楼会议室</td></tr>
<tr><td>审查委员</td><td colspan="6">夏莉娅、李勇光</td></tr>
<tr><td>批准文件</td><td colspan="6">1. 研究方案：V2.0
2. 知情同意书：V2.0
3. 研究者手册：V2.0
4. 病例报告表：V3.0
5. 原始病例：V3.0
6. 取栓器说明书：V2.0</td></tr>
<tr><td rowspan="3">投票结果</td><td colspan="6">本次会议应到人数 2；实到人数 2；投票人数 2；回避人数 0 人</td></tr>
<tr><td>同 意</td><td>作必要的修正后同意</td><td>作必要的修正后重审</td><td>不同意</td><td>终止或暂停试验</td><td>弃 权</td></tr>
<tr><td>2 票</td><td>0 票</td><td>0 票</td><td>0 票</td><td>0 票</td><td>0 票</td></tr>
<tr><td>审查结论</td><td>■同意</td><td>□做必要修正后同意</td><td>□做必要修正后重审</td><td>□不同意</td><td colspan="2">□终止或暂停试验</td></tr>
</table>

根据卫生部《涉及人的生物医学研究伦理审查办法（试行）》（2007）、SFDA《药物临床试验质量管理规范（2003）》、《医疗器械临床试验规定（2004）》、WMA《赫尔辛基宣言》和 CIOMS《人体生物医学研究国际道德指南》的伦理原则，经本伦理委员会审查，免除知情同意书审查，同意所批准的临床研究方案开展研究。

请遵循 GCP 原则、遵循伦理委员会批准的方案开展临床研究，保护受试者的健康与权利。研究开始前，请申请人完成临床试验注册。

研究过程中若变更主要研究者，对临床研究方案、知情同意书、招募材料等的任何修改，请申请人提交修正案审查申请。发生严重不良事件，请申请人及时提交严重不良事件报告。

请按照伦理委员会规定的年度/定期跟踪审查频率，申请人在截止日期前 1 个月提交研究进展报告。

出现没有遵从方案开展研究的情况；或可能对受试者的权益/健康、以及研究

的科学性造成不良影响等违背 GCP 原则的情况，请申办者/监查员/研究者提交违背方案报告。

申请人暂停或提前终止临床研究，请及时提交暂停/终止研究报告。

完成临床研究，请申请人提交结题报告。

跟踪审查频率	一年/一次
有效期	一年
联系人与联系电话	陈桢艳 027-83745616
主任/副主任委员签字	[illegible]
湖北省第三人民医院药物临床试验伦理委员会（盖章）	
日期：2018年10月24日	

声明：本伦理委员会的职责、人员组成、操作程序及记录遵循中华人民共和国食品药品监督管理局颁布的药物临床试验质量管理规范（GCP）和 ICH-GCP 的伦理审查原则，并遵守中国的相关法律及法规。

伦理地址：湖北省武汉市硚口区中山大道 26 号，邮编 430033；
