# Supplementary material for: Endovascular treatment of acute ischemic stroke with a fully radiopaque retriever: A randomized controlled trial
Source: Front Neurol. 2022 Dec 14;13:962987. doi: 10.3389/fneur.2022.962987 (PMC9796564; doi:10.3389/fneur.2022.962987)
Supplement: Supplementary file 1 [file Data_Sheet_1.zip › 08 ╒π╚╦├±.pdf]

AF16 浙江省人民医院医学伦理委员会

伦理审查批件

批件编号	2017QX017	项目受理号	QX2017012
项目名称/版本号	取栓器治疗急性缺血性卒中的前瞻性、多中心、单盲、随机对照临床试验		
研究类型	口药物临床试验 ■医疗器械临床试验 口体外诊断试剂临床试验<br>口科研项目 口其他______		
申办单位	微创神通医疗科技（上海）有限公司		
组长单位	上海长海医院		
主要研究者	耿昱	申请专业	神经内科
审查类别	■新方案 口作必要修改后的复审案 口修正案		
审查方式	■会议审查 口快速审查		
审查日期	2017 年 6 月 15 日	审查会议地点	7 号楼第 2 会议室
审查文件	见附件		

投票结果：

同意	作修改后同意	作修改后重审	不同意	终止或暂停
10 票	4 票	0 票	0 票	0 票

审查意见	1、基本符合 GCP 原则，同意进行临床试验。<br>2、医学伦理委员会对该研究实施过程的持续审查：■是 口否<br>审查频度为研究批准之日起：口 3 个月 口 6 个月 ■1 年<br>3、医学伦理委员会有权根据实际进展情况改变持续审查频度。

主任委员签名：______ 日期：______

医学伦理委员会（盖章）

注：

1、研究者应遵循伦理委员会批准的方案执行，本伦理委员会组成运行符合 SFDA GCP 和赫尔辛基宣言的原则。

2、在试验实施过程中，对研究方案和知情同意等相关文件所做的任何修改，均需得到伦理委员会审查同意后方可实施。

3、发生严重不良事件及可能影响风险受益比的任何事件和新信息需及时报告伦理委员会。

4、接受伦理委员会持续审查的项目，请在到期前 1 个月（无论试验开始与否）提出持续审查的申请。

5、如有违背/偏离方案或暂停/提前终止的试验项目，应及时以书面文件报告伦理委员会；临床试验结束后，须及时向伦理委员会提交结题报告。

6、本批件有效期 1 年（自批准之日起），如试验逾期未实施即自行废止。

7、本批件一式四份，分别由申办方、主要研究者、药物临床试验机构和本伦理委员会保存。

附件：

<table>
<tr><td>批件编号</td><td>2017QX017</td><td>项目受理号</td><td>QX2017012</td></tr>
<tr><td>审查文件</td><td colspan="3">
<table>
<tr><td>文件名称</td><td>文件版本</td><td>版本日期</td><td>语言</td></tr>
<tr><td>临床试验方案</td><td>V1.0</td><td>2017-03-08</td><td>中文</td></tr>
<tr><td>受试者知情同意书</td><td>V1.0</td><td>2017-03-08</td><td>中文</td></tr>
<tr><td>主要研究者简历</td><td>无</td><td>无</td><td>中文</td></tr>
<tr><td>病例报告表（CRF）</td><td>V1.0</td><td>2017-03-08</td><td>中文</td></tr>
<tr><td>产品研制符合质量体系声明</td><td>无</td><td>无</td><td>中文</td></tr>
<tr><td>CRO 资质证明</td><td>无</td><td>无</td><td>中文</td></tr>
<tr><td>CRO 委托函</td><td>无</td><td>无</td><td>中文</td></tr>
<tr><td>研究团队及分工</td><td>无</td><td>无</td><td>中文</td></tr>
<tr><td>申办方营业执照、组织机构代码证、税务登记表</td><td>无</td><td>无</td><td>中文</td></tr>
<tr><td>受试者鉴认代码表样张</td><td>无</td><td>无</td><td>中文</td></tr>
<tr><td>中心伦理批件</td><td>无</td><td>无</td><td>中文</td></tr>
<tr><td>试验机构研究条件综述</td><td>无</td><td>无</td><td>中文</td></tr>
<tr><td>伦理审查申请表伦理审查申请表*</td><td>无</td><td>无</td><td>中文</td></tr>
<tr><td>产品检验报告（包括对照产品）</td><td>无</td><td>无</td><td>中文</td></tr>
<tr><td>研究者手册</td><td>V1.0</td><td>2017-03-08</td><td>中文</td></tr>
<tr><td>试验方案签名页</td><td>V1.0</td><td>2017-03-08</td><td>中文</td></tr>
<tr><td>对照品说明书</td><td>无</td><td>无</td><td>中文</td></tr>
<tr><td>取栓器说明书</td><td>无</td><td>无</td><td>中文</td></tr>
<tr><td>产品自检报告</td><td>无</td><td>无</td><td>中文</td></tr>
</table>
</td></tr>
<tr><td colspan="4">浙江省人民医院 医学伦理委员会
浙江省人民医院 医学伦理委员会
医学伦理委员会（盖章）</td></tr>
</table>

浙江省人民医院医学伦理委员会名单

伦理委员会职务	姓名	性别	专业与方向	备注	职称	工作单位
主任委员	于恩彦	男	精神卫生	书记	主任医师	浙江省人民医院
副主任委员	高寒	女	中西医结合	教学部	主任中医师	浙江省人民医院
委员	张红霞	女	财务	财务总监	会计师	浙江省人民医院
委员	吴立萱	女	心内科	门办主任	主任医师	浙江省人民医院
委员	张国兵	男	药学	药学部副主任	副主任药师	浙江省人民医院
委员	陈肖敏	女	护理	护理部主任	主任护师	浙江省人民医院
委员	孙晓东	男	外科学	教学部副主任	主任医师	浙江省人民医院
委员	宋向阳	男	外科学	甲乳外科主任	主任医师	浙江省人民医院
委员	张平	女	设备工程	医学工程部主任	高级工程师	浙江省人民医院
委员	隋梅花	女	分子生物学	肿瘤生物学与创新治疗实验室主任	研究员	浙江省人民医院
委员	杨柳	女	肿瘤学	科研部副主任	副研究员	浙江省人民医院
委员	钱农	女	档案管理	教学部	副主任馆员	浙江省人民医院
委员	常润根	男	法律	律师	律师	北京康达（杭州）律师事务所
委员	毛欣欣	男	医疗器械	社区	高级工程师	社区
委员	连连	女	政治学	副教授	副教授	浙江大学
候补委员	何强	男	肾脏病学	副院长机构主任	主任医师	浙江省人民医院
候补委员	洪朝阳	男	眼科	副院长	主任医师	浙江省人民医院
候补委员	祁金文	男	药学	机构办	副主任药师	浙江省人民医院
候补委员	方晴霞	女	药学	药学部主任	副主任药师	浙江省人民医院
候补委员	舒静	女	妇产科学	主任	主任医师	浙江省人民医院
候补委员	沈俞美	女	法律	律师	律师	北京康达（杭州）律师事务所
秘书	李青青	女	流行病与卫生统计学	科研部	公卫医师	浙江省人民医院
秘书	钱莎莎	女	流行病与卫生统计学	质量管理办公室	公卫医师	浙江省人民医院

浙江省人民医院医学伦理委员会的组成及操作方式严格遵循 GCP（包括 ICH-GCP）及相关法律和法规的规定，实施各项操作规程。

地址：浙江省杭州市上塘路 158 号　邮编：310014　联系电话：0571-85893643　传真：0571-85133255

浙江省人民医院医学伦理委员会

AF15 浙江省人民医院医学伦理委员会

会议记录

时间和地点：2017 年 6 月 13 日 14:00-[illegible]:00　7 号楼 2 号会议室

主持人：于恩彦

参加人员名单：

序号	姓名	职务	签名
1	于恩彦	主任委员	于恩彦
2	高寒	副主任委员	[illegible]
3	张红霞	委员	张红霞
4	吴立萱	委员	吴立萱
5	张国兵	委员	张国兵
6	陈肖敏	委员	陈肖敏
7	孙晓东	委员	孙晓东
8	宋向阳	委员	宋向阳
9	张平	委员	张平
10	隋梅花	委员	隋梅花
11	杨柳	委员	杨柳
12	钱农	委员	钱农
13	常润根	委员	常润根
14	毛欣欣	委员	毛欣欣
15	连连	委员	
16	何强	候补委员	
17	洪朝阳	候补委员	洪朝阳
18	祁金文	候补委员	
19	方晴霞	候补委员	
20	舒静	候补委员	
21	沈俞美	候补委员	
22	李青青	秘书	李青青
23	钱莎莎	秘书	

浙江省人民医院 医学伦理委员会

AF17 浙江省人民医院医学伦理委员会

伦理审查意见函

传达日期	2018 年 11 月 9 日	项目受理号	QX2017012
项目名称/版本号	取栓器治疗急性缺血性卒中的前瞻性、多中心、单盲、随机对照临床试验		
研究类型	□药物临床试验 ■医疗器械临床试验 □体外诊断试剂临床试验<br>□科研项目 □其他______		
申办单位	微创神通医疗科技（上海）有限公司		
组长单位	上海长海医院		
主要研究者	耿昱	申请专业	神经外科
审查类别	□新方案 □复审案 ■修正案 □持续审查 □ SAE/非预期不良事件<br>□违背/偏离方案 □终止/暂停研究 □结题		
审查方式	□会议审查 ■快速审查		
审查日期	2018 年 11 月 9 日	审查会议地点	无
审查文件	1、组长单位伦理批件<br>2、研究方案（修正后的版本号/日期：V2.0/2018-08-08）<br>3、知情同意书（修正后的版本号/日期：V2.0/2018-08-08）<br>4、研究病历（修正后的版本号/日期：V3.0/2018-08-08）<br>5、CRF/eCRF（修正后的版本号/日期：V3.0/2018-08-08）<br>6、研究者手册（修正后的版本号/日期：V2.0/2018-08-08）<br>7、取栓器说明书（修正后的版本号/日期：V2.0/NA）		
审查意见	经本医学伦理委员会审查，同意上述文件的修订，同意试验继续进行。		

主任委员签名：[signature]　　日期：2018.11.9

医学伦理委员会（盖章）　浙江省人民医院医学伦理委员会

注：

请根据上述审查意见对方案和知情同意书作修改后，递交医学伦理委员会审查批准后执行。如果对审查意见有不同观点，请书面向本伦理委员会秘书处反映。
