# Supplementary material for: Endovascular treatment of acute ischemic stroke with a fully radiopaque retriever: A randomized controlled trial
Source: Front Neurol. 2022 Dec 14;13:962987. doi: 10.3389/fneur.2022.962987 (PMC9796564; doi:10.3389/fneur.2022.962987)
Supplement: Supplementary file 1 [file Data_Sheet_1.zip › 09 ╝├─■╩╨╥╗.pdf]

济宁市第一人民医院医学伦理委员会

伦理审查批件

伦理审查编号：（2017-械-02）

项目名称	取栓器治疗急性缺血性卒中的前瞻性、多中心、单盲、随机对照临床试验				
CFDA 批件号	-				
申办者	微创神通医疗科技（上海）有限公司				
临床研究科室	神经内科	主要研究者	初建峰		
送审文件	具体见随附“济宁市第一人民医院医学伦理委员会审查文件清单”				

依据 GCP 要求及相关法规，本伦理委员会的组织和实施相对独立
本伦理委员会的人员组成和工作程序符合中国 GCP 以及国家相关规定

伦理审查方式：☑会议审查　　□快速审查

审查时间：2017 年 11 月 15 日		会议地点：济宁市第一人民医院办公楼四楼会议室					
审查委员	见随附“伦理委员会会议签到表”						
伦理委员会总人数	15	出席人数	11	回避人数	0	弃权人数	1
投票结果	同意 10 票		不同意 0 票		作必要修改后同意 0 票		
	作必要修改后重审 0 票		终止或暂停试验 0 票		回避 0 票		
审查意见	1、经本伦理委员会审查：同意进行该临床研究。<br>意见和建议：无<br>2、该研究进行过程中将接受伦理委员会的定期跟踪审查？☑是　□否<br>定期跟踪审查频率：□6 个月　☑12 个月　其他 ______						

注意事项：

1、本项临床试验应当在伦理委员会同意进行之日起 1 年内实施。预期未实施的，本批件自行废止。
2、研究应遵循本伦理委员会批准的方案执行，须符合 CFDA/GCP 和《赫尔辛基宣言》的原则。
3、研究过程中，对研究方案和知情同意书等相关文件所作的任何修改，请交《修正案申请表》及“送审文件清单”中规定相关资料，并得到伦理委员会审查同意该修正后方可实施。
4、发生严重不良事件或影响研究风险受益比的非预期不良事件，在向 CFDA 上报的同时向伦理委员会作书面通报。伦理委员会有权根据对其评估做出新的决定。

主任/副主任委员签字：[signature]

盖章：

日期：

伦理委员会地址：济宁市任城区健康路 6 号　邮编：272011　联系电话：0537-2253449

济宁市第一人民医院

医学伦理委员会会议签到

时　　间：2017 年 11 月 15 日

会议地点：济宁市第一人民医院办公楼四楼会议室

会议签到表

成员	性别	职称	专业	委员会任职	单位	签名
孙树印	男	主任医师	急诊医学	主任委员	济宁市第一人民医院	孙树印
乔　森	男	主任医师	外科学	副主任委员	济宁市第一人民医院	乔森
郭洪敏	男	主任医师	外科学	副主任委员	济宁市第一人民医院	缺席
刘瑞娟	女	主任医师	呼吸内科	委员	济宁市第一人民医院	刘瑞娟
齐先龙	男	主任医师	影像学	委员	济宁市第一人民医院	缺席
鹿占鹏	男	主任医师	泌尿外科	委员	济宁市第一人民医院	缺席
孙秀芹	女	主任医师	生殖医学	委员	济宁市第一人民医院	孙秀芹
徐红琳	女	主任药师	药学	委员	济宁市第一人民医院	徐红琳
杨文红	女	主任护师	护理学	委员	济宁市第一人民医院	缺席
史继红	女	副主任医师	心理学	委员	济宁市第一人民医院	史继红
房宝梅	女	副主任护师	护理学	委员	济宁市第一人民医院	房宝梅
刘传新	男	教授	伦理学	委员	济宁医学院	刘传新
毕于建	男	教授	社会学	委员	济宁医学院	毕于建
陈　旭	男	律师	法学	委员	山东中评和律师事务所	陈旭
杜　凌	女	社会监督员	群众代表	委员	济宁市牌坊街居委	杜凌

济宁市第一人民医院

伦理审查清单

伦理审查项目编号：2017-械-02

1、 国家食品药品监督管理部门/医疗器械监督管理部门批准的检测报告

2、 主要研究者简历

3、 取栓器自测报告

4、 研究方案（版本号：1.0，版本日期：2017-03-08）

5、 取栓器临床试验知情同意书（版本号：1.0，版本日期：2017-03-08）

6、 受试者招募说明

7、 临床研究保险

8、 原始病历（版本号：1.0，版本日期：2017-03-08）

9、 病例报告表（版本号：1.0，版本日期：2017-03-08）

10、 研究者手册（版本号：1.0，版本日期：2017-03-08）

11、 取栓器说明书

12、 中心伦理批件

13、 申办方资质证明

14、 CRO 公司资质证明

15、 委托函（申办方对 CRO,CRO 对本中心，CRO 对 CRA）

16、 专业组项目研究团队说明

伦理委员会地址：济宁市健康路 6 号济宁市第一人民医院办公楼二楼
联系方式：0537-2253449

机构递交信

尊敬的济宁市第一人民医院神经内科初建峰主任：

在“取栓器治疗急性缺血性卒中的前瞻性、多中心、单盲、随机对照临床试验”的多中心临床研究中(方案编号:2016-GATOR-01-A)，根据医疗器械临床试验相关法规以及贵院临床试验机构办公室的要求递交如下材料，敬请提交贵单位临床试验机构办公室审阅。

1、研究方案（版本号：2.0，版本日期：2018-08-08）
2、研究方案 V2.0 修订说明及修订列表
3、知情同意书（版本号：2.0，版本日期：2018-08-08）
4、知情同意书 V2.0 修订说明及列表
5、研究者手册（版本号：2.0，版本日期：2018-08-08）
6、研究者手册 V2.0 修订说明及列表
7、病例报告表（版本号：3.0，版本日期：2018-08-08）
8、病例报告表 V3.0 修订说明及列表
9、原始病历（版本号：3.0，版本日期：2018-08-08）
10、 原始病历 V3.0 修订说明及列表
11、 取栓器说明书（版本号：2.0，文件编号：A-T0006-002）
12、 取栓器说明书 V2.0 修订说明及列表
13、 SAE 豁免说明-20180914
14、 长海医院-方案修正等快审批件-20180925
15、 血糖采集说明-201810

此致

敬礼！

方恩（天津）医药发展有限公司

临床监查员签名：王世栋

日期：2018.10.23

回执

我已收到上述递交文件，将尽快提交我单位临床试验机构办公室审查。

济宁市第一人民医院神经内科

研究者签名：[illegible]

接收日期：2018.10.23

机构递交信

尊敬的济宁市第一人民医院临床试验机构办公室：

在“取栓器治疗急性缺血性卒中的前瞻性、多中心、单盲、随机对照临床试验”的多中心临床研究中(方案编号:2016-GATOR-01-A)，根据医疗器械临床试验相关法规以及我院临床试验机构办公室的要求递交以下资料，敬请接收审阅。

1、研究方案（版本号：2.0，版本日期：2018-08-08）
2、研究方案 V2.0 修订说明及修订列表
3、知情同意书（版本号：2.0，版本日期：2018-08-08）
4、知情同意书 V2.0 修订说明及列表
5、研究者手册（版本号：2.0，版本日期：2018-08-08）
6、研究者手册 V2.0 修订说明及列表
7、病例报告表（版本号：3.0，版本日期：2018-08-08）
8、病例报告表 V3.0 修订说明及列表
9、原始病历（版本号：3.0，版本日期：2018-08-08）
10、 原始病历 V3.0 修订说明及列表
11、 取栓器说明书（版本号：2.0，文件编号：A-T0006-002）
12、 取栓器说明书 V2.0 修订说明及列表
13、 SAE 豁免说明-20180914
14、 长海医院-方案修正等快审批件-20180925
15、 血糖采集说明-201810

此致

敬礼！

济宁市第一人民医院神经内科

主要研究者签名：[signature]

日期：2018.10.23

回　执

临床试验机构办公室已收到取栓器治疗急性缺血性卒中的前瞻性、多中心、单盲、随机对照临床试验（方案编号：2016-GATOR-01-A）项目的递交材料，我们将会：

☑ 同意上述文件在本研究中使用，并归档保存;

☐ 其他，说明：

临床试验机构办公室秘书签字：[signature]

日期：2018.10.23

伦理递交信

尊敬的济宁市第一人民医院神经内科初建峰主任：

在“取栓器治疗急性缺血性卒中的前瞻性、多中心、单盲、随机对照临床试验”的多中心临床研究中(方案编号:2016-GATOR-01-A)，根据医疗器械临床试验相关法规以及贵院伦理委员会的要求递交如下材料，敬请提交贵单位伦理委员会审阅。

1、研究方案（版本号：2.0，版本日期：2018-08-08）
2、研究方案 V2.0 修订说明及修订列表
3、知情同意书（版本号：2.0，版本日期：2018-08-08）
4、知情同意书 V2.0 修订说明及列表
5、研究者手册（版本号：2.0，版本日期：2018-08-08）
6、研究者手册 V2.0 修订说明及列表
7、病例报告表（版本号：3.0，版本日期：2018-08-08）
8、病例报告表 V3.0 修订说明及列表
9、原始病历（版本号：3.0，版本日期：2018-08-08）
10、 原始病历 V3.0 修订说明及列表
11、 取栓器说明书（版本号：2.0，文件编号：A-T0006-002）
12、 取栓器说明书 V2.0 修订说明及列表
13、 SAE 豁免说明-20180914
14、 长海医院-方案修正等快审批件-20180925
15、 血糖采集说明-201810

此致

敬礼！

方恩（天津）医药发展有限公司

临床监查员签名：王世艳

日期：2018.10.23

回执

我已收到上述伦理递交文件，将尽快提交我单位伦理委员会审查。

济宁市第一人民医院神经内科

研究者签名：[illegible]

接收日期：2018.10.23

伦理递交信

尊敬的济宁市第一人民医院伦理委员会：

在“取栓器治疗急性缺血性卒中的前瞻性、多中心、单盲、随机对照临床试验”的多中心临床研究中(方案编号:2016-GATOR-01-A)，根据医疗器械临床试验相关法规以及我院伦理委员会的要求递交以下资料，请接收审阅。

1、研究方案（版本号：2.0，版本日期：2018-08-08）

2、研究方案 V2.0 修订说明及修订列表

3、知情同意书（版本号：2.0，版本日期：2018-08-08）

4、知情同意书 V2.0 修订说明及列表

5、研究者手册（版本号：2.0，版本日期：2018-08-08）

6、研究者手册 V2.0 修订说明及列表

7、病例报告表（版本号：3.0，版本日期：2018-08-08）

8、病例报告表 V3.0 修订说明及列表

9、原始病历（版本号：3.0，版本日期：2018-08-08）

10、 原始病历 V3.0 修订说明及列表

11、 取栓器说明书（版本号：2.0，文件编号：A-T0006-002）

12、 取栓器说明书 V2.0 修订说明及列表

13、 SAE 豁免说明-20180914

14、 长海医院-方案修正等快审批件-20180925

15、 血糖采集说明-201810

此致

敬礼！

济宁市第一人民医院神经内科

主要研究者签名：[signature]

日期：2018.10.23

回　执

伦理委员会已收到取栓器治疗急性缺血性卒中的前瞻性、多中心、单盲、随机对照临床试验（方案编号：2016-GATOR-01-A）项目的伦理递交材料，我们将会：

- ☐ 择期进行会议审查，之后书面回复审查意见
- ☐ 进行快速审查，之后书面回复审查意见
- ☒ 备案并存档，同意在本研究中使用
- ☐ 其他，说明：

伦理秘书签字：[illegible]

日期：2018.10.23
