# Supplementary material for: Endovascular treatment of acute ischemic stroke with a fully radiopaque retriever: A randomized controlled trial
Source: Front Neurol. 2022 Dec 14;13:962987. doi: 10.3389/fneur.2022.962987 (PMC9796564; doi:10.3389/fneur.2022.962987)
Supplement: Supplementary file 1 [file Data_Sheet_1.zip › 11 ╒─╓▌╩╨╥╜.pdf]

伦理审查意见

编号：IEC-SG-28-1

意见号：201701001

<table>
<tr><td>试验项目名称</td><td colspan="3">取栓器治疗急性缺血性卒中的前瞻性、多中心、单盲、随机对照临床试验</td></tr>
<tr><td>申办单位</td><td colspan="3">微创神通医疗科技（上海）有限公司</td></tr>
<tr><td>研究单位</td><td colspan="3">福建省漳州市医院</td></tr>
<tr><td>主要研究者</td><td>神经内科 陈文伙</td><td>审查方式</td><td>☑会议审查
☐快速审查</td></tr>
<tr><td>审查类别</td><td colspan="3">☑初次审查 ☐修正案审查 ☐跟踪审查 ☐违背方案审查
☐严重不良事件审查 ☐暂停/终止研究审查 ☐结题审查</td></tr>
<tr><td>审查文件</td><td colspan="3">审批文件
1、 临床试验方案 V1.0 版，版本日期 2017-3-8
2、 知情同意书 V1.0 版，版本日期 2017-3-8
3、 病例报告表 V1.0 版，版本日期 2017-3-8
4、 研究者手册 V1.0 版，版本日期 2017-3-8
5、 研究团队名单及履历
6、 受试者招募说明

审阅文件
1、 上海长海医院伦理委员会批准函（组长单位）
2、 医疗器械经营许可证
3、 医疗器械生产企业许可证
4、 申办方营业执照
5、 CRO 公司营业执照：方恩（天津）医药发展有限公司
6、 临床试验委托书（申办方对 CRO、申办方对本单位）
7、 医疗器械说明书
8、 检验报告
9、 临床试验协议范本
10、 临床试验责任保险（索赔发生制）</td></tr>
<tr><td>本伦理委员会联系方式</td><td colspan="3">地址：福建省漳州市医院 行政楼 9 楼科教科
电话：0596-2082955</td></tr>
<tr><td>到会委员名单</td><td colspan="3">详见附表</td></tr>
</table>

伦理委员会审查意见

☐同意

是否需要持续审查：☐是　☐否　审查频率：☐3 个月　☐6 个月　☐12 个月

☑作必要的修正后同意　　☐做必要的修正后重审

☐ 不同意　　☐ 终止/暂停已经批准的研究

意见及建议：☐无　☑有

1、知情同意书中的英文单词均应有翻译或必要的解释，以便于受试者理解。例如 Solitaire FR（ev3/Medtronic、mRS/NIHSS、TICI 等。

2、知情同意书中第九点，第三行“导致了经医疗鉴定的与试验支架有关的直接损害”，不符合《涉及人的生物医学研究伦理审查办法》中的“依法赔偿原则”；同时，2016 年 6 月 1 日生效的《医疗器械临床试验质量管理规范》中对此的相关要求有第十六条、二十二条、四十八条，例如：如发生与试验相关的伤害，受试者可以获得治疗和经济补偿。

3、方案第 34 页中“特殊情况下的知情同意过程”，对于“危机生命的紧急情况”的受试者应谨慎纳入研究，注意防范和控制风险。

按审查意见修改后的文件或对审查意见不同观点的陈述，请提交“复审申请”，方案/知情同意书修改请注明新的版本号和版本日期，报伦理委员会审查，经过批准后执行。

主任委员签名	[illegible]
日期	2017.9.6
伦理委员会	福建省漳州市医院医学伦理委员会

福建省漳州市医院医学伦理委员会

伦理审查批件

编号：IEC-SG-29-1

批件号：201701002

<table>
<tr><td>试验项目名称</td><td colspan="3">取栓器治疗急性缺血性卒中的前瞻性、多中心、单盲、随机对照临床试验</td></tr>
<tr><td>申办单位</td><td colspan="3">微创神通医疗科技（上海）有限公司</td></tr>
<tr><td>研究单位</td><td colspan="3">福建省漳州市医院</td></tr>
<tr><td>主要研究者</td><td>神经内科　陈文伙</td><td>审查方式</td><td>☐会议审查
☑快速审查</td></tr>
<tr><td>审查文件</td><td colspan="3">审批文件
1、 复审申请及说明、修改清单（2017.9.21）
2、 临床试验方案 V1.0 版，版本日期 2017-3-8
3、 知情同意书 V2.0 版，版本日期 2017-9-12
4、 病例报告表 V1.0 版，版本日期 2017-3-8
5、 研究者手册 V1.0 版，版本日期 2017-3-8
6、 研究团队名单及履历
7、 受试者招募说明
审阅文件
1、 上海长海医院伦理委员会批准函（组长单位）
2、 医疗器械经营许可证
3、 医疗器械生产企业许可证
4、 申办方营业执照
5、 CRO 公司营业执照：方恩（天津）医药发展有限公司
6、 临床试验委托书（申办方对 CRO、申办方对本单位）
7、 医疗器械说明书、检验报告
8、 临床试验协议范本
9、 临床试验责任保险（索赔发生制）</td></tr>
<tr><td>本伦理委员会联系方式</td><td colspan="3">地址：福建省漳州市医院 9 楼科教科
电话：0596-2082955</td></tr>
<tr><td>到会委员名单</td><td colspan="3">不适用</td></tr>
<tr><td colspan="4">伦理委员会审查意见
经本伦理委员会审查，同意按所批准的临床研究方案、知情同意书开展本项研究，请遵循 GCP 的原则及本伦理委员会批准的方案开展临床研究，保护受试者的权益。
研究过程中发生主要研究者变更，临床试验方案、知情同意书的修改，应提交修正案审查申请。
按规定的年度/定期跟踪审查频率，及时提前 1 月递交研究进展报告。
试验过程中若发生违背试验方案或 GCP 原则，及时提交违背方案报告。
申请人暂停或提前终止临床研究，及时提交暂停/终止研究报告。
试验完成，应递交结题报告。</td></tr>
<tr><td>审查频率</td><td colspan="3">☐3 个月　☐6 个月　☑12 个月</td></tr>
<tr><td>主任委员签名</td><td colspan="3">[illegible]</td></tr>
<tr><td>日期</td><td colspan="3">2017.10.27</td></tr>
<tr><td>伦理委员会</td><td colspan="3">福建省漳州市医院伦理委员会（盖章）</td></tr>
</table>

会议签到表 编号：IEC-SG-23-3

声 明

本伦理委员会是独立的。委员会的职责、人员组成、操作规程遵循中国 GCP 规范和 ICH-GCP 的伦理审查原则，并遵守中国的相关法律及法规。

伦理委员会名称	福建省漳州市医院临床试验伦理委员会			
会议日期	2017.9.4			
姓名	性别	单位/专业背景	职务	签到
陈诺琦	男	漳州市医院/内分泌与代谢疾病	主任委员	
林玉霜	女	漳州市医院/麻醉	副主任委员	
叶小玲	女	漳州市医院/儿科	委员	
林瑞生	男	漳州市医院/神经外科	委员	
吴燕华	女	漳州市医院/呼吸内科	委员	
陈开珠	女	漳州市医院/护理	委员	—
许 慎	女	漳州市医院/肿瘤内科	委员	
邱陆阵	男	漳州市医院/心血管内科	委员	
陈锦凤	女	漳州市医院/内分泌与代谢疾病	委员	
冯惠平	女	漳州市医院/药学	委员	
薛贵滨	男	闽南师范大学/法学	委员	—
黄耀明	男	闽南师范大学/社会工作	委员	—
戴学清	男	漳州市医保中心/管理	委员	—
詹丽英	女	西街社区/群众代表	委员	
谢素红	女	东铺头社区委员会	委员	
蒋辉	男	漳州市医院/卫生管理	委员兼秘书	

注：本届委员会成员任期三年，从 2016 年 9 月 18 日到 2019 年 9 月 17 日。

伦理审查意见

编号：IEC-SG-28-1

意见号：201701001

试验项目名称	取栓器治疗急性缺血性卒中的前瞻性、多中心、单盲、随机对照临床试验		
申办单位	微创神通医疗科技（上海）有限公司		
研究单位	福建省漳州市医院		
主要研究者	神经内科 陈文伙	审查方式	☑会议审查<br>☐快速审查
审查类别	☐初次审查　☑修正案审查　☑跟踪审查　☐违背方案审查<br>☐严重不良事件审查　☐暂停/终止研究审查　☐结题审查		
审查文件	1. 肝功能、SCIH、时间窗豁免说明，授权书（2018.7.23）<br>2. 研究进展报告（2018.9.26）<br>3. 修正案申请（2018.10.19）<br>(1)研究方案 2.0 版，日期 2018.8.8；<br>(2)知情同意书 3.0 版，日期 2018.9.9。		
本伦理委员会联系方式	地址：福建省漳州市医院 行政楼 8 楼 803 室<br>电话：0596-2082561		
到会委员名单	详见附表		
伦理委员会审查意见			
☑同意<br>是否需要持续审查：☑是　☐否　审查频率：☐3 个月　☐6 个月　☑12 个月<br>☐作必要的修正后同意　☐做必要的修正后重审<br>☐ 不同意　☐ 终止/暂停已经批准的研究<br>意见及建议：☑无　☐有<br>按审查意见修改后的文件或对审查意见不同观点的陈述，请提交“复审申请”，方案/知情同意书修改请注明新的版本号和版本日期，报伦理委员会审查，经过批准后执行。			
主任委员签名	[illegible]		
日期	2018.11.9		
伦理委员会	福建省漳州市医院医学伦理委员会（盖章）		
