# Supplementary material for: Endovascular treatment of acute ischemic stroke with a fully radiopaque retriever: A randomized controlled trial
Source: Front Neurol. 2022 Dec 14;13:962987. doi: 10.3389/fneur.2022.962987 (PMC9796564; doi:10.3389/fneur.2022.962987)
Supplement: Supplementary file 1 [file Data_Sheet_1.zip › 12 ╣─┬Ñ.pdf]

伦理审查意见

意见号	2017-165-01		
项目名称	取栓器治疗急性缺血性卒中的前瞻性、多中心、单盲、随机对照临床试验		
项目来源	微创神通医疗科技（上海）有限公司		
研究单位	南京大学医学院附属鼓楼医院神经内科		
主要研究者	徐运		
审查类别	初始审查	审查方式	会议审查
审查日期	2017 年 11 月 22 日	审查地点	机关楼 112 会议室
审查委员	详见会议签到单		
审查文件	1. 初始审查申请<br>2. 申办者资质证明：企业法人营业执照<br>3. CRO 资质证明<br>4. 申办者委托 CRO 进行药物临床试验的委托书<br>5. 临床研究方案 V1.0，2017-03-08<br>6. 知情同意书 V1.0，2017-03-08<br>7. 病例报告表和原始病历 V2.0，2017-11-06<br>8. 研究者手册 V1.0，2017-03-08<br>9. 医疗器械说明书（取栓器及对照品）<br>10. 产品技术要求<br>11. 产品质量检测报告（注册检验报告和自检报告）<br>12. 医疗器械动物实验报告<br>13. 主要研究者专业履历<br>14. 监查员委托书及身份证复印件<br>15. 临床试验机构的设施和条件能够满足试验的综述<br>16. 试验用医疗器械的研制符合适用的医疗器械质量管理体系相关要求的声明<br>17. 组长单位批件<br>18. 其他：保险合同		

审查意见

根据国家卫计委《涉及人的生物医学研究伦理审查办法》(2016)、CFDA《药物临床试验质量管理规范（2003)》、《医疗器械临床试验规定（2016)》、WMA《赫尔辛基宣言》(2013 年）和 CIOMS《人体生物医学研究国际道德指南》的伦理原则，经本伦理委员会审查，意见如下：

临床研究方案：

研究者的学术地位、医疗水平有可靠的保障。从本中心资质来说，神经内科是药物临床试验专业，研究团队经过 GCP 培训，有丰富的临床诊疗和临床试验经验。本研究设计研究目的明确、研究背景清

晰、研究流程详细、研究步骤规范，研究的入排标准比较科学严谨的。主要研究者已经声明了和该项研究没有利益冲突。研究涉及弱势群体，用已上市的器械多对照，研究安全有基本的保证，保护受试者措施得当，风险受益比是合理的。但需要补充提供以下材料：

1.　请补充提供对照组取栓器医疗器械注册证。

2.　研究方案附录的“多中心临床试验的所有临床试验机构和研究者列表”中没有鼓楼医院；

知情同意书：

从知情同意书来看，要素是完备的，包含了研究背景、目的、研究方法、研究流程、研究范围、研究的受益和风险、研究的损害赔偿、隐私保密等均包含了，需要修改以下内容：

1.英文较多，请用中文通俗易懂地给予解释；

2.在可能的受益部分，“可以恢复”改为“可能恢复”；

因受试者来院时多为昏迷状态，请与受试者家属充分做好知情同意工作，并在受试者清醒后再予以告知。

经伦理委员会讨论决定，审查意见为作必要修正后同意。

按审查意见修改后的文件，或对审查意见不同观点的陈诉，请提交“复审申请”，方案/知情同意书请注明新的版本号和版本日期，并以阴影和/或下划线方式标注修改部分，报伦理委员会审查，经批准后执行。

**调整的年度/定期跟踪审查频率**	12 个月
**伦理委员会**	南京大学医学院附属鼓楼医院医学伦理委员会
**主任委员签字**	[illegible]
**日期**	2017.11.22

南京大学医学院附属鼓楼医院医学伦理委员会

伦理委员会签到单

伦理委员会名称/地址：南京大学医学院附属鼓楼医院伦理委员会

南京市中山路 321 号，210008

时间：2017-11-22

职责	姓名	职务	专业	性别	单位	签名
主任委员	何忠正	主任医师	外科学	男	南京医院协会	
副主任委员	胡娅莉	主任医师	妇产科学	女	南京大学医学院附属鼓楼医院	
委员	傅国藩	主任医师	外科学	男	南京宁益眼科医院	
委员	戴令娟	主任医师	内科学	女	南京大学医学院附属鼓楼医院	
委员	方　芸	主任药师	药学	女	南京大学医学院附属鼓楼医院	
委员	王　芃	副教授	宗教	女	金陵协和神学院	
委员	许碧云	研究员	统计学	女	南京大学医学院附属鼓楼医院	
委员	孙小玲	助理社会工作师	社会学	女	华侨路街道龙蟠里社区	
委员	陈隆典	主任医师	内科学	男	南京大学医学院附属鼓楼医院	
委员	丁从珠	主任医师	内科学	女	南京大学医学院附属鼓楼医院	
委员	胡　云	主任医师	内科学	女	南京大学医学院附属鼓楼医院	
委员	王志群	主任医师	妇产科学	女	南京大学医学院附属鼓楼医院	
委员	李　娟	主任医师	血液病学	女	南京大学医学院附属鼓楼医院	
委员	顾光煜	主任技师	检验医学	男	南京大学医学院附属鼓楼医院	
委员	王　玲	初级社工师	经济管理	女	南京乐德特殊儿童康复中心	
委员、秘书	沙莉莉	助理研究员	管理学	女	南京大学医学院附属鼓楼医院	

南京大学医学院附属鼓楼医院医学伦理委员会

南京大学医学院附属鼓楼医院
医学伦理委员会伦理审查批准件

IRB Review Approval Documents

编号：2017-165-02

**研究名称**	取栓器治疗急性缺血性卒中的前瞻性、多中心、单盲、随机对照临床试验		
**研究方案号**	2016-GATOR-01-A	**项目起止时间**	2016 年 9 月-2019 年 12 月
**申办者/ CRO 公司**	微创神通医疗科技（上海）有限公司		
**组长单位**	上海长海医院	**CFDA 批件号**	NA
**申请专业**	神经内科	**主要研究者**	徐运
**研究期别**	□I 期 □II 期 □III 期 □IV 期 ■医疗器械临床试验 □体外诊断试剂 □科研		
**审查途径**	快速审查	**审查类型**	复审
**报告时间**	2017 年第 16 次会议	**审查地点**	NA
**伦理委员会列席人员签名**	NA		

伦理审查文件：

1. 复审申请
2. 申办者资质证明：企业法人营业执照
3. CRO 资质证明
4. 申办者委托 CRO 进行药物临床试验的委托书
5. 临床研究方案 V1.0，2017-03-08
6. 知情同意书 V2.0，2017-11-30
7. 病例报告表和原始病历 V2.0，2017-11-06
8. 研究者手册 V1.0，2017-03-08
9. 医疗器械说明书（取栓器及对照品）
10. 产品技术要求
11. 产品质量检测报告（注册检验报告和自检报告）
12. 医疗器械动物实验报告
13. 主要研究者专业履历
14. 监查员委托书及身份证复印件
15. 临床试验机构的设施和条件能够满足试验的综述
16. 试验用医疗器械的研制符合适用的医疗器械质量管理体系相关要求的声明
17. 组长单位批件
18. 其他：保险合同

南京大学医学院附属鼓楼医院医学伦理委员会

伦理委员会审评意见

声明：（请仔细阅读）

1. 该批件有效期为 3 年，超过效期请继续提出申请。
2. 已批准项目须遵循本伦理委员会批准的方案执行，该伦理委员会组成及运行符合 CFDA-GCP/ICH-GCP 和《赫尔辛基宣言》的原则。
3. 对已批准的临床研究方案、知情同意书等材料的任何修改及主要研究者更换等。请及时向本伦理委员会提交《修正案申请》，获得本伦理委员会书面批准后方可执行。
4. 发生严重不良事件及影响研究风险受益比的非预期事件，请及时报告本伦理委员会。
5. 暂停/提前终止临床研究，请及时通知伦理审查委员会。
6. 发现违反试验方案情况须及时报告本伦理委员会。
7. 根据本伦理委员会对持续审查频度的意见，无论试验开始与否，请在持续审查日到期前一个月提出持续审查的申请。申办者应向组长单位提交各中心研究进展汇总报告。
8. 完成临床研究，请提交结题报告供本伦理委员会审查。
9. 南京大学医学院附属鼓楼医院医学伦理委员会 地址：南京市中山路 321 号，210008
   联系电话：025-66056260,传真：025-66056260
   Email:gyethics@163.com

1. 伦理委员会对项目审查决定为同意开展该项研究。

2. 国际多中心研究对我国人类遗传资源采集、收集、研究、开发、买卖、出口、出境等，须取得中国人类遗传资源管理办公室批准后方可实施。

3. 持续审查

本伦理委员会对此项研究的持续审查频率为自研究批准之日起：□3 个月　　□6 个月　　■1 年

除非研究在周期内已经完成，否则请在 2018 年 12 月 26 日之前一个月内向伦理委员会递交年度进展报告。本伦理委员会具有根据实际进展情况改变持续审查频度的权力。

4. 请仔细阅读审批件声明页内容，方案批准后有声明所述情况发生，请及时递交相关审查申请。

主任委员签名：

日期：2017.12.26

南京大学医学院附属鼓楼医院医学伦理委员会（盖章）

南京大学医学院附属鼓楼医院
医学伦理委员会伦理审查批准件

IRB Review Approval Documents

编号：2017-165-03

**研究名称**	取栓器治疗急性缺血性卒中的前瞻性、多中心、单盲、随机对照临床试验		
**研究方案号**	2016-GATOR-01-A	**项目起止时间**	2016.9-2019.12
**申办者/CRO 公司**	微创神通医疗科技（上海）有限公司/方恩（天津）医药发展有限公司/		
**组长单位**	上海长海医院	**临床试验批件号**	NA
**申请专业**	神经内科	**主要研究者**	徐运
**研究期别**	□I 期 □II 期 □III 期 □IV 期 ■医疗器械临床试验□科研		
**审查途径**	□会议审查　■快速审查	**审查类型**	□首次审查 □复审 ■跟踪审查
**会议日期/报告时间**	2018 年 10 月 31 日	**审查地点**	NA
**伦理委员会列席人员签名**	无		

伦理审查文件：

1. 修正案审查申请
2. 临床研究方案修正说明页
3. 修正的临床研究方案　（版本号：V2.0，版本日期：2018 年 08 月 08 日）
4. 修正的临床研究方案　（版本号：V2.0，版本日期：2018 年 08 月 08 日）
5. 修正的知情同意书　（版本号：V3.0，版本日期：2018 年 09 月 09 日）
6. 修正的知情同意书　（版本号：V3.0，版本日期：2018 年 09 月 09 日）
7. 组长单位伦理审查批件
8. 研究者手册 无痕版
9. 研究者手册 痕迹版
10. 研究者手册修订列表
11. 知情同意书修订列表
12. 病例报告表 无痕版
13. 病例报告表 痕迹版
14. 病例报告表修订列表
15. 原始病历 无痕版
16. 原始病历 痕迹版
17. 原始病历修订列表
18. 取栓器项目使用说明 无痕版
19. 取栓器项目使用说明书 痕迹版

南京大学医学院附属鼓楼医院 医学伦理委员会

20.取栓器使用说明书修订列表
**伦理委员会审评意见**

声明：（请仔细阅读）

1.批件有效期为 2017 年 12 月 26 日~2018 年 12 月 26 日，超过效期请继续提出申请。

2.已批准项目须遵循本伦理委员会批准的方案执行，本伦理委员会组成及运行符合 CFDA-GCP/ICH-GCP 和《赫尔辛基宣言》的原则。

3. 对已批准的临床研究方案、知情同意书等材料的任何修改及主要研究者更换等。请及时向本伦理委员会提交《修正案申请》，获得本伦理委员会书面批准后方可执行。

4.发生严重不良事件及影响研究风险受益比的非预期事件，请及时报告本伦理委员会。

5.暂停/提前终止临床研究，请及时通知伦理审查委员会。

6.发现违反试验方案情况须及时报告本伦理委员会。

7.根据本伦理委员会对持续审查频度的意见，无论试验开始与否，请在持续审查日到期前一个月提出持续审查的申请。申办者应向组长单位提交各中心研究进展汇总报告。

8.完成临床研究，请提交结题报告供本伦理委员会审查。

9.南京大学医学院附属鼓楼医院医学伦理委员会 地址：南京市中山路 321 号，210008

联系电话：025-66056260,传真：025-66056260

Email:gyethics@163.com

1.伦理委员会对项目审查决定为同意开展该项研究。

2.国际多中心研究对我国人类遗传资源采集、收集、研究、开发、买卖、出口、出境等，须取得中国人类遗传资源管理办公室批准后方可实施

3.持续审查

本伦理委员会对此项研究的持续审查频率为自研究批准之日起：☐3 个月　☐6 个月　■1 年

除非研究在周期内已经完成，否则请在 2019 年 12 月 26 日之前一个月内向伦理委员会递交年度进展报告。本伦理委员会具有根据实际进展情况改变持续审查频度的权力。

4.请仔细阅读审批件声明页内容，方案批准后有声明所述情况发生，请及时递交相关审查申请。

主任委员签名：[signature]

日期：**2018 年 10 月 31 日**

南京大学医学院附属鼓楼医院医学伦理委员会（盖章）
