# Supplementary material for: Endovascular treatment of acute ischemic stroke with a fully radiopaque retriever: A randomized controlled trial
Source: Front Neurol. 2022 Dec 14;13:962987. doi: 10.3389/fneur.2022.962987 (PMC9796564; doi:10.3389/fneur.2022.962987)
Supplement: Supplementary file 1 [file Data_Sheet_1.zip › 13 ╟α┤≤╥╜.pdf]

青岛大学附属医院医学伦理委员会

伦理审查意见

意见号	器临伦审意见 QYFYECYJ 2018-002-01		
项目名称	取栓器治疗急性缺血性卒中的前瞻性、多中心、单盲、随机对照临床试验		
项目来源	申办方：微创神通医疗科技（上海）有限公司<br>CRO：方恩（天津）医药发展有限公司		
研究单位	神经内科专业		
主要研究者	张勇		
审查类别	初始审查	审查方式	会议审查
审查日期	2018 年 5 月 9 日	审查地点	行政楼四楼会议室
审查委员	蒋光峰、张国庆、张晓春、孙伟、于壮、孔心涓、牛珉、刘淑红、孙立荣、曲政海、冷萍、 吴力群、李环廷、肖兴国、崔竹梅、 阎胜利、 阎博民、隋忠国、蔡霞		
审查文件	1. 医疗器械产品技术要求<br>2. 临床试验方案 版本号：第 1.0 版 版本日期：2017 年 3 月 8 日<br>3. 研究者手册 版本号：第 1.0 版 版本日期：2017 年 3 月 8 日<br>4. 病例报告表 版本号：第 2.0 版 版本日期：2017 年 11 月 6 日<br>5. 原始病历 版本号：第 2.0 版 版本日期：2017 年 11 月 6 日<br>6. 知情同意书 版本号：第 1.0 版 版本日期：2017 年 3 月 8 日<br>7. 国家食品药品监督管理局上海医疗器械质量监督检验中心检验报告<br>报告编号： 国医检（械）字 ZC2016 第 637 号<br>国医检（械）字 ZC2016 第 636 号<br>8. 取栓器自测报告 文件编号：050016B<br>9. 试验产品的动物试验报告<br>10. 主要研究者履历<br>11. 生命科学人体临床试验责任保险 保单号：92666573		

审查意见

根据卫计委《涉及人的生物医学研究伦理审查办法（2016）》、CFDA《药物临床试验质量管理规范（2003）》、《医疗器械临床试验质量管理规范（2016）》、WMA《赫尔辛基宣言》和 CIOMS《人体生物医学研究国际道德指南》的伦理原则，经本伦理委员会审查，意见如下：

做必要修正后重审。

1. 根据试验方案第 15 页**“入组标准”**项下第四条和第五条，如何保证受试者参与试验时可以达到此入组标准，请予以说明；
2. 试验方案第 20 页**“影像学检查”**项下“根据各临床研究中心临床治疗规范，术前和术中进行 DSA、CT、CTA、MRI 或者 MRA 检查”与方案的第 21 页**“试验流程图”**有差异，请予以修改；
3. 知情同意书第 1 页第三段中的“法定监护人”描述不准确，应修改为“法定代理人或被授权人”；
4. 知情同意书中的**“六.试验的资金来源、可能的利益冲突”**项下的内容描述过于笼统，建议进一步明确影像学检查（包括术前和术中）及所有项目的免费情况；
5. 知情同意书中的**“七.可能的受益”**项下的内容描述过于肯定，建议予以修改；
6. 知情同意书中的**“九.与试验相关伤害的治疗和经济补偿”**项下，建议将“如果您因使用试验支架取栓或者遵守正常试验程序时导致了经医疗鉴定的与试验支架有关的直接损害，诊断和治疗这类损害的医疗费用将由申办者根据法律、法规承担”修改为“如果您因使用试验支架取栓或者遵守正常试验程序时导致了经医疗鉴定的与试验支架有关的直接损害，由申办方承担医疗与赔偿责任”。

按审查意见修改后的文件，或对审查意见有不同观点的陈述，请提交“复审申请”，方案/知情同意书请注明新的版本号和版本日期，并以阴影和（或）下划线方式标注修改部分，报伦理委员会审查，经批准后执行。

伦理委员会	青岛大学附属医院医学伦理委员会
主任委员签字	[signature]
日期	2018 年 5 月 14 日

声明

本伦理委员会是相对独立的，委员会的职责、人员组成、操作程序及记录均遵循 ICH-GCP/中国 GCP 和中国相关法律和法规。所有出席委员均在委员会有效任职期间。特此声明。

青岛大学附属医院医学伦理委员会

2018.5

青岛大学附属医院医学伦理委员会

地　址：山东省青岛市市南区江苏路 16 号热电楼 3 楼　　邮编：266003

电话/传真：0532-82912611　　E-mail: qingyilunli@126.com

青岛大学附属医院医学伦理委员会（会议签到表）

会议日期　　2018年5月9日　　　　会议地点　　行政楼四楼会议室

姓名	性别	专业背景	职称	工作单位	签名
蒋光峰	男	耳鼻喉科	主任医师	青岛大学附属医院	
张国庆	男	医务管理	主任医师	青岛大学附属医院	
张晓春	女	肿瘤科	主任医师	青岛大学附属医院	
孙　伟	女	药学	主任药师	青岛大学附属医院	
于文成	男	内科	主任医师	青岛大学附属医院	请假
于　壮	男	肿瘤科	主任医师	青岛大学附属医院	
孔心涓	女	内科	主任医师	青岛大学附属医院	
牛　珉	男	医务管理	副主任药师	青岛大学附属医院	
邓晓红	女	教育学	高级教师	青岛市朝城路小学	请假
刘淑红	女	内科	主任医师	青岛大学附属医院	
孙立荣	女	儿科	主任医师	青岛大学附属医院	
孙　瑛	女	法学	法　官	青岛市市南区法院	请假
曲政海	男	儿科	主任医师	青岛大学附属医院	
冷　萍	女	药学	副主任药师	青岛大学附属医院	
吴力群	男	外科	主任医师	青岛大学附属医院	
李环廷	男	医务管理	副主任医师	青岛大学附属医院	
肖兴国	男	法学	律　师	山东瑞康律师事务所	
陶　昊	男	外科	副主任医师	青岛大学附属医院	请假
崔竹梅	女	妇产科	主任医师	青岛大学附属医院	
矫文捷	男	外科	主任医师	青岛大学附属医院	请假
阎胜利	男	内科	主任医师	青岛大学附属医院	
阎博民	男	医务管理	主任医师	青岛大学附属医院	
隋忠国	男	药学	主任药师	青岛大学附属医院	
蔡　霞	女	外科	主任医师	青岛大学附属医院	
魏丽丽	女	护理学	主任护师	青岛大学附属医院	请假

青岛大学附属医院

医学伦理委员会

会议审查决定表

项目	取栓器治疗急性缺血性卒中的前瞻性、多中心、单盲、随机对照临床试验	
投票意见	票数	会议决定
同意	票	☐ 同意
作必要的修正后同意	1 票	☐ 作必要的修正后同意
作必要的修正后重审	18 票	■ 作必要的修正后重审
不同意	票	☐ 不同意

因利益冲突退出 人

跟踪审查频率	______个月	截止日期	
是否调整跟踪审查频率	☐不变，☐改变	修正跟踪审查频率	______个月

青岛大学附属医院医学伦理委员会

伦理审查批件

批件号	器临伦审 QYFYEC 2018-004-01		
项目名称	取栓器治疗急性缺血性卒中的前瞻性、多中心、单盲、随机对照临床试验		
项目来源	申办方：微创神通医疗科技（上海）有限公司<br>CRO：方恩（天津）医药发展有限公司		
研究单位	神经内科专业		
主要研究者	张勇		
审查类别	初始审查后的复审	审查方式	会议审查
审查日期	2018 年 6 月 15 日	审查地点	行政楼四楼会议室
审查委员	蒋光峰、张国庆、张晓春、孙伟、于壮、孔心涓、牛珉、邓晓红、刘淑红、孙立荣、曲政海、冷萍、吴力群、李环廷、肖兴国、陶昊、崔竹梅、阎胜利、阎博民、隋忠国、蔡霞		
批准文件	1. 医疗器械产品技术要求<br>2. 临床试验方案 版本号：第 1.0 版 版本日期：2017 年 3 月 8 日<br>3. 研究者手册 版本号：第 1.0 版 版本日期：2017 年 3 月 8 日<br>4. 病例报告表 版本号：第 2.0 版 版本日期：2017 年 11 月 6 日<br>5. 原始病历 版本号：第 2.0 版 版本日期：2017 年 11 月 6 日<br>6. 知情同意书 版本号：第 2.0 版 版本日期：2018 年 5 月 11 日<br>7. 国家食品药品监督管理局上海医疗器械质量监督检验中心检验报告 报告编号：国医检（械）字 ZC2016 第 637 号；国医检（械）字 ZC2016 第 636 号<br>8. 取栓器自测报告 文件编号：050016B<br>9. 试验产品的动物试验报告<br>10. 主要研究者履历<br>11. 生命科学人体临床试验责任保险 保单号：92666573		

审查意见
根据卫计委《涉及人的生物医学研究伦理审查办法（2016）》、CFDA《药物临床试验质量管理规范（2003）》、《医疗器械临床试验质量管理规范（2016）》、WMA《赫尔辛基宣言》和CIOMS《人体生物医学研究国际道德指南》的伦理原则，经本伦理委员会审查，同意按所批准的临床研究方案、知情同意书开展本项研究。<br>请遵循 GCP 原则，遵循伦理委员会批准的方案开展临床研究，保护受试者的健康与权利。<br>研究开始前，请申请人完成临床试验注册。<br>研究过程中若变更主要研究者，对临床研究方案、知情同意书等的任何修改，请申请人提交修正案审查申请。<br>发生严重不良事件，请申请人及时提交严重不良事件报告。<br>请按照伦理委员会规定的年度/定期跟踪审查频率，申请人在截止日期前 1 个月提交研究进展报告；申办者应当向组长单位伦理委员会提交各中心研究进展的汇总报告；当出现任何可能显著影响试验进行或增加受试者危险的情况时，请申请人及时向伦理委员会提交书面报告。<br>研究纳入了不符合纳入标准或符合排除标准的受试者，符合中止试验规定而未让受试者退出研究，给予错误治疗或剂量，给予方案禁止的合并用药等没有遵从方案开展研究的情况；或可能对受试者的权益/健康以及研究的科学性造成不良影响等违背 GCP 原则的情况，请申办者/监查员/研究者提交违背方案报告。<br>申请人暂停或提前终止临床研究，请及时提交暂停/终止研究报告。<br>完成临床研究，请申请人提交结题报告。

年度/定期跟踪审查频率	请于 2019 年 6 月 21 日前 1 个月提交年度/定期跟踪审查
有效期	2018 年 6 月 22 日 - 2023 年 6 月 21 日
联系人与联系电话	张小蕾　　0532-82912611
主任委员签字	蒋光峰 [signature]
伦理委员会	青岛大学附属医院医学伦理委员会　　（盖章）
日期	2018 年 6 月 22 日

注：伦理批件批准 1 年内临床试验没有启动，则该批件自动失效。

青岛大学附属医院医学伦理委员会

声明

本伦理委员会是相对独立的，委员会的职责、人员组成、操作程序及记录均遵循 ICH-GCP/中国 GCP 和中国相关法律和法规。所有出席委员均在委员会有效任职期间。特此声明。

青岛大学附属医院医学伦理委员会

2018.6

青岛大学附属医院医学伦理委员会

地　址：山东省青岛市市南区江苏路 16 号热电楼 3 楼　　邮编：266003

电话/传真：0532-82912611　　E-mail: qingyilunli@126.com

青岛大学附属医院医学伦理委员会（会议签到表）

会议日期　　2018年6月15日　　　　会议地点　　行政楼四楼会议室

姓名	性别	专业背景	职称	工作单位	签名
蒋光峰	男	耳鼻喉科	主任医师	青岛大学附属医院	[illegible]
张国庆	男	医务管理	主任医师	青岛大学附属医院	[illegible]
张晓春	女	肿瘤科	主任医师	青岛大学附属医院	[illegible]
孙　伟	女	药学	主任药师	青岛大学附属医院	[illegible]
于文成	男	内科	主任医师	青岛大学附属医院	请假
于　壮	男	肿瘤科	主任医师	青岛大学附属医院	于壮
孔心涓	女	内科	主任医师	青岛大学附属医院	[illegible]
牛　珉	男	医务管理	副主任药师	青岛大学附属医院	[illegible]
邓晓红	女	教育学	高级教师	青岛市朝城路小学	邓晓红
刘淑红	女	内科	主任医师	青岛大学附属医院	[illegible]
孙立荣	女	儿科	主任医师	青岛大学附属医院	[illegible]
孙　瑛	女	法学	法　官	青岛市市南区法院	请假
曲政海	男	儿科	主任医师	青岛大学附属医院	[illegible]
冷　萍	女	药学	副主任药师	青岛大学附属医院	冷萍
吴力群	男	外科	主任医师	青岛大学附属医院	[illegible]
李环廷	男	医务管理	副主任医师	青岛大学附属医院	李环廷
肖兴国	男	法学	律　师	山东瑞康律师事务所	[illegible]
陶　昊	男	外科	副主任医师	青岛大学附属医院	陶昊
崔竹梅	女	妇产科	主任医师	青岛大学附属医院	[illegible]
矫文捷	男	外科	主任医师	青岛大学附属医院	请假
阎胜利	男	内科	主任医师	青岛大学附属医院	[illegible]
阎博民	男	医务管理	主任医师	青岛大学附属医院	[illegible]
隋忠国	男	药学	主任药师	青岛大学附属医院	[illegible]
蔡　霞	女	外科	主任医师	青岛大学附属医院	[illegible]
魏丽丽	女	护理学	主任护师	青岛大学附属医院	请假

青岛大学附属医院
医学伦理委员会

会议审查决定表

项目	取栓器治疗急性缺血性卒中的前瞻性、多中心、单盲、随机对照临床试验	
投票意见	票数	会议决定
同意	21 票	■ 同意
作必要的修正后同意	票	□ 作必要的修正后同意
作必要的修正后重审	票	□ 作必要的修正后重审
不同意	票	□ 不同意

因利益冲突退出 人

跟踪审查频率	12 个月	截止日期	2019 年 6 月 21 日
是否调整跟踪审查频率	□不变，□改变	修正跟踪审查频率	______个月

青岛大学附属医院医学伦理委员会

伦理审查批件

批件号	器临伦审 QYFYEC 2018-004-02		
项目名称	取栓器治疗急性缺血性卒中的前瞻性、多中心、单盲、随机对照临床试验		
项目来源	申办方：微创神通医疗科技（上海）有限公司<br>CRO：方恩（天津）医药发展有限公司		
研究单位	神经内科专业		
主要研究者	张勇		
审查类别	修正案审查		
审查委员 1	阎胜利	审查委员 2	牛珉
批准文件	1. 临床试验方案 版本号：第 2.0 版 版本日期：2018 年 8 月 8 日<br>2. 研究者手册 版本号：第 2.0 版 版本日期：2018 年 8 月 8 日<br>3. 病例报告表 版本号：第 3.0 版 版本日期：2018 年 8 月 8 日<br>4. 原始病历 版本号：第 3.0 版 版本日期：2018 年 8 月 8 日<br>5. 知情同意书 版本号：第 3.0 版 版本日期：2018 年 9 月 9 日<br>6. 取栓器使用说明书 版本号：Rev.2.0		

审查意见

根据卫计委《涉及人的生物医学研究伦理审查办法（2016）》、CFDA《药物临床试验质量管理规范（2003）》、《医疗器械临床试验质量管理规范（2016）》、WMA《赫尔辛基宣言》和 CIOMS《人体生物医学研究国际道德指南》的伦理原则，经本伦理委员会审查，同意按所批准的临床研究方案、知情同意书开展本项研究。

请遵循 GCP 原则、遵循伦理委员会批准的方案开展临床研究，保护受试者的健康与权利。

研究开始前，请申请人完成临床试验注册。

研究过程中若变更主要研究者，对临床研究方案、知情同意书等材料的任何修改，请申请人提交修正案审查申请。

发生严重不良事件，请申请人及时提交严重不良事件报告。

请按照伦理委员会规定的年度/定期跟踪审查频率，申请人在截止日期前 1 个月提交研究进展报告；申办者应当向组长单位伦理委员会提交各中心研究进展的汇总报告；当出现任何可能显著影响试验进行或增加受试者危险的情况时，请申请人及时向伦理委员会提交书面报告。

研究纳入了不符合纳入标准或符合排除标准的受试者，符合中止试验规定而未让受试者退出研究，给予错误治疗或剂量，给予方案禁止的合并用药等没有遵从方案开展研究的情况；或可能对受试者的权益/健康以及研究的科学性造成不良影响等违背 GCP 原则的情况，请申办者/监查员/研究者提交违背方案报告。

申请人暂停或提前终止临床研究，请及时提交暂停/终止研究报告。

完成临床研究，请申请人提交结题报告。

年度/定期跟踪审查频率	请于 2019 年 6 月 21 日前 1 个月提交年度/定期跟踪审查
有效期	2018 年 6 月 22 日 - 2023 年 6 月 21 日
联系人与联系电话	张小蕾　0532-82912611
主任委员签字	蒋光峰 [signature]
伦理委员会	青岛大学附属医院医学伦理委员会　（盖章）
日期	2018 年 11 月 5 日

声明

本伦理委员会是相对独立的，委员会的职责、人员组成、操作程序及记录均遵循 ICH-GCP/中国 GCP 和中国相关法律和法规。所有出席委员均在委员会有效任职期间。特此声明。

青岛大学附属医院医学伦理委员会

2018.11

青岛大学附属医院医学伦理委员会

地　址：山东省青岛市市南区江苏路 16 号热电楼 3 楼　　邮编：266003

电话/传真：0532-82912611　　E-mail: qingyilunli@126.com
