# Supplementary material for: Endovascular treatment of acute ischemic stroke with a fully radiopaque retriever: A randomized controlled trial
Source: Front Neurol. 2022 Dec 14;13:962987. doi: 10.3389/fneur.2022.962987 (PMC9796564; doi:10.3389/fneur.2022.962987)
Supplement: Supplementary file 1 [file Data_Sheet_1.zip › 14 ┴─│╟.pdf]

聊城市人民医院
药物临床试验伦理委员会审查批件（器械）

（2018001）号

试验项目名　称	取栓器治疗急性缺血性卒中的前瞻性、多中心、单盲、随机对照临床试验		
产品名称	取栓器	检验类别	注册检验
型号规格	AIS4025<br>AIS6030	会议类别	会议初审
产品编号/批号	02010001、02010002、02010003、02020001、02020002、02020003	检测报告	国医检（械）字 ZC2016 第 636 号、第 637 号
承担科室	神经外科	临床试验负责人	王继跃
申办者名称/地址	微创神通医疗技术（上海）有限公司<br>合同研究组织：方恩（天津）医药发展有限公司		
审查内容	研究者资格、人员配备及设备条件：　符合要求☑　不符合要求☐<br>试验方案（编号和版本号）：2016-GATOR-01-A　V1.0　日期：2017-03-08　适当☑　不适当☐<br>知情同意书(编号和版本号)：2016-GATOR-01-E　V1.0　日期：2017-03-08　适当☑　不适当☐<br>研究者手册(编号和版本号)：2016-GATOR-01-G　V1.0　日期：2017-03-08　适当☑　不适当☐<br>病例报告表(编号和版本号)：2016-GATOR-01-F　V2.0　日期：2017-11-06　适当☑　不适当☐<br>招募说明：　不适用☐　适当☑　不适当☐<br>其他：申办方/CRO 资质证明、CRO 委托书、保险凭证、产品自测报告、产品注册检验报告、取栓器说明书、对照产品说明书、医疗器械产品技术要求、试验用医疗器械的研制符合适用的医疗器械质量管理体系相关要求的声明、取栓器出厂检验报告、动物实验报告、器械销毁委托书、器械运输及储存温湿度说明、组长单位的临床试验批件、原始病历（V2.0,2017-11-06）。		
出席人数	应到 15 名	实到 12 名	
投票结果	同意 12 票	不同意 0 票	作必要修正后同意 0 票
	作必要修正后重审 0 票	终止或暂停已批准的试验 0 票	回避 0 票

注：试验过程中如果发生严重 SAE，请及时报告伦理委员会；如果试验过程超过一年，伦理委员会及时对项目进行年度跟踪审查。伦理委员会地址：聊城市东昌西路 67 号，联系电话 0635-8276111

审查意见	试验方案适当合理，知情同意书通俗易懂，受试者受试者的隐私保护、补偿和知情权得到有效的保障<br>伦理审查意见：同意开展临床试验。<br><br>主任（副主任）委员（签字）：王大伟<br>2018 年 4 月 2 日

委　员	性 别	所 属 单 位	专 业	职务/职称	签 名
王大伟	男	聊城市人民医院	骨 外	主任医师	王大伟
胥庆华	男	聊城市人民医院	保健内	主任医师	[illegible]
路庆良	男	聊城市人民医院	伦理学	党办主任	路庆良
马龙乐	男	聊城市人民医院	心血管病	主任医师	
张连荣	女	聊城市人民医院	护理学	主任护师	张连荣
杨巧芝	女	聊城市人民医院	儿 科	主任医师	杨巧芝
赵岐刚	男	聊城市人民医院	医学检验	主任技师	赵岐刚
陈建中	男	聊城市人民医院	药 学	副主任药师	陈建中
李雪莉	女	聊城市人民医院	神经内	主任医师	李雪莉
谷方里	男	聊城市人民医院	中医科	主任医师	
张　伟	男	聊城市人民医院	两腺外	副主任医师	张伟
李　铸	男	聊城市人民医院	肝胆外	副主任医师	李铸
闫光瑜	男	山东鲁衡律师事务所	法 学	律 师	闫光瑜
姜　琳	女	聊城市技师学院	教 师	高级实习指导教师	姜琳
臧利敏	女	聊城市文联	文学创作	三级	

聊城市人民医院药物临床试验伦理委员会

声明：参加审批的伦理委员会独立于医院和申办者，并且本伦理委员会严格按照 ICH-GCP、中国 GCP 及相关法规组成和工作。

聊城市人民医院
药物临床试验伦理委员会审查批件（器械）

（2018011）号

<table>
<tr><td>试验项目名　称</td><td colspan="3">取栓器治疗急性缺血性卒中的前瞻性、多中心、单盲、随机对照临床试验</td></tr>
<tr><td>产品名称</td><td>取栓器</td><td>检验类别</td><td>注册检验</td></tr>
<tr><td>型号规格</td><td>AIS4025
AIS6030</td><td>会议类别</td><td>会议重审</td></tr>
<tr><td>产品编号/批号</td><td>02010004、02010005、02010006、02010007、02020005、02020006、0202007</td><td>检测报告</td><td>国医检（械）字 ZC2016 第 636 号、第 637 号</td></tr>
<tr><td>承担科室</td><td>神经外科</td><td>临床试验负责人</td><td>王继跃</td></tr>
<tr><td>申办者名称/地址</td><td colspan="3">微创神通医疗技术（上海）有限公司
合同研究组织：方恩（天津）医药发展有限公司</td></tr>
<tr><td>审查内容</td><td colspan="3">研究者资格、人员配备及设备条件：　符合要求☑　不符合要求☐
试验方案（编号和版本号）：2016-GATOR-01-A　V2.0
日期：2018-08-08　适当☑　不适当☐
知情同意书(编号和版本号)：2016-GATOR-01-8E　V3.0
日期：2018-09-09　适当☑　不适当☐
研究者手册(编号和版本号)：2016-GATOR-01-G　V2.0
日期：2018-08-08　适当☑　不适当☐
病例报告表(编号和版本号)：2016-GATOR-01-F　V3.0
日期：2018-08-08　适当☑　不适当☐
原始病历(编号和版本号）2016-GATOR-01-I　V3.0
日期：2018-08-08　适当☑　不适当☐
其他：取栓器说明书(版本号：2.0，文件编号 A-T0006-002)、SAE 豁免说明书-20180914，长海医院-方案修正等快审批件-20180925。</td></tr>
<tr><td>出席人数</td><td>应到 15 名</td><td colspan="2">实到 11 名</td></tr>
<tr><td rowspan="2">投票结果</td><td>同意 11 票</td><td>不同意 0 票</td><td>作必要修正后同意 0 票</td></tr>
<tr><td>作必要修正后重审 0 票</td><td>终止或暂停已批准的试验 0 票</td><td>回避 0 票</td></tr>
</table>

注：试验过程中如果发生严重 SAE，请及时报告伦理委员会；如果试验过程超过一年，伦理委员会及时对项目进行年度跟踪审查。伦理委员会地址：聊城市东昌西路 67 号，联系电话 0635-8276111

审查意见	经审查，试验方案适当可行，知情同意书通俗易懂，能保障受试者合法权益。<br>同意开展临床试验。<br><br>主任（副主任）委员（签字）：王大伟　　2018 年 12 月 4 日

委　员	性 别	所 属 单 位	专 业	职务/职称	签　名
王大伟	男	聊城市人民医院	骨 外	主任医师	王大伟
胥庆华	男	聊城市人民医院	保健内	主任医师	
路庆良	男	聊城市人民医院	伦理学	党办主任	路庆良
马龙乐	男	聊城市人民医院	心血管病	主任医师	
张连荣	女	聊城市人民医院	护理学	主任护师	张连荣
杨巧芝	女	聊城市人民医院	儿 科	主任医师	
赵岐刚	男	聊城市人民医院	医学检验	主任技师	赵岐刚
陈建中	男	聊城市人民医院	药 学	副主任药师	陈建中
李雪莉	女	聊城市人民医院	神经内	主任医师	李雪莉
谷万里	男	聊城市人民医院	中医科	主任医师	谷万里
张　伟	男	聊城市人民医院	两腺外	副主任医师	张伟
李　铸	男	聊城市人民医院	肝胆外	副主任医师	李铸
闫光瑜	男	山东鲁衡律师事务所	法 学	律 师	闫光瑜
姜　琳	女	聊城市技师学院	教 师	高级实习指导教师	姜琳
臧利敏	女	聊城市文联	文学创作	三级	

聊城市人民医院药物临床试验伦理委员会

声明：参加审批的伦理委员会独立于医院和申办者，并且本伦理委员会严格按照 ICF/GCP、中国 GCP 及相关法规组成和工作。
