# Supplementary material for: Endovascular treatment of acute ischemic stroke with a fully radiopaque retriever: A randomized controlled trial
Source: Front Neurol. 2022 Dec 14;13:962987. doi: 10.3389/fneur.2022.962987 (PMC9796564; doi:10.3389/fneur.2022.962987)
Supplement: Supplementary file 1 [file Data_Sheet_1.zip › 15 ╫═▓⌐.pdf]

淄博市中心医院药物临床试验伦理委员会
伦理审查批件

（2018）伦审第（005）号

项目名称	取栓器治疗急性缺血性卒中的前瞻性、多中心、单盲、随机对照临床试验		
申办单位	微创神通医疗科技（上海）有限公司		
研究单位	淄博市中心医院		
研究者/科室	翟乃池/神经外科	审查方式	☒会议审查 ☐快速审查
审查时间	2018-01-26	审查地点	淄博市中心医院北病房楼学术报告厅
审查文件	试验方案（版本号 V1.0，版本日期 2017—03-08）<br>研究者手册（版本号 V1.0，版本日期 2017-03-08）<br>知情同意书（版本号 V1.0，版本日期 2017-03-08）<br>受试者招募说明（日期 2018-01-03）		
审查委员	见委员签到表		
审查意见	1. 审查决定：<br>☒同意 ☐修正后同意 ☐修正后重审<br>☐不同意 ☐暂停或终止研究<br>2. 审查频率：<br>☐3 个月 ☐6 个月 ☒12 个月<br>3. 批件效期：1 年		
备注	1. 本研究应当在伦理委员会同意之日起 1 年内实施，逾期未实施的，本批件自行作废。<br>2. 请遵循 GCP 的原则及本伦理委员会批准的临床研究方案开展临床研究，保护受试者的权益。<br>3. 研究过程中发生主要研究者变更，临床试验方案、知情同意书的修改，应提交修正案审查申请。<br>4. 按规定的年度/定期跟踪审查频率，在跟踪审查到期前 1 个月递交研究进展报告。<br>5. 试验过程中若发生违背试验方案或 GCP 原则，及时提交违背方案报告。<br>6. 申请人暂停或提前终止临床研究，及时提交暂停/终止研究报告。<br>7. 试验完成，应递交结题报告。		

主任/副主任委员签字：[signature]

日 期：2018.2.2

淄博市中心医院药物临床试验伦理委员会 （盖章）

本伦理委员会地址：山东省淄博市张店区共青团西路 54 号；邮编：255036；电话：0533-2360337.

会议签到表

伦理委员会名称	淄博市中心医院药物临床试验伦理委员会
会议日期	2018-01-26
会议地点	北病房楼学术报告厅

姓名	性别	专业	职务	工作单位	签到
崔德杰	男	行政	主任委员	淄博市中心医院	崔德杰
鲍秀丽	女	行政	副主任委员	淄博市中心医院	鲍秀丽
司继刚	男	药学	委员	淄博市中心医院	司继刚
刘　红	女	护理	委员	淄博市中心医院	/
陈佑生	男	呼吸	委员	淄博市中心医院	陈佑生
梁文龙	男	内分泌	委员	淄博市中心医院	梁文龙
刘艳华	女	护理	委员	淄博市中心医院	刘艳华
崔　庆	男	普外	委员	淄博市中心医院	崔庆
周忠民	男	肾内	委员	淄博市中心医院	周忠民
张　波	男	律师	委员	山东理工大学	张波
陈　宏	女	教育	委员	淄博市文化局	/

淄博市中心医院药物临床试验伦理委员会
伦理快速审查批件

（2018）伦审第（005）号-2

项目名称	取栓器治疗急性缺血性卒中的前瞻性、多中心、单盲、随机对照临床试验		
申办单位	微创神通医疗科技（上海）有限公司		
研究单位	淄博市中心医院		
研究者/科室	翟乃池/神经外科	审查方式	☐会议审查 ☑快速审查
审查时间	NA	审查地点	NA
审查文件	见附件		
审查委员	崔 庆 司继刚		
审查意见	经伦理委员会审查，同意按修改后的方案及文件遵循 GCP 的原则进行临床试验。<br>跟踪审查频率：☒不变 ☐修正为_____个月		
备注	1. 本研究应当在伦理委员会同意之日起 1 年内实施，逾期未实施的，本批件自行作废。<br>2. 请遵循 GCP 的原则及本伦理委员会批准的临床研究方案开展临床研究，保护受试者的权益。<br>3. 研究过程中发生主要研究者变更，临床试验方案、知情同意书的修改，应提交修正案审查申请。<br>4. 按规定的年度/定期跟踪审查频率，在跟踪审查到期前 1 个月递交研究进展报告。<br>5. 试验过程中若发生违背试验方案或 GCP 原则，及时提交违背方案报告。<br>6. 申请人暂停或提前终止临床研究，及时提交暂停/终止研究报告。<br>7. 试验完成，应递交结题报告。		

主任/副主任委员签字：[illegible]

日 期：2018.10.26

淄博市中心医院药物临床试验伦理委员会（盖章）

本伦理委员会地址：山东省淄博市张店区共青团西路 54 号；邮编：255036；电话：0533-2360337.

淄博市中心医院药物临床试验伦理委员会
伦理审查批件附件

（2018）伦审第（005）号-2

项目名称：

取栓器治疗急性缺血性卒中的前瞻性、多中心、单盲、随机对照临床试验

审查资料：

1.临床研究方案 版本号：2.0，版本日期：2018-08-08

2.知情同意书 版本号：2.0，版本日期：2018-08-08

3.研究者手册 版本号：2.0，版本日期 2018-08-08

4.病例报告表 版本号：3.0，版本日期 2018-08-08

5.原始病历 版本号：3.0，版本日期 2018-08-08

6.取栓器说明书 版本号：2.0，文件编号：A-T0006-002

7.SAE 豁免说明-20180914

8.长海医院-方案修正等快审批件-20180925
