# Supplementary material for: Endovascular treatment of acute ischemic stroke with a fully radiopaque retriever: A randomized controlled trial
Source: Front Neurol. 2022 Dec 14;13:962987. doi: 10.3389/fneur.2022.962987 (PMC9796564; doi:10.3389/fneur.2022.962987)
Supplement: Supplementary file 2 [file Data_Sheet_2.zip › 17 ╠╞╢╝╥╜╘║.pdf]

第四军医大学唐都医院医学伦理委员会伦理审查批件

唐都医院医学伦理委员会批件号：第 201809-07 号

试验项目名称	取栓器治疗急性缺血性卒中的前瞻性、多中心、 单盲、 随机对照临床试验		
注册分类	3 类医疗器械	审批资料	见附件
申请专业科室	神经外科	申办单位	微创神通医疗科技（上海）有限公司
专业组负责人	高国栋 主任	主要研究者	赵振伟 主任
审查类别	初始审查	审查日期	2018.9.18
审查方式	■会议审查 ☐紧急会议审查 ☐快速审查	审查地点	肿瘤科学习室
委员出席人数	应到委员 9 人，实到委员 8 人，其中投票 8 人，回避 0 人，缺席 1 人		
审查结论	☐同意 ☑作必要修正后同意 ☐作必要修正后重审 ☐不同意 ☐终止或暂停已批准的研究		
年度/定期跟踪审查	☑是 ☐否	跟踪审查频率	☐3 个月 ☐6 个月 ☑12 个月
审批意见	（包括对研究者资质、人员配备及条件、试验方案、知情同意书及受试者权益保护等的评价）<br>本伦理委员会按照中国 GCP、ICH-GCP 及有关法规组成并开展工作，对本项目的审查意见如下：<br>1. 主要研究者及项目组成员具有丰富的临床经验，具有作为研究者进行研究的资质，能够保证项目的研究时间和条件。<br>2. 研究目的明确，设计合理，科学性强。<br>3. 知情同意书存在问题如下：①伦理委员会联系人及电话有误；②签字页中“或受试者为未成年人的”，本研究入选标准不含未成年人，请去掉。<br>经讨论，作必要修正后同意该项目的临床研究。<br>附：伦理审查文件清单。<br>主任委员签名：[illegible]<br>伦理委员会（盖章）<br>时　间：2018 年 9 月 18 日		

备注：

请遵循 GCP 原则、遵循伦理委员会批准的方案开展临床研究，保护受试者的健康与权益。

研究过程中，若变更主要研究者，对临床研究方案、知情同意书、招募材料等的任何修改，请申请人提交修正案审查申请。

发生严重不良事件，请申请人及时提交严重不良事件报告。

请按照伦理委员会规定的年度/定期跟踪审查频率，申请人在截止日期前 1 个月提交研究进展报告；申办者应向组长单位伦理委员会提交各中心研究进展的汇总报告；当出现可能显著影响试验进行或增加受试者危险情况时，请申请人及时向伦理委员会递交书面报告。

研究纳入了不符合纳入标准或符合排除标准的受试者，符合中止试验规定而未让受试者退出研究，给予错误治疗或剂量，给予方案禁止的合并用药等没有遵从方案开展研究的情况；或可能对受试者的权益/健康以及研究的科学性造成不良影响等违背 GCP 原则的情况，请申办者/监查员/研究者提交违背方案报告。

申请人暂停或提前终止临床研究，请及时提交暂停/终止研究报告。

完成临床研究，请申请人提交结题报告供伦理委员会进行审查。

声明：本伦理委员会的职责、人员组成、操作程序及记录遵循 ICH-GCP、中国 GCP 及中国的相关法律和法规。本伦理委员会所有出席的委员均在有效任职期间，将对所审阅的临床研究资料以及伦理委员会会议讨论的结果和相关的内容保密，且与本研究项目无利益冲突。本批件有效期 1 年，逾期未实施者则自行废止。

联系地址：西安市灞桥区新寺路 569 号第四军医大学唐都医院临床教学楼 304 室；邮编：710038；邮箱：tangduec@126.com；
联系人：李诗草；联系电话/传真：029-84777631

附：伦理审查文件清单【取栓器治疗急性缺血性卒中的前瞻性、多中心、单盲、随机对照临床试验】

1. 产品技术要求；
2. 自检报告；
3. 产品合格检验报告（型号：AIS4025，AIS6030），以及对照产品检验报告；
4. 动物实验报告；
5. 研究者手册（V1.0　20170308）；
6. 申办者资质证明文件；
7. 方案（V1.0　20170308）；
8. 知情同意书（V2.0　20180905）；
9. 病例报告表(V2.0　20171106)；
10. 主要研究者简历及GCP培训证书复印件；
11. 其他单位对该临床试验的伦理审查批件；
12. 申办者对CRO的委托函及签字授权书；
13. 关于无国家食品药品监督管理局批件的说明；
14. 肝功能时间窗豁免说明；
15. 受试者招募说明；
16. 保险证明；
17. 无患者日记卡说明；
18. 产品说明书；
19. 临床试验器械销毁委托书；
20. 器械运输及储存温湿度说明；
21. 对照产品说明；
22. 合并用药采集时间说明；
23. SICH时间窗豁免说明；
24. 医疗器械临床试验备案表。

第四军医大学唐都医院医学伦理委员会签到表

会议地点：肿瘤科学习室				会议时间：2018.9.18	
伦理委员会职务	姓名	性别	工作单位	专业背景/职称	是否出席及签名
主任委员	张贺龙	男	唐都医院肿瘤科	医学/主任医师	张贺龙
副主任委员	郭照江	男	第四军医大学社科部	医学伦理学/教授	郭照江
委员	张　琰	女	唐都医院药剂科	药学/主任药师	[illegible]
委员	郝春秋	男	唐都医院感染科	医学/主任医师	郝春秋
委员	李学拥	男	唐都医院烧伤整形科	医学/主任医师	李学拥
委员	李志立	男	唐都医院心内科	医学/副主任医师	请假
委员	刘琳娜	女	唐都医院药剂科	药理学/副主任药师	刘琳娜
委员	郑本性	男	西安市灞桥区法院	法律/四级高级法官	郑本性
委员	王清荣	女	西北核技术研究所	无线电/高级技工	王清荣
秘书	李诗草	女	唐都医院药剂科	药学/药师	李诗草

第四军医大学唐都医院医学伦理委员会伦理审查批件

唐都医院医学伦理委员会批件号：第 201811-23 号

试验项目名称	取栓器治疗急性缺血性卒中的前瞻性、多中心、单盲、随机对照临床试验		
注册分类	第 3 类医疗器械	审批资料	见附件
申请专业科室	神经外科	申办单位	微创神通医疗科技（上海）有限公司
专业组负责人	高国栋 主任	主要研究者	赵振伟 主任
审查类别	修正案审查	审查日期	2018.11.07
审查方式	■会议审查 □紧急会议审查 □快速审查	审查地点	肿瘤科学习室
委员出席人数	应到委员 9 人，实到委员 8人，其中投票 8人，回避 0人，缺席 1 人		
审查结论	☑同意 □作必要修正后同意 □作必要修正后重审 □不同意 □终止或暂停已批准的研究		
年度/定期跟踪审查	☑是 □否	跟踪审查频率	□3 个月 □6 个月 ☑12 个月
审批意见	(包括对研究者资质、人员配备及条件、试验方案、知情同意书及受试者权益保护等的评价)<br>本伦理委员会按照中国 GCP、ICH-GCP 及有关法规组成并开展工作，对本项目的审查意见如下：<br>1. 本伦理委员会对该项目原批件（批件号：第 201809-07 号）中的意见依然有效。<br>2. 同意申办方对研究方案、知情同意书、原始病历、使用说明书等文件的修订。<br>主任委员签名：[illegible]<br>伦理委员会（盖章）<br>时　间：2018年11月7日		

备注：

请遵循 GCP 原则、遵循伦理委员会批准的方案开展临床研究，保护受试者的健康与权益。

研究过程中，若变更主要研究者，对临床研究方案、知情同意书、招募材料等的任何修改，请申请人提交修正案审查申请。

发生严重不良事件，请申请人及时提交严重不良事件报告。

请按照伦理委员会规定的年度/定期跟踪审查频率，申请人在截止日期前 1 个月提交研究进展报告；申办者应向组长单位伦理委员会提交各中心研究进展的汇总报告；当出现可能显著影响试验进行或增加受试者危险情况时，请申请人及时向伦理委员会递交书面报告。

研究纳入了不符合纳入标准或符合排除标准的受试者，符合中止试验规定而未让受试者退出研究，给予错误治疗或剂量，给予方案禁止的合并用药等没有遵从方案开展研究的情况；或可能对受试者的权益/健康以及研究的科学性造成不良影响等违背 GCP 原则的情况，请申办者/监查员/研究者提交违背方案报告。

申请人暂停或提前终止临床研究，请及时提交暂停/终止研究报告。

完成临床研究，请申请人提交结题报告供伦理委员会进行审查。

声明：本伦理委员会的职责、人员组成、操作程序及记录遵循 ICH-GCP、中国 GCP 及中国的相关法律和法规。本伦理委员会所有出席的委员均在有效任职期间，将对所审阅的临床研究资料以及伦理委员会会议讨论的结果和相关的内容保密，且与本研究项目无利益冲突。本批件有效期 1 年，逾期未实施者则自行废止。

联系地址：西安市灞桥区新寺路 569 号第四军医大学唐都医院临床教学楼 304 室；邮编：710038；邮箱：tangduec@126.com；
联系人：李诗草；联系电话/传真：029-84777631

附：伦理审查文件清单【取栓器治疗急性缺血性卒中的前瞻性、多中心、单盲、随机对照临床试验】

1. 临床试验方案 V2.0 修订说明；
2. 临床试验方案（V2.0 20180808）；
3. 临床试验方案 V2.0 痕迹版；
4. 临床试验方案 V2.0 修订列表；
5. 研究者手册（V2.0 20180808）；
6. 研究者手册 V2.0 痕迹版；
7. 研究者手册 V2.0 修订列表；
8. 知情同意书（V3.0 20181008）；
9. 知情同意书 V3.0 痕迹版；
10. 知情同意书 V3.0 修订列表；
11. 病例报告表（V3.0 20180808）；
12. 病例报告表 V3.0 痕迹版；
13. 病例报告表修订列表；
14. 原始病历（V3.0 20180808）；
15. 原始病历 V3.0 痕迹版；
16. 原始病历修订列表；
17. 取栓器使用说明书（文件编号：A-T0006-002 Rev.2.0）；
18. 取栓器使用说明书痕迹版；
19. 取栓器使用说明书修订列表

第四军医大学唐都医院医学伦理委员会签到表

会议地点： 肿瘤科学习室				会议时间：2018. 11. 07	
伦理委员会职务	姓名	性别	工作单位	专业背景/职称	是否出席及签名
主任委员	张贺龙	男	唐都医院肿瘤科	医学/主任医师	张贺龙
副主任委员	郭照江	男	第四军医大学社科部	医学伦理学/教授	郭照江
委员	张　琰	女	唐都医院药剂科	药学/主任药师	张琰
委员	郝春秋	男	唐都医院感染科	医学/主任医师	请假
委员	李学拥	男	唐都医院烧伤整形科	医学/主任医师	李学拥
委员	宿长军	男	唐都医院神经内科	医学/主任医师	宿长军
委员	刘琳娜	女	唐都医院药剂科	药理学/副主任药师	刘琳娜
委员	郑本性	男	西安市灞桥区法院	法律/四级高级法官	郑本性
委员	王清荣	女	西北核技术研究所	无线电/高级技工	王清荣
秘书	李诗草	女	唐都医院药剂科	药学/药师	李诗草
