# Supplementary material for: Endovascular treatment of acute ischemic stroke with a fully radiopaque retriever: A randomized controlled trial
Source: Front Neurol. 2022 Dec 14;13:962987. doi: 10.3389/fneur.2022.962987 (PMC9796564; doi:10.3389/fneur.2022.962987)
Supplement: Supplementary file 2 [file Data_Sheet_2.zip › 18 ║╝╓▌╩╨╥╗ .pdf]

杭州市第一人民医院伦理委员会临床试验审查批件

批件号:【2018】医伦审第（049）号-01

项目名称：取栓器治疗急性缺血性卒中的前瞻性、多中心、单盲、随机对照临床试验		
申办者：微创神通医疗科技（上海）有限公司		
CRO： 方恩（天津）医药发展有限公司		
临床试验科室：神经内科	主要研究者：殷聪国	申请事项：初始审查(器械III类)
临床批件：/	药品类别：/	试验分期：/
审查方式： 会议审查	会议时间：2018.10.30	会议地点：7号楼7楼远程会诊中心
伦理委员会联系人： 陆蕴	联系电话：0571－56007507	
审查文件： 见附件		

投票结果：伦理委员会对上述文件进行了认真的审查和讨论，并进行了投票表决，应到人数：9人，实到人数：8人，其中：投票人数：8人，回避：0人，弃权：0人。

同意	作必要修正后同意	作必要修正后重审	终止或暂停已批准试验	不同意
8票	0票	0 票	0 票	0 票

审查意见：

根据卫计委《涉及人的生物医学研究伦理审查办法（2016）》，CFDA《药物临床试验伦理审查工作指导原则（2010）》，《药物临床试验质量管理规范（2003）》，《医疗器械临床试验质量管理规范（2016）》，WMA《赫尔辛基宣言》和 CIOMS《人体生物医学研究国际道德指南》的伦理原则，经伦理委员会审查，同意按所批准的临床方案（V2.0，2018.08.08）、知情同意书（V2.0，2018.08.08）等开展本项研究。

年度/定期跟踪审查频率：1 年

本批件有效期 1 年(自批准之日起)，如试验逾期未实施即自行废止。

杭州市第一人民医院伦理委员会（盖章）

（印章：杭州市第一人民医院伦理委员会）

主任委员签名：[签名] 日期：2018.10.31

注意事项：

请遵循 GCP 原则，遵循伦理委员会批准的方案开展临床研究，保护受试者的健康和权利。

研究过程中若变更主要研究者、对临床方案、知情同意书、招募材料等的任何修改，请申请人提交修正案审查申请。

发生严重不良事件，请申请人及时提交严重不良事件报告。

请按照伦理委员会规定的年度/定期跟踪审查频率，申请人在截止日期前 1 个月提交研究进展报告。

出现方案违背，请申请人及时提交违背方案报告。

申请人暂停/提前终止临床研究，请及时提交暂停/终止研究报告。

完成临床研究，请申请人提交结题报告。

地址：杭州市上城区浣纱路 261 号　　邮编：310006

附件：

项目名称：取栓器治疗急性缺血性卒中的前瞻性、多中心、单盲、随机对照临床试验

1、医疗器械临床试验备案表

2、研究者手册（V2.0，2018.08.08）

3、临床试验方案（V2.0，2018.08.08）

4、研究病历（V3.0，2018.08.08）

5、病例报告表（V3.0，2018.08.08）

6、知情同意书（V2.0，2018.08.08

7、招募说明

8、组长单位伦理批件（上海长海医院）

9、其他参与单位和主要研究者名单

10、主要研究者及研究者履历

11、医疗器械产品技术要求

12、自测报告

13、产品型式试验合格报告（上海医疗器械质量监督检验中心）

14、试验用医疗器械研制符合适用的医疗器械质量管理体系相关要求的声明

15、临床试验机构的设施和条件能够满足试验的综述

16、动物实验报告

17、产品说明书

18、保险单

19、器械生产许可证复印件

20、器械生产企业法人营业执照（副本）复印件

21、申办方保证所提供资料真实性的声明

22、CRO 公司委托书及资质证明

23、SMO 公司委托书及资质证明

24、其他：对照产品合格证

对照产品出厂检验报告及采购说明

对照产品医疗器械注册证

销售授权书，进口货物报告单

杭州市第一人民医院
伦理委员会

杭州市第一人民医院伦理委员会会议签到
及保密协议、利益冲突申明

杭州市第一人民医院严格按照 GCP 及相关法规组成伦理委员会，审查临床试验项目。为保证临床试验伦理审查的公平、公正，作为伦理委员会成员，保护受试者的安全和权益，对所审查的材料及伦理委员会会议的内容保密。并保证在本人任期中，若与该项目存在利益冲突，则在伦理审查前向伦理委员会主任说明，并从会议的决定程序中退出，绝不干扰其他伦理委员会成员的决定。

姓名	性别	职称	专业	单位	签名
黄进宇	男	主任医师	心血管内科	杭州市第一人民医院	[illegible]
张　楚	男	主任医师	内分泌	杭州市第一人民医院	请假
朱　瑾	男	主任医师	耳鼻咽喉科	杭州市第一人民医院	[illegible]
封光华	男	主任医师	普外科	杭州市第一人民医院	[illegible]
王　鸣	男	主任医师	肾内科	杭州市第一人民医院	[illegible]
俞小虹	女	主任医师	皮肤性病科	杭州市第一人民医院	[illegible]
严伟	女	主任药师	药学	杭州市第一人民医院	[illegible]
赵丽华	女	律师	法律	浙江天卫律师事务所	[illegible]
李军	女	/	退休工人	东坡路社区	[illegible]

杭州市第一人民医院 伦理委员会

2018年10月30日
