# Supplementary material for: Endovascular treatment of acute ischemic stroke with a fully radiopaque retriever: A randomized controlled trial
Source: Front Neurol. 2022 Dec 14;13:962987. doi: 10.3389/fneur.2022.962987 (PMC9796564; doi:10.3389/fneur.2022.962987)
Supplement: Supplementary file 2 [file Data_Sheet_2.zip › 19 ╔╜╬≈╨─╤¬╣▄.pdf]

山西省心血管病医院药物临床试验伦理委员会

会议审查批件

审查受理编号：2018015　　审查时间：2018 年 9 月 28 日

签发日期：2018 年 10 月 12 日

<table>
<tr><td>项目名称</td><td colspan="5">取栓器治疗急性缺血性卒中的前瞻性、多中心、单盲、随机对照临床试验</td></tr>
<tr><td>项目来源</td><td colspan="5">微创神通医疗科技(上海)有限公司</td></tr>
<tr><td>研究单位/科室</td><td colspan="5">山西省心血管病医院神经外科</td></tr>
<tr><td>主要研究者</td><td colspan="5">蒯东</td></tr>
<tr><td rowspan="3">审核内容</td><td>研究方案</td><td colspan="4">版本号：V1.0　版本日期：20170308</td></tr>
<tr><td>知情同意书</td><td colspan="4">版本号：V1.0　版本日期：20170308</td></tr>
<tr><td>其他文件</td><td colspan="4">受试者招募广告
病例报告表（版本号 V2.0 版本日期 20171106）
研究者手册（版本号 V1.0 版本日期 20170308）
主要研究者履历及研究人员名单、职责分工等</td></tr>
<tr><td colspan="6">投票结果</td></tr>
<tr><td>同意</td><td>修改后同意</td><td>修改后重审</td><td>暂停或终止</td><td colspan="2">不同意</td></tr>
<tr><td>6 票</td><td>5 票</td><td>0 票</td><td>0 票</td><td colspan="2">0 票</td></tr>
<tr><td>审核意见</td><td colspan="5">伦理委员会按照国家相关法规要求对该临床试验的研究方案及知情同意书等材料进行审阅和充分讨论，最终讨论决定为同意该项目在我院实施。</td></tr>
<tr><td colspan="6">主任委员签字：[signature]　　伦理委员会（盖章）
日期：2018.10.12</td></tr>
</table>

声明：本伦理委员会组成和工作程序符合 GCP 原则及国家相关法律法规。自批 件签署日期起 1 年内开始临床研究，本批件有效。

备注：

1.根据卫生部《涉及人的生物医学研究伦理审查办法》(2016)、SFDA《药物临床试验质量管理规范(2003)》、《医疗器械临床试验规定(2004)》、WMA《赫尔辛基宣言》和 CIOMS《人体生物医学研究国际道德指南》的伦理原则， 经本伦理委员会审查，同意按所批准的临床研究方案、知情同意书、招募材料 开展本项研究。

2.请遵循 GCP 原则、遵循伦理委员会批准的方案开展临床研究，保护受试者的健康与权利。

3.研究开始前，请申请人完成临床试验注册。

4.研究过程中若变更主要研究者，对临床研究方案、知情同意书、招募材 料等的任何修改，请申请人提交修正案审查申请。

5.发生严重不良事件，请申请人及时提交严重不良事件报告。

6.请按照伦理委员会规定的年度/定期跟踪审查频率，申请人在截止日期前一个月提交研究进展报告；申办者应当向组长单位伦理委员会提交各中心研究进展的汇总报告；当出现任何可能显著影响试验进行、或增加受试者危险的情况时，请申请人及时向伦理委员会提交书面报告。

7.研究纳入了不符合纳入标准或符合排除标准的受试者，符合中止试验规定而未让受试者退出研究，给予错误治疗或剂量，给予方案禁止的合并用药等没有遵从方案开展研究的情况；或可能对受试者的权益/健康以及研究的科学性造成不良影响等违背 GCP 原则的情况，请申办者/监察员/研究者提交违背方案 报告。

8.申请人暂停或提前终止临床研究，请及时提交暂停/终止研究报告。

9.完成临床研究，请申请人提交研究完成报告。

10.伦理委员会每年年底将会对试验研究进行跟踪审查。

11.此批件的有效期为自签发之日起 1 年。

联系人：张鑫

联系电话：0351-5661182

伦理委员会委员签到表

日期：2018 年 9 月 28 日 15:00

地点： 山西省心血管病医院医技楼 5 层小会议室

审查项目：取栓器治疗急性缺血性卒中的前瞻性、多中心、单盲、随机对照临床试验

受理编号：2018015

参加会议的伦理委员会委员名单

姓名	性别	工作单位	专业领域	职称/职务	签名
安 健	男	山西省心血管病医院	心内科	主任医师	
薄建萍	女	山西医科大学第二医院	呼吸科	主任医师	
成建国	男	山西锋镝律师事务所	法学	主任	
陈小飞	女	山西省心血管病医院	神经内科	主任医师	
郝淑兰	女	山西省中医药研究院	中医内科	主治医师	
李东芳	女	山西医科大学第二医院	神经内科	主任医师	
张亮清	男	山西省心血管病医院	心内科	心内科 7 病区主任	
李小明	男	山西省心血管病医院	心内科	主任医师	
王洪奇	男	山西医科大学	医学伦理学	教授	
王敬萍	女	山西省心血管病医院	心内科	主任医师	
郗光霞	女	山西大医院	内分泌	主任医师	
杨 滨	男	山西省心血管病医院	心内科	副主任医师	
杨会明	男	太原市迎泽区司法局	法学	副局长	
张丽贞	女	山西省心血管病医院	心内科	主任医师	
张月安	女	山西省心血管病医院	流行病与卫生统计学	科教科主任	

注：按姓氏拼音首字母排序

山西省心血管病医院药物临床试验伦理委员会

快速审查批件

审查受理编号：2018015　　　签发日期：2018 年 12 月 4 日

项目名称	取栓器治疗急性缺血性卒中的前瞻性、多中心、单盲、随机对照临床试验	
项目来源	微创神通医疗科技（上海）有限公司	
研究单位/科室	山西省心血管病医院神经外科	
主要研究者	蒯东	
审核内容	研究方案	版本号：V2.0 版本日期：20180808
	知情同意书	版本号：V2.0 版本日期：20180808
	其他文件	研究者手册版本号:V2.0 版本日期:20180808<br>病例报告表版本号:V3.0 版本日期:20180808<br>原始病历版本号：V3.0 版本日期：20180808
主审结果	伦理委员会按照国家相关法规要求，由两位主审委员对该临床试验研究进行快速审查，其结果：同意该项目在我院实施。	
跟踪审查频率	12 个月	
具体意见		
主任委员签字：[signature]	伦理委员会（盖章）<br>日期：2018.12.04	

声明：本伦理委员会组成和工作程序符合 GCP 原则及国家相关法律法规。自批件 签署日 期起1年内开始临床研究，本批件有效。

目录

1. 临床研究方案-clean 版（版本号 V2.0，版本日期 2018 年 8 月 8 日）
2. 临床研究方案-draft 版（版本号 V2.0，版本日期 2018 年 8 月 8 日）
3. 临床研究方案修订说明（V1.0→V2.0）
4. 临床研究方案修订对照列表（V1.0→V2.0）
5. 知情同意书-clean 版（版本号 V2.0，版本日期 2018 年 8 月 8 日）
6. 知情同意书-draft 版（版本号 V2.0，版本日期 2018 年 8 月 8 日）
7. 知情同意书修订对照列表（V1.0→V2.0）
8. 研究者手册-clean 版（版本号 V2.0，版本日期 2018 年 8 月 8 日）
9. 研究者手册-draft 版（版本号 V2.0，版本日期 2018 年 8 月 8 日）
10. 研究者手册修订对照列表（V1.0→V2.0）
11. 病例报告表-clean 版（版本号 V3.0，版本日期 2018 年 8 月 8 日）
12. 病例报告表-draft 版（版本号 V3.0，版本日期 2018 年 8 月 8 日）
13. 病例报告表修订对照列表（V2.0→V3.0）
14. 原始病历-clean 版（版本号 V3.0，版本日期 2018 年 8 月 8 日）
15. 原始病历-draft 版（版本号 V3.0，版本日期 2018 年 8 月 8 日）
16. 原始病历修订对照列表（V2.0→V3.0）
17. 取栓器使用说明书-clean 版（文件编号 A-T0006-002，版本号 Rev.2.0，版本日期 NA）
18. 取栓器使用说明书-draft 版（文件编号 A-T0006-002，版本号 Rev.2.0，版本日期 NA）
19. 取栓器使用说明书修订对照列表（V1.0→V2.0）
20. 组长单位伦理委员会递交信及批件
