# Supplementary material for: Endovascular treatment of acute ischemic stroke with a fully radiopaque retriever: A randomized controlled trial
Source: Front Neurol. 2022 Dec 14;13:962987. doi: 10.3389/fneur.2022.962987 (PMC9796564; doi:10.3389/fneur.2022.962987)
Supplement: Supplementary file 2 [file Data_Sheet_2.zip › 20 ╓╨╔╜╬σ╘║ .pdf]

中山大学附属第五医院医学伦理委员会

项目审批表

编号：中大五院【2018 】伦字第（Q11-1）号

项目名称	取栓器治疗急性缺血性卒中的前瞻性、多中心、单盲、随机对照临床试验			
规格型号	AIS4025、AIS6030	医疗器械类别	☐II 类，■III 类，☐体外诊断试剂	
研究科室	神经介入科	项目负责人	周斌	
申办方	微创神通医疗科技（上海）有限公司			
评审文件	见附件 1			
审查方式	会议审查	审查类型	初审	
审查日期	2018. 10. 30	审查地点	312 会议室	
会议审查情况（到会情况见附件 2）				
委员出席会议情况	应到会人数	实际到会人数	缺席人数	回避人数
	11 人	9 人	2 人	0 人
伦理委员会投票结果	同意	作必要修正后同意	作必要修正后重审	不同意
	9 票	0 票	0 票	0 票

评审意见：

经本医学伦理委员会审查，该研究符合伦理规范及相关法律法规，同意其在本中心开展。

本批件有效期至 2019 年 10 月 29 日，到期后按规定进行年度审查。

主任委员签名：

日　　期：2018 年 10 月 30 日

中山大学附属第五医院医学伦理委员会

中山大学附属第五医院医学伦理委员会

附件 1：

中山大学附属第五医院药物临床试验伦理审核资料目录

中大五院【2018】伦字第（Q11-1）号

序号	送审资料
1.	临床试验项目审议表
2.	主要研究者履历表
3.	研究者履历及分工签名表
4.	项目通讯录（申办方、CRO、参加临床试验各单位名称及联系方式）
5.	关于无国家食品药品监督管理局批件的说明
6.	组长单位批件及成员表
7.	申办方证明性文件（营业执照三证合一、医疗器械生产许可证）
8.	申办方委托函（申办方委托 CRO）
9.	CRO 公司证明性文件（营业执照三证合一）
10.	CRA 委托函、GCP 证书、毕业证书及身份证明文件
11.	试验方案（V2.0 / 2018-8-8）
12.	研究者手册（V2.0 / 2018-8-8）
13.	知情同意书文本（V2.0 / 2018-8-8）
14.	研究病历（V3.0 / 2018-8-8）
15.	病例报告表文本（V3.0 / 2018-8-8）
16.	产品检测报告
17.	产品自测报告
18.	产品注册标准
19.	对照产品注册证
20.	受试者招募说明

21.	财务规定/保险
22.	临床试验协议
23.	试验用医疗器械研制符合适用的医疗器械生产质量管理规范声明
24.	动物实验报告
25.	申办者保证所提供材料真实性的声明
26.	说明书

编号：中大五院【2018】伦字第（Q11-1）号

附件 2：医学伦理委员会会议审查签到表

项目名称		取栓器治疗急性缺血性卒中的前瞻性、多中心、单盲、随机对照临床试验			
伦理委员会委员名单		性别	专业	单位	签名
主任委员	黄　瑾	男	医学	中山大学附属第五医院	[illegible]
副主任委员	李绍林	男	医学	中山大学附属第五医院	
副主任委员	温盛霖	男	医学	中山大学附属第五医院	[illegible]
委员	于翠香	女	护理	中山大学附属第五医院	[illegible]
	王爱红	女	法律	广东文证律师事务所	[illegible]
	卢华定	男	医学	中山大学附属第五医院	[illegible]
	张卫华	女	工程	珠海水务集团	[illegible]
	林　忠	男	医学	中山大学附属第五医院	[illegible]
	陈红涛	女	医学	中山大学附属第五医院	
	曹庆东	男	医学	中山大学附属第五医院	[illegible]
	梁嘉碧	女	药学	中山大学附属第五医院	[illegible]

注意事项：修正后同意/重审项目，应将修正后文件及时反馈给伦理委员会，以便安排再审（包括快审）。

1.修正后同意/重审项目，应将修正后文件及时反馈给伦理委员会，以便安排再审（包括快审）。

2.不同意项目可在 2 周内向伦理委员会就有关事项做出解释或提出申诉。

3.参与试验的单位，应严格按照本伦理委员会批准的方案执行；如有必须做出修改的建议，应形成书面文件并及时与本伦理委员会沟通协商。

4.对已批准的临床研究方案、知情同意书等材料的任何修改及主要研究者的更换等，须及时通知本伦理审查委员会并提交更改申请及相关资料，经重新审查，获得批准后方可执行。

5.试验中发生任何严重不良事件及可能影响风险收益比的任何事件和新信息，须及时报告本伦理委员会。

6. 定期/年度跟踪审查项目，于到期前 2 周提交试验进展情况报告。

7. 如有不依从/违背方案或暂停/提前终止的试验项目，应及时通知本伦理审查委员会；临床试验结束时，须及时向本伦理委员会提交结题报告。

8.本批件自发出后一年内有效，如试验逾期未实施即自行废止；如试验开展一年以上，需向本委员会提交试验年度报告。

9.本委员会联系电话：0756-2528895　　联系人：宋蕾

声明：本伦理审查委员会按照 GCP 和有关法规组成和工作，其审查和工作过程不受伦理审查委员会以外组织及个人影响。

由 扫描全能王 扫描创建
