# Supplementary material for: Endovascular treatment of acute ischemic stroke with a fully radiopaque retriever: A randomized controlled trial
Source: Front Neurol. 2022 Dec 14;13:962987. doi: 10.3389/fneur.2022.962987 (PMC9796564; doi:10.3389/fneur.2022.962987)
Supplement: Supplementary file 2 [file Data_Sheet_2.zip › 21 ─╧╤⌠╢■.pdf]

南阳市第二人民医院伦理委员会批准函

首次伦理审查<br>批准编号	械-2019001	首次伦理审查<br>批准日期	2019-10-22
研究方案名称/项目编号	取栓器治疗急性缺血性卒中的前瞻性、多中心、单盲、随机对照临床试验/2016-GATOR-01-A		
申办者	微创神通医疗科技（上海）有限公司	CFDA 批件	□有 □无 ■不适用
CRO	方恩（天津）医药发展有限公司	研究期别	□Ⅰ期 □Ⅱ期 □Ⅲ期 □Ⅳ期 □登记研究<br>□临床科研 ■器械临床验证 □器械临床试用
试验器械名称及型号	取栓器/AIS4025、AIS6030		
受理审查文件	1.临床研究方案 版本号：V2.0 版本日期：2018-08-08<br>2.知情同意书 版本号：V2.0 版本日期：2018-08-08<br>3.研究者手册 版本号：V2.0 版本日期：2018-08-08<br>4.病例报告表 版本号：V3.0 版本日期：2018-08-08<br>5.主要研究者简历<br>6.临床试验的设施条件能够满足实验的综述<br>7.取栓器说明书及对照产品说明书<br>7.研究产品自测报告，注册检验合格报告<br>9.组长单位伦理批件<br>10.企业法人营业执照<br>11.医疗器械生产企业许可证<br>12. 复核通过注册产品标准或相应的国家、行业标准<br>13.器械销毁委托书<br>14.申办者声明<br>15.申办方说明性文件<br>16.取栓器招募说明 2017-03-10<br>17.CRO 资质<br>18.CRO 委托书<br>19.保险<br>20.其他申办方新版授权书		
主要研究者及职称	李新满/主任医师	研究单位及专业	南阳市第二人民医院脑血管介入科
审查方式	□会议审查 □快速审查 □紧急会议审查 ■备案		

2019 年 9 月 27 日微创神通医疗科技（上海）有限公司向南阳市第二人民医院伦理委员会递交了试验相关资料，伦理委员会对送审材料进行审查和讨论，**本次审批意见如下：同意在我院按照本次递交试验方案进行该临床试验研究。**

1、请按照 GCP 原则和审查通过的递交材料：试验方案（版本号：V2.0，版本日期：20180808）、知情同意书（版本号：V2.0，版本日期：20180808）进行临床试验研究。

2、在开展该项研究时要求，上述审评资料未经本委员会批准，不得作任何修改；如果研究中发生严重不良事件请立即（24 小时内）通知本委员会。

3、如果试验开展一年以上，需向本委员会提交试验年度报告并接受年度跟踪审查及再备案。

4、该研究进行过程中将接受伦理委员会的持续审查，伦理委员会有权根据实际进展情况改变持续审查频率。

5、批件超过有效期请提前 1 个月提出申请。

主任委员签字：

（伦理委员会盖章）

2019 年 10 月 22 日

年度/定期跟踪审查频率：□3 个月 □6 个月 ■12 个月	此批件有效期至：2020 年 10 月 21 日

声明：

1.本院伦理委员会职责、人员组成，操作规程及记录均遵循 ICH-GCP、CFDA-GCP、中国相关法规。

2.“同意”的研究应遵循已经伦理委员会批准的方案执行，应符合 ICH-GCP、CFDA-GCP 和《赫尔辛基宣言》的原则。

3.“作必要的修正后同意”的研究请按评审意见进行逐条修改并递交临床研究文件修正申请及新版本文件，申请经伦理委员会同意后，方可获得批准编号。

4.“作必要的修正后重审”的研究请按评审意见进行修改，并递交临床研究文件修正申请及新版本文件，在下次全体会议中审查，由全体委员投票决定结果。

5.“不同意”或“暂停或终止已经批准的临床试验”的研究方案，申办者和研究者有权就伦理委员会的意见和建议中提及的问题做书面申诉，由全体委员投票决定结果。

6.本中心发生的严重不良事件或意外不良事件需在向 CFDA 上报的同时向伦理委员会作书面报告，必要时会邀请研究者参加全体会议进行审查，伦理委员会有权根据对其的评估并做出新的决定。

7.无论试验开始与否，请在下次持续审查已到期前 1 个月提出持续审查的申请。

8.南阳市第二人民医院伦理委员会

地址：南阳市建设东路 66 号南阳市第二人民医院，邮编：473000

秘书处联系人：徐鹏　联系电话：0377-61609960

E-mail: nyseyywk@163.com
