# Supplementary material for: Endovascular treatment of acute ischemic stroke with a fully radiopaque retriever: A randomized controlled trial
Source: Front Neurol. 2022 Dec 14;13:962987. doi: 10.3389/fneur.2022.962987 (PMC9796564; doi:10.3389/fneur.2022.962987)
Supplement: Supplementary file 2 [file Data_Sheet_2.zip › 22 └÷╦«.pdf]

伦理审查批件

伦审号：2019-伦理审查-22

伦理审查日期	2019 年 12 月 18 日
试验项目名称	取栓器治疗急性缺血性卒中的前瞻性、多中心、单盲、随机对照临床试验
申 办 方	微创神通医疗科技（上海）有限公司
专业组/主要研究者	神经内科/蔡学礼
审查方式	☑ 会议审查　　□快速审查

到会委员人数：应到人数 13 人，实到人数 8 人，回避人数 0 人。

投票结果：同意 8 票，修改后同意 0 票，修改后重审 0 票，不同意 0 票。

审查意见：

根据国家卫生计生委《涉及人的生物医学研究伦理审查办法》（2016）、SFDA《药物临床试验质量管理规范》（2003）、CFDA《医疗器械临床试验质量管理规范》（2016）、WMA《赫尔辛基宣言》和 CIOMS《人体生物医学研究国际道德指南》的伦理原则，经本伦理委员会审查，同意按所批准的临床研究方案、知情同意书开展本项研究。请遵循 ICH-GCP、GCP 原则，保护受试者的健康和权利。

此项研究的审查频率是：□3 个月　□6 个月　☑12 个月。

注意事项：

所有资料未经委员会审核批准，不得作任何修改；

如果试验中发生任何严重不良事件请在获知后 24 小时内通知本伦理委员会；

试验过程中若发生违背试验方案，及时提交违背方案报告；

按规定的定期跟踪审查频率，请在审查日期前 1 月递交跟踪审查申请及报告；

从批准之日起，每一年度需向伦理委员会提交年度报告，请在审查日期前 1 个月递交年度审查的申请及报告；

暂停或提前终止临床研究，及时提交暂停/终止审查申请；

研究结束时，应向伦理委员会递交结题报告。

主任委员/副主任委员签名：[signature]　　　日期：2019 年 12 月 30 日

丽水市中心医院伦理委员会临床试验伦理小组（盖章）

[Stamp: 丽水市中心医院伦理委员会 临床试验伦理小组]

伦理委员会地址： 丽水市括苍路 289 号　　联系方式：0578-2285102/2285719

伦理委员会审查文件目录：

1. 初始审查申请表
2. 临床试验方案（版本号：V2.0；版本日期：2018-08-08）
3. 研究者手册（版本号：V1.0；版本日期：2017-03-08）
4. 知情同意书（版本号：V3.0；版本日期：2019-11-20）
5. 招募流程说明
6. 病例报告表(版本号：V1.0；版本日期：2017-11-06)
7. 自检报告和产品注册检验报告（报告编号：国医检（械）字 ZC2016 第 636 号）
8. 研究者简历、专业特长、能力、接受培训和其他能够证明其资格的文件
9. 临床试验机构的设施和条件能够满足试验的综述
10. 试验用医疗器械的研制符合适用的医疗器械质量管理体系相关要求的声明
11. 牵头单位（上海长海医院）伦理委员会批件（批件号：CHEC2018-124）

以下无正文

伦理委员会会议签到表

丽水市中心医院伦理委员会 临床试验伦理小组

会议地点	丽水市中心医院行政楼三楼党员活动室
会议日期	2019 年 12 月 18 日 14：00

姓　名	性别	职称	职务	专业背景	工作单位	签　名
曾春来	男	主任医师	主任委员	心血管内科	丽水市中心医院	曾春来
吕耀军	男	高级政工师	副主任委员	党政管理	丽水市中心医院	吕耀军
魏以新	男	主任医师	副主任委员	心血管内科	丽水市中心医院	—
方伟钧	男	主任医师	委员	急危重症科	丽水市中心医院	—
徐宏涛	男	主任医师	委员	普通外科	丽水市中心医院	—
赵中伟	男	~~副~~主任医师	委员	医学影像科	丽水市中心医院	赵中伟
谢艳茹	女	~~副~~主任医师	委员	肿瘤内科	丽水市中心医院	谢艳茹
李　雅	女	主任医师	委员	消化内科	丽水市中心医院	李雅
周丽红	女	主任医师	委员	妇产科	丽水市中心医院	—
骆松梅	女	主任药师	委员	药学部	丽水市中心医院	—
季伟艺	男	经济师	委员秘书	法学	丽水市中心医院	季伟艺
陈　超	男	四级律师	委员	律师	浙江晟耀律师事务所	陈超
叶前	女	无	委员	中西医临床医学	丽水市科技局	叶前
