# Supplementary material for: Endovascular treatment of acute ischemic stroke with a fully radiopaque retriever: A randomized controlled trial
Source: Front Neurol. 2022 Dec 14;13:962987. doi: 10.3389/fneur.2022.962987 (PMC9796564; doi:10.3389/fneur.2022.962987)
Supplement: Supplementary file 2 [file Data_Sheet_2.zip › 23 ─╧▓2╢■.pdf]

南昌大学第二附属医院临床试验伦理委员会审查批件

IEC Approval Letter of the second affiliated hospital of Nanchang university

审批号：器临审【2019】第（19)号

项目名称<br>Project Title	取栓器治疗急性缺血性卒中的前瞻性、多中心、单盲、随机对照临床试验		
审查时间<br>Review time	2020.01.08	审查地点<br>Review site	综合楼13楼伦理委员会办公室
研究类别<br>Category of research	器械临床试验		
申办者<br>Sponsor	微创神通医疗科技（上海）有限公司		
专业组<br>Specialty	神经外科	主要研究者<br>Principal Investigator	祝新根
审查形式<br>Review modes	会议审查	审查类别<br>Review category	初始审查
审查文件<br>Review documents	(AIS4025)取栓器检验报告（批号：C.2D.07A0001）；(AIS6030)取栓器检验报告（批号：C.2D.06A0003）；取栓器自测报告；临床研究方案（版本号：V2.0，版本日期：2018.08.08）；知情同意书（版本号：V3.0，版本日期：2019.11.20）；受试者招募流程说明；医疗器械临床试验病例报告表（版本号：V3.0，版本日期：2018.08.08）；原始病历（版本号：V3.0，版本日期：2018.08.08）；研究者手册（版本号：V2.0，版本日期：2018.08.08）；GLP 实验室对微创神通取栓器在猪动物模型的治疗评估总结报告；保险凭证（安达保险，92666573）；主要研究者履历表及试验人员团队分工表		
伦理委员会投票结果汇总<br>Results of IEC			
共 13 名委员参加会议，其中 13 名委员投票，0 名委员回避；<br>同意 12 票，作必要修正后同意 1 票，作必要修正后重审 0 票，不同意 0 票，终止或暂停已经批准的临床试验 0 票			
审查结果<br>Review decisions	同意，持续审查频率为12个月		
注意<br>Attention	1、 研究过程中对若变更主要研究者，对研究资料有任何修改，请申请人提交修正案审查申请。<br>2、 如发生严重不良事件，请申请人按要求提交严重不良事件报告。<br>3、 根据伦理委员会对跟踪审查频度的意见，无论试验开始与否，请申请人在跟踪审查到期前一个月提出跟踪审查的申请，递交项目进展报告。<br>4、 如发生违背方案的情况，请申办方\监察员\研究者提交违背方案报告。<br>5、 申请人暂停或提前终止临床研究，请及时提交暂停/终止研究报告。<br>6、 如项目涉及人体生物样本外送至境外实验室检测，待获得“中国人类遗传资源管理办公室”批准后将批件递交医学伦理委员会备案才可开展外送程序。<br>7、 研究结束请申请人按要求提交结题报告。<br>8、 本批件有效期为一年，逾期未实施的，则自行废止。		

主任委员签字：程[illegible]新

日期：2020.11.10

南昌大学第二附属医院临床试验伦理委员会（盖章）

声明：本伦理委员会严格按照中国 GCP 及相关法规组成和工作

地址：南昌市民德路1号电话&传真：（0791）86209562 邮箱：efyiec@163.com

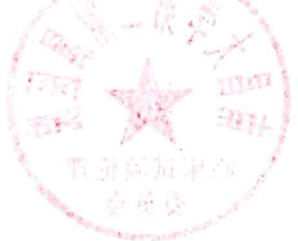
委员会

南昌大学第二附属医院临床试验伦理委员会　　AF/SC-04/2.0

南昌大学第二附属医院临床试验伦理委员会审查批件

IEC Approval Letter of the second affiliated Hospital of Nanchang university

主任委员签字：

审批号：药临审【2019】第（50）号

日期：2020.1.10

南昌大学第二附属医院临床试验伦理委员会（盖章）

声明：本伦理委员会严格按照中国 GCP 及相关法规组成和工作

地址：南昌市民德路 1 号电话&传真：(0791 [illegible] 邮箱：efyiec@163.com

项目名称<br>Project Title	一项在血糖控制不佳的中国 2 型糖尿病患者中开展的采用恩格列净（10mg 和 25mg）每日一次口服联合胰岛素不伴或伴有最多 2 种口服降糖[illegible]安慰剂对照、平行分组研究		
审查时间<br>Review time	2020.01.08	审查地点<br>Review site	综合楼 13 楼伦理委员会办公室
研究类别<br>Category of research	药物临床试验		
申办者<br>Sponsor	勃林格殷格翰（中国）投资有限公司		
专业组<br>Specialty	内分泌科	主要研究者<br>Principal Investigator	赖晓阳
审查形式<br>Review modes	会议审查	审查类别<br>Review category	初始审查
审查文件<br>Review documents	临床试验通知书（JXHL1900167、JXHL1900168）；恩格列净（10mg）薄膜包衣片（批号：707210）；恩格列净（25mg）薄膜包衣片（批号：707331）；BI 10773（10mg）薄膜包衣片安慰剂（批号：B171003521）；BI 10773（25mg）薄膜包衣片安慰剂（批号：B171003250）；临床试验方案中英文版（版本号：1.0，版本日期：2019.06.06）；试验受试者须知和同意书（版本日期：2019.09.23）；患者招募广告（版本号：V1.0，版本日期：2019.06.26）；关于 1245-0191 试验招募广告传播形式的说明；临床试验识别卡（版本号：V1.0，版本日期：2019.04.25）；（V2、V3、V4、V5、V6、V7）8 点法血糖监测日志（版本号：2.0，版本日期：2019.08.05）；（V2、V8）每日家庭血糖监测日志（版本号：1.0，版本日期：2019.06.26）；（V3、V4/5、V6/7）每周家庭血糖监测日志（版本号：1.0，版本日期：2019.06.26）；病例报告表（版本日期：2019.09.13）；研究者手册（版本号：20，版本日期：2019.01.11）；保险凭证（京东安联财产保险，201-1-310-19-000021-000-00）；中心实验室委托说明；项目风险的预评估及风险处置预案；主要研究者履历表及试验人员团队分工表		
伦理委员会投票结果汇总<br>Results of IEC			
共 13 名委员参加会议，其中 13 名委员投票，0 名委员回避；<br>同意 13 票，作必要修正后同意 0 票，作必要修正后重审 0 票，不同意 0 票，终止或暂停已经批准的临床试验 0 票			
审查结果<br>Review decisions	同意，持续审查频率为 12 个月		
注意<br>Attention	1、 研究过程中对若变更主要研究者，对研究资料有任何修改，请申请人提交修正案审查申请。<br>2、 如发生严重不良事件，请申请人按要求提交严重不良事件报告。<br>3、 根据伦理委员会对跟踪审查频度的意见，无论试验开始与否，请申请人在跟踪审查到期前一个月提出跟踪审查的申请，递交项目进展报告。<br>4、 如发生违背方案的情况，请申办方\监察员\研究者提交违背方案报告。<br>5、 申请人暂停或提前终止临床研究，请及时提交暂停/终止研究报告。<br>6、 如项目涉及人体生物样本外送至境外实验室检测，待获得“中国人类遗传资源管理办公室”批准后将批件递交医学伦理委员会备案才可开展外送程序。<br>7、 研究结束请申请人按要求提交结题报告。<br>8、 本批件有效期为一年，逾期未实施的，则自行废止。		

会 议 签 到 表

Signature of Full Borad Meeting

时间：2020 年 01 月 08 日

姓 名<br>Name	性别<br>Gender	工 作 单 位<br>Work Unit	专 业<br>Speciality	职称<br>Technical Post	职务<br>position	签 名<br>Signature
程学新<br>Xuexin Cheng	男<br>Male	南昌大学第二附属医院<br>The second affiliated hospital of Nanchang university	医疗管理<br>Hospital Administration	教授<br>Professor	主任委员<br>Chairman	
葛晓珍<br>Xiaozhen Ge	女<br>Female	南昌大学第二附属医院<br>The second affiliated hospital of Nanchang university	医疗管理<br>Hospital Administration	主任技师<br>Chief Technician	副主任委员<br>Associate Chairman	
李 剑<br>Jian Li	女<br>Female	南昌大学第二附属医院<br>The second affiliated hospital of Nanchang university	血液学<br>Hematology	教授<br>Professor	委员<br>Committee member	
杨维兰<br>Weilan Yang	女<br>Female	南昌大学第二附属医院<br>The second affiliated hospital of Nanchang university	医疗管理<br>Hospital Administration	主任医师<br>Chief physician	委员<br>Committee member	
吴清华<br>Qinghua Wu	男<br>Male	南昌大学第二附属医院<br>The second affiliated hospital of Nanchang university	心血管内科<br>Cardiology	主任医师<br>Chief physician	委员<br>Committee member	
洪 葵<br>Kui Hong	女<br>Female	南昌大学第二附属医院<br>The second affiliated hospital of Nanchang university	心血管内科<br>Cardiology	主任医师<br>Chief physician	委员<br>Committee member	
唐燕华<br>Yanhua Tang	女<br>Female	南昌大学第二附属医院<br>The second affiliated hospital of Nanchang university	心胸外科<br>Cardio-thoracic surgery	主任医师<br>Chief physician	委员<br>Committee member	

涂江龙<br>anglong Tu	男<br>Male	南昌大学第二附属医院<br>The second affiliated hospital of Nanchang university	神经内科<br>Neurology	副主任医师<br>Associate chief physician	委员<br>Committee member	
刘晶美<br>ingmei Liu	女<br>Female	南昌大学第二附属医院<br>The second affiliated hospital of Nanchang university	消化内科<br>Gastroenterology	主任医师<br>Chief physician	委员<br>Committee member	
况九龙<br>long Kuang	男<br>Male	南昌大学第二附属医院<br>The second affiliated hospital of Nanchang university	呼吸内科<br>Respiratory	主任医师<br>Chief physician	委员<br>Committee member	
黄 龙<br>ong Huang	男<br>Male	南昌大学第二附属医院<br>The second affiliated hospital of Nanchang university	肿瘤学<br>Oncology	主治医师<br>Attending physician	委员<br>Committee member	
雷立生<br>sheng Lei	男<br>Male	南昌大学第二附属医院<br>The second affiliated hospital of Nanchang university	经济学<br>Economics	高级会计师<br>Senior accountant	委员<br>Committee member	
张 红<br>ong Zhang	女<br>Female	南昌大学<br>Nanchang university	临床药理学<br>Clinical pharmacology	教授<br>Professor	委员<br>Committee member	
王 卿<br>ing Wang	女<br>Female	江西华邦律师事务所<br>Jiangxi Huabang law firm	法学<br>Law	律 师<br>lawyer	委员<br>Committee member	
王棠棣<br>ngdi Wang	女<br>Female	南昌市东湖区妇联<br>East Lake District Women's Federation	社区工作者<br>Community workers	主席<br>Chairman	委员<br>Committee member	
