# Supplementary material for: Endovascular treatment of acute ischemic stroke with a fully radiopaque retriever: A randomized controlled trial
Source: Front Neurol. 2022 Dec 14;13:962987. doi: 10.3389/fneur.2022.962987 (PMC9796564; doi:10.3389/fneur.2022.962987)
Supplement: Supplementary file 2 [file Data_Sheet_2.zip › 16 ─╧╖╜╥╜╘║.pdf]

南方医院 南方医科大学南方医院医学伦理委员会

编码：AF/SC-08/03.2

伦理审查意见

意见号	NFEC-201803-Q1-01		
研究项目名称	取栓器治疗急性缺血性卒中的前瞻性、多中心、单盲、随机对照临床试验		
申请者	微创神通医疗科技（上海）有限公司		
研究科室	南方医科大学南方医院神经内科		
主要研究者	潘速跃		
审查类别	初始审查（器械临床试验）		
审查方式	■会议审查　☐快速审查		
审查日期	2018-3-15	审查地点	医学伦理会议室
审查委员	张训（主任医师），郭志刚（主任医师），汤明芳（女，主任医师），薛莲（女，助理研究员），刘世霆（主任药师），冯茹（女，主任医师），吴炳义（教授），吴志华（副教授），全松（主任医师），刘浩（副主任医师），沈少林（律师），严金海（非医药专业，外单位）		
审查文件	1、递交信<br>2、初始审查申请<br>3、主要研究者简历<br>4、主要研究者责任声明<br>5、研究人员职责签名表<br>6、所有研究者的 GCP 培训证书复印件<br>7、临床研究方案（1.0 版 2017 年 3 月 8 日）<br>8、知情同意书（2.0 版 2018 年 2 月 22 日）<br>9、病例报告表（2.0 版 2017 年 11 月 6 日）<br>10、原始病历（2.0 版 2017 年 11 月 6 日）<br>11、保险证明<br>12、医疗器械产品自测报告<br>13、产品技术要求<br>14、注册检验报告（含临床预评价意见）<br>15、医疗器械研制符合适用的医疗器械质量管理体系相关要求的声明<br>16、动物试验报告<br>17、研究者手册（1.0 版 2017 年 3 月 8 日）<br>18、申办者和 CRO 资质证明<br>19、所有以前其他伦理委员会对该研究项目的重要决定<br>20、风险预案<br>21、其它(产品说明书/产品储存温湿度说明/销毁委托书/新增中心说明）		

审查意见	作必要的修正后同意，跟踪审查频率：12 个月<br>1. 增加伦理委员会的联系方式，研究者姓名与联系方式；<br>2. 明确免费器械的费用总和；<br>3. 去掉“良好的”诱导性词句。
主任委员或副主任委员签名：张[illegible]<br>南方医科大学南方医院医学伦理委员会（盖章）<br>日　　期：2018.3.23	
备注：<br>1.按审查意见修改后的文件，或对审查意见不同观点的陈诉，请提交“复审申请”，方案/知情同意书请注明新的版本号和版本日期，并以阴影和（或）下划线方式标注修改部分，报伦理委员会审查，经批准后执行。<br>2.不同意/暂停或终止项目，2 周内可向伦理委员会就有关事项做出解释或提出申诉。	

联系方式：广州市广州大道北 1838 号，邮编：510515
电话/传真：020-62787238/87713945，邮箱：nfyyec@163.com

会议签到表

会议时间：2018 年 3 月 15 日（星期 四 ） 下 午 14 时 30 分

会议地点：医学新实验楼十六楼伦理会议室

伦理任职	姓　名	工作单位	性别	职　称	专　业	签　名
主任委员	张　训	南方医科大学南方医院肾内科	男	主任医师/教授	内科学（肾脏病学）	张训
副主任委员	郭志刚	南方医科大学南方医院心血管内科	男	主任医师/教授	心血管内科学	郭志刚
委员秘书	汤明芳	南方医科大学南方医院眼科	女	主任医师/副教授	眼科学	汤明芳
	薛莲	南方医科大学南方医院科研处	女	助理研究员	卫生事业管理	薛莲
委员	鲁　鸿	南方医科大学南方医院	女	副教授	卫生事业管理学	
	刘世霆	南方医科大学南方医院药学部	男	主任药师/教授	药理学	[illegible]
	冯茹	南方医科大学南方医院血液科	女	主任医师/教授	血液病	[illegible]
	吴炳义	南方医科大学临床医学实验研究中心	男	主任/教授	临床医疗	[illegible]
	周宏珍	南方医科大学南方医院护理部	女	主任护师	护理学	
	吴志华	南方医院科研处	男	处长/副教授	科研管理	[illegible]
	全松	南方医科大学南方医院妇产科	男	副主任/ 教授、主任医师	生殖医学	全松
	孙剑	南方医科大学南方医院感染内科	男	副主任/教授、主任医师	内科学（传染病）	
	刘浩	南方医科大学南方医院普外科	男	副主任医师、副教授	普通外科	刘浩
	沈少林	广东君之杰律师事务所	男	教授/律师	法律	沈少林
	严金海	南方医科大学人文社科学院	男	教授	哲学	严金海

伦理审查批件 Approval Letter

批件号 No.	NFEC-2018-040
研究项目名称 Protocol Title	取栓器治疗急性缺血性卒中的前瞻性、多中心、单盲随机对照临床试验
申请者 Submitter	微创神通医疗科技（上海）有限公司
研究科室 Research department	神经内科
主要研究者 Principal Investigator	潘速跃
审查类别 Scope of review	复审（器械临床试验）
审查方式 Review mode	☐会议审查 Full board review ■快速审查 Expedited review
审查日期 Review Date	2018-4-17
审查地点 Review Place	NA
审查委员 Review members	冯茹（主任医师，女），严金海（非医药专业，外单位）
审查文件 Documents	1、递交信<br>2、初始审查申请<br>3、主要研究者简历<br>4、主要研究者责任声明<br>5、研究人员职责签名表<br>6、所有研究者的 GCP 培训证书复印件<br>7、临床研究方案（1.0 版 2017 年 3 月 8 日）<br>8、知情同意书（V2.0 版 2018 年 3 月 27 日）<br>9、病例报告表（2.0 版 2017 年 11 月 6 日）<br>10、原始病历（2.0 版 2017 年 11 月 6 日）<br>11、保险证明<br>12、医疗器械产品自测报告<br>13、产品技术要求<br>14、注册检验报告（含临床预评价意见）<br>15、医疗器械研制符合适用的医疗器械质量管理体系相关要求的声明<br>16、动物试验报告<br>17、研究者手册（1.0 版 2017 年 3 月 8 日）<br>18、申办者和 CRO 资质证明<br>19、所有以前其他伦理委员会对该研究项目的重要决定<br>20、风险预案<br>21、其它(产品说明书/产品储存温湿度说明/销毁委托书/新增中心说明）<br>22、复审申请表
审查意见 Comments	同意。

年度/定期跟踪审查频率<br>Annual Follow-Up Review Frequency	□3 个月 3 Months　□6 个月 6 Months　■12 个月 12 Months
批件有效期<br>Expiry of Approval Letter	三年

主任委员或副主任委员签名：张[illegible]
Signature of the Chair or Vice-chair
南方医科大学南方医院医学伦理委员会（盖章）
Medical Ethics Committee of Nanfang Hospital(Seal)
日期：
Date: 2018. 4. 17

注意事项：

1. 本批件可能在其他中心机构及其伦理委员会备案。如果对方案在贵机构的可行性（包括研究者的资格与经验、设备与条件等）有不同意见，请及时与本伦理委员会联系。
2. 请遵循 CFDA/GCP，《药物临床试验伦理审查工作指导原则》和《赫尔辛基宣言》的原则、遵循伦理委员会批准的方案开展临床研究，保护受试者的健康与权利。
3. 研究过程中若变更主要研究者，对临床研究方案、知情同意书、招募材料等的任何修改，请申请人提交修正案审查申请。
4. 发生严重不良事件，请申请人及时提交严重不良事件报告。
5. 请按照伦理委员会规定的年度定期跟踪审查频率，申请人在截止日期前 1 个月提交年度定期跟踪审查报告;申办者应当向组长单位伦理委员会提交各中心研究进展的汇总报告;当出现任何可能显著影响试验进行或增加受试者危险的情况时，请申请人及时向伦理委员会提交书面报告。
6. 研究纳入了不符合纳入标准或符合排除标准的受试者，符合中止试验规定而未让受试者退出研究，给予错误治疗或剂量，给予方案禁止的合并用药等没有遵从方案开展研究的情况；或可能对受试者的权益健康以及研究的科学性造成不良影响等违背 GCP 原则的情况，请申办者/监查员/研究者提交违背方案报告。
7. 申请人暂停或提前终止临床研究，请及时提交暂停/终止研究报告。
8. 完成临床研究，请申请人提交结题报告。

声明：本伦理委员会按照中国 GCP、ICH GCP 和有关法规组成和工作，其审查和工作过程不受任何组织及个人的影响

联系方式：广州市广州大道北 1838 号，邮编：510515
电话/传真：020-62787238/87713945
邮箱：nfyyec@163.com

伦理审查意见

意见号	NFEC-201803-Q1-修正案审查 01		
研究项目名称	取栓器治疗急性缺血性卒中的前瞻性、多中心、单盲、随机对照临床试验		
申请者	微创神通医疗科技（上海）有限公司		
研究科室	神经内科		
主要研究者	潘速跃		
审查类别	跟踪审查（修正案审查）		
审查方式	■会议审查　　☐快速审查		
审查日期	2018-11-22	审查地点	伦理委员会会议室
审查委员	张训（主任医师），全松（主任医师），郭志刚（主任医师），严金海（非医药专业，外单位），刘浩（副主任医师），周宏珍（女，主任护师），汤明芳（女，主任医师），沈少林（律师），孙剑（主任医师），薛莲（女，助理研究员），吴炳义（教授）		
审查文件	1. 修正案审查申请<br>2. 组长单位伦理批件<br>3. 临床研究方案（版本号：V2.0；版本日期：2018-08-08）<br>4. 临床研究方案【修改说明】含补充说明<br>5. 临床研究方案【修订列表】<br>6. 临床研究方案【痕迹修订版】<br>7. 知情同意书（版本号：V3.0；版本日期：2018-09-09）<br>8. 知情同意书【修订列表】<br>9. 知情同意书【痕迹修订版】<br>10. 研究病历（版本号：V3.0，日期：2018-08-08）<br>11. 研究病历【修订列表】<br>12. 研究病历【痕迹修订版】<br>13. 病例报告表（CRF/eCRF）（版本号：V3.0，日期：2018-08-08）<br>14. 病例报告表（CRF/eCRF）【修订列表】<br>15. 病例报告表（CRF/eCRF）【痕迹修订版】		

审查文件	16. 医疗器械研究者手册（版本号：V2.0，日期：2018-08-08）<br>17. 医疗器械研究者手册【修订列表】<br>18. 医疗器械研究者手册【痕迹修订版】<br>19. 取栓器说明书（版本号：V2.0）<br>20. 取栓器说明书【修订列表】<br>21. 取栓器说明书【痕迹修订版】
审查意见	同意执行修正案，跟踪审查频率不变（12 个月）。
主任委员或副主任委员签名：张[illegible]<br>南方医科大学南方医院医学伦理委员会（盖章）<br>日期：2018.12.4	
备注：<br>1.按审查意见修改后的文件，或对审查意见不同观点的陈诉，请提交"复审申请"，方案/知情同意书请注明新的版本号和版本日期，并以阴影和（或）下划线方式标注修改部分，报伦理委员会审查，经批准后执行。<br>2.不同意/暂停或终止项目，2 周内可向伦理委员会就有关事项做出解释或提出申诉。	

联系方式：广州市广州大道北 1838 号，邮编：510515
电话/传真：020-62787238/87713945，邮箱：nfyyec@163.com
