# Supplementary material for: Endovascular treatment of acute ischemic stroke with a fully radiopaque retriever: A randomized controlled trial
Source: Front Neurol. 2022 Dec 14;13:962987. doi: 10.3389/fneur.2022.962987 (PMC9796564; doi:10.3389/fneur.2022.962987)
Supplement: Supplementary file 3 [file Data_Sheet_3.docx]

**The names and affiliation of each ethics committee**

Shanghai Changhai Hospital ethics committee

The First Affiliated Hospital of Zhengzhou University ethics committee

Changzhou First People's Hospital ethics committee

Qilu Hospital of Shandong University ethics committee

Shanghai Oriental Hospital ethics committee

The Third People's Hospital of Hubei Province ethics committee

Zhejiang Provincial People's Hospital ethics committee

The First People's Hospital of Jining City ethics committee

Zhangzhou Hospital Affiliated to Fujian Medical University ethics committee

Nanjing Gulou Hospital ethics committee

The Affiliated Hospital of Qingdao University ethics committee

Liaocheng People's Hospital Brain Hospital ethics committee

Zibo Central Hospital ethics committee

Nanfang Hospital of Southern Medical University ethics committee

The Second Affiliated Hospital of Air Force Military Medical University ethics committee

Hangzhou First People's Hospital ethics committee

Shanxi Provincial Cardiovascular Hospital ethics committee

The Fifth Affiliated Hospital of Sun Yat-sen University ethics committee

Nanyang Second People's Hospital ethics committee

The Second Affiliated Hospital of Nanchang University ethics committee

Lishui Municipal Central Hospital ethics committee
